# Heterologous Expression of a Cryptic BGC from *Bilophila* sp. Provides Access to a Novel Family of Antibacterial Thiazoles

**DOI:** 10.1021/acssynbio.5c00042

**Published:** 2025-02-25

**Authors:** Maximilian Hohmann, Denis Iliasov, Martin Larralde, Widya Johannes, Klaus-Peter Janßen, Georg Zeller, Thorsten Mascher, Tobias A. M. Gulder

**Affiliations:** † Chair of Technical Biochemistry, TUD Dresden University of Technology, Bergstraße 66, 01069 Dresden, Germany; ‡ General Microbiology, TUD Dresden University of Technology, Zellescher Weg 20b, 01217 Dresden, Germany; § Leiden University Center for Infectious Diseases (LUCID), Leiden University Medical Center, 2333 ZA Leiden, Netherlands; ∥ Department of Surgery, School of Medicine and Health, Klinikum Rechts der Isar, Technical University of Munich, 81675 Munich, Germany; ⊥ Leiden University Center for Infectious Diseases (LUCID) and Center for Microbiome Analyses and Therapeutics (CMAT), Leiden University Medical Center, 2333 ZA Leiden, Netherlands; ∇ Department of Natural Product Biotechnology, Helmholtz Institute for Pharmaceutical Research Saarland (HIPS), Helmholtz Centre for Infection Research (HZI) and Department of Pharmacy, PharmaScienceHub (PSH), Saarland University, Campus E8.1, 66123 Saarbrücken, Germany

**Keywords:** bilothiazoles, *Bilophila* sp., gut mircrobiome, natural products, antibiotics, DiPaC

## Abstract

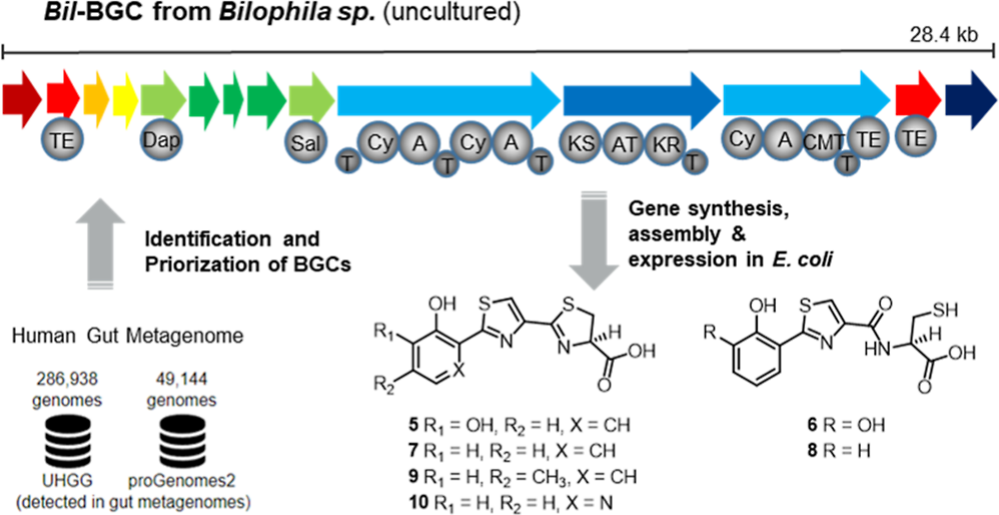

Human health is greatly influenced by the gut microbiota and microbiota imbalance can lead to the development of diseases. It is widely acknowledged that the interaction of bacteria within competitive ecosystems is influenced by their specialized metabolites, which act, e.g., as antibacterials or siderophores. However, our understanding of the occurrence and impact of such natural products in the human gut microbiome remains very limited. As arylthiazole siderophores are an emerging family of growth-promoting molecules in pathogenic bacteria, we analyzed a metagenomic data set from the human microbiome and thereby identified the *bil*-BGC, which originates from an uncultured *Bilophila* strain. Through gene synthesis and BGC assembly, heterologous expression and mutasynthetic experiments, we discovered the arylthiazole natural products bilothiazoles A–F. While established activities of related molecules indicate their involvement in metal-binding and -uptake, which could promote the growth of pathogenic strains, we also found antibiotic activity for some bilothiazoles. This is supported by biosensor-experiments, where bilothiazoles C and E show P_*recA*_-suppressing activity, while bilothiazole F induces P_*blaZ*_, a biosensor characteristic for β-lactam antibiotics. These findings serve as a starting point for investigating the role of bilothiazoles in the pathogenicity of *Bilophila* species in the gut.

## Introduction

The human gut hosts a large variety of microbial species, among them thousands of different bacterial species, which together form a complex ecosystem.^1,2^ A healthy gut microbiota is generally characterized by stable coexistence of symbiotic bacterial species and provides significant benefits for the host, such as colonization resistance against pathogenic bacteria, immunomodulation, and nutrient uptake.^3,4^ Alterations in bacterial composition, however, are linked to development of chronic diseases, including inflammatory bowel disease and colorectal cancer.

Microbial balance can be disrupted by a range of exogenous and endogenous factors, most notably antibiotic treatment, which can, e.g., lead to subsequent infections with *Clostridium difficile* or *Klebsiella oxytoca*.^5,6^ Host-produced antimicrobial peptides are recognized as an important factor to balance the intestinal microbiota. Such compounds can be produced by cells of the gastrointestinal tract and are a key component of the mammalian immune systems.^7,8^ In addition to such host-derived compounds, members of the gut microbiota possess their own, often strain-specific specialized metabolism, which can provide competitive advantages in this densely populated environment.^9,10^ Bacterial antibiotics are indeed a key component to understanding antagonistic microbe–microbe interactions in gut environments.^11,12^ These include a range of ribosomally synthesized and post-translationally modified peptides (RiPPs) with antibacterial activities.^13,14^ The importance to foster our understanding of the impact of such microbial natural products (NPs) is underlined by bioinformatic studies predicting the genetic capacity of the human gut microbiota to produce thousands of yet unknown NPs with unknown functions.^15,16^

**Figure 1:**
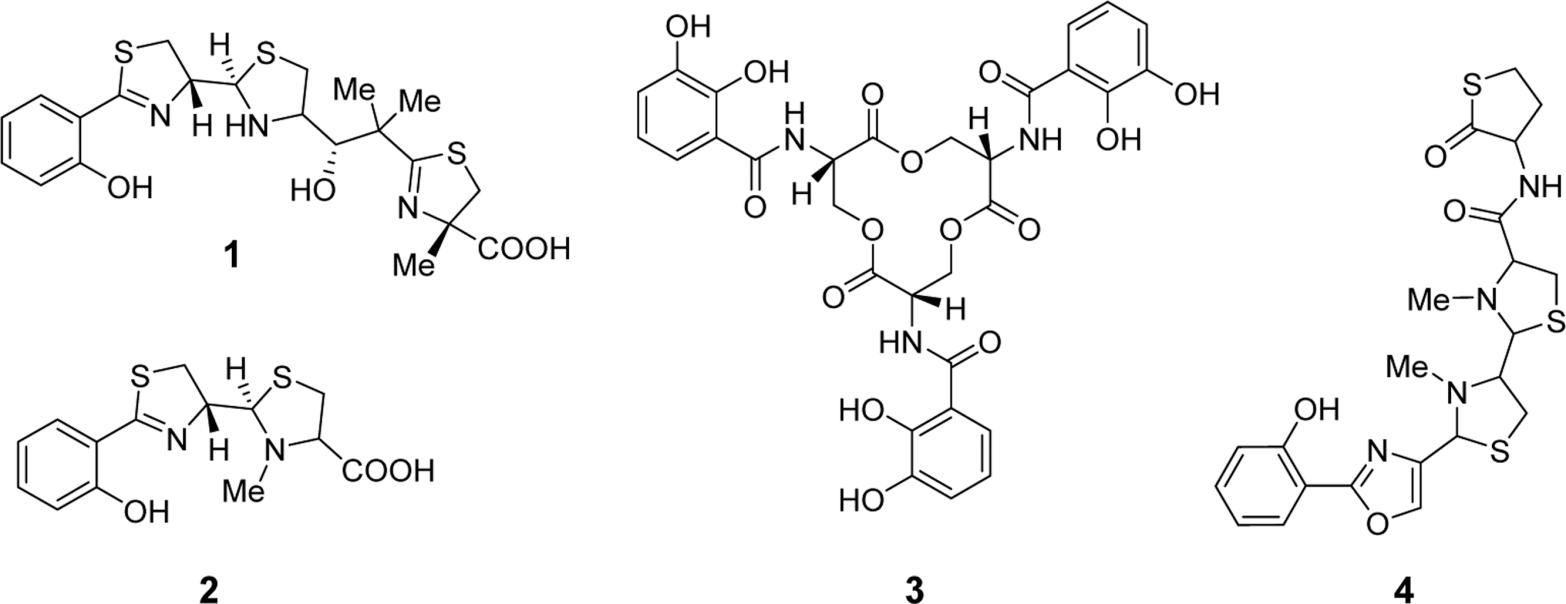
Structures of iron-chelating molecules yersiniabactin (**1**), pyochelin (**2**), enterobactin (**3**) and anthrochelin *D*/sorangibactin A (**4**). **1**, **2** and **4** share the arylthiazole/-oxazole structural motif.

Apart from antibiotic activity, NPs can, e.g., act as siderophores, which can also influence microbe–microbe interactions in the gut. Well-characterized siderophores, such as yersiniabactin (ybt, **1**), pyochelin (pch, **2**), or enterobactin (**3**) (Figure 1), facilitate bacterial iron uptake in competitive environments and play a significant role in the virulence of pathogenic bacteria such as *Klebsiella pneumoniae*, *Escherichia coli*, and *Pseudomonas aeruginosa*.^17−19^ Compounds **1**–**3** are nonribosomal peptides (NRPs), which is a family of NPs renowned for their broad range of bioactivities. NRPs are assembled from amino acid precursors by NRP synthethases (NRPSs), which are encoded by large genes organized in so-called biosynthetic gene clusters (BGCs) that can be readily identified by bioinformatic analysis of bacterial genomes using rule-based methods with tools such as antiSMASH.^20^ Compounds **1** and **2** belong to the growing family of arylthiazole siderophores. Recently discovered members include the anthrochelins, for example antrochelin *D* (**4**)^21^ from a human pathogen, and the myxobacterial sorangibactins.^22^

Virulence factors such as **1** also play important roles in gut environments. For example, it was shown that *E. coli* strains containing the *ybt* BGC promote inflammation-associated fibrosis in mice.^23^ This suggests that such compounds from the gut microbiota are not only relevant for microbe–microbe- but also key to pathogenic microbe-host interactions. Our group is interested in accessing such yet undiscovered NPs from the gut microbiome with potential effects on microbiome composition and host health, but also with application potential in biomedicine. In this study, we bioinformatically analyzed a gut metagenomic data set and identified a functionally uncharacterized BGC from uncultured *Bilophila* sp. Putatively encoding an arylthiazole NP. The BGC was made available by gene synthesis and heterologously expressed in *E. coli*, leading to the isolation and characterization of new arylthiazole analogs, the bilothiazoles A–E (**5**–**10**), with antibiotic activity.

## Results and Discussion

### Bioinformatic Analysis

**Figure 2:**
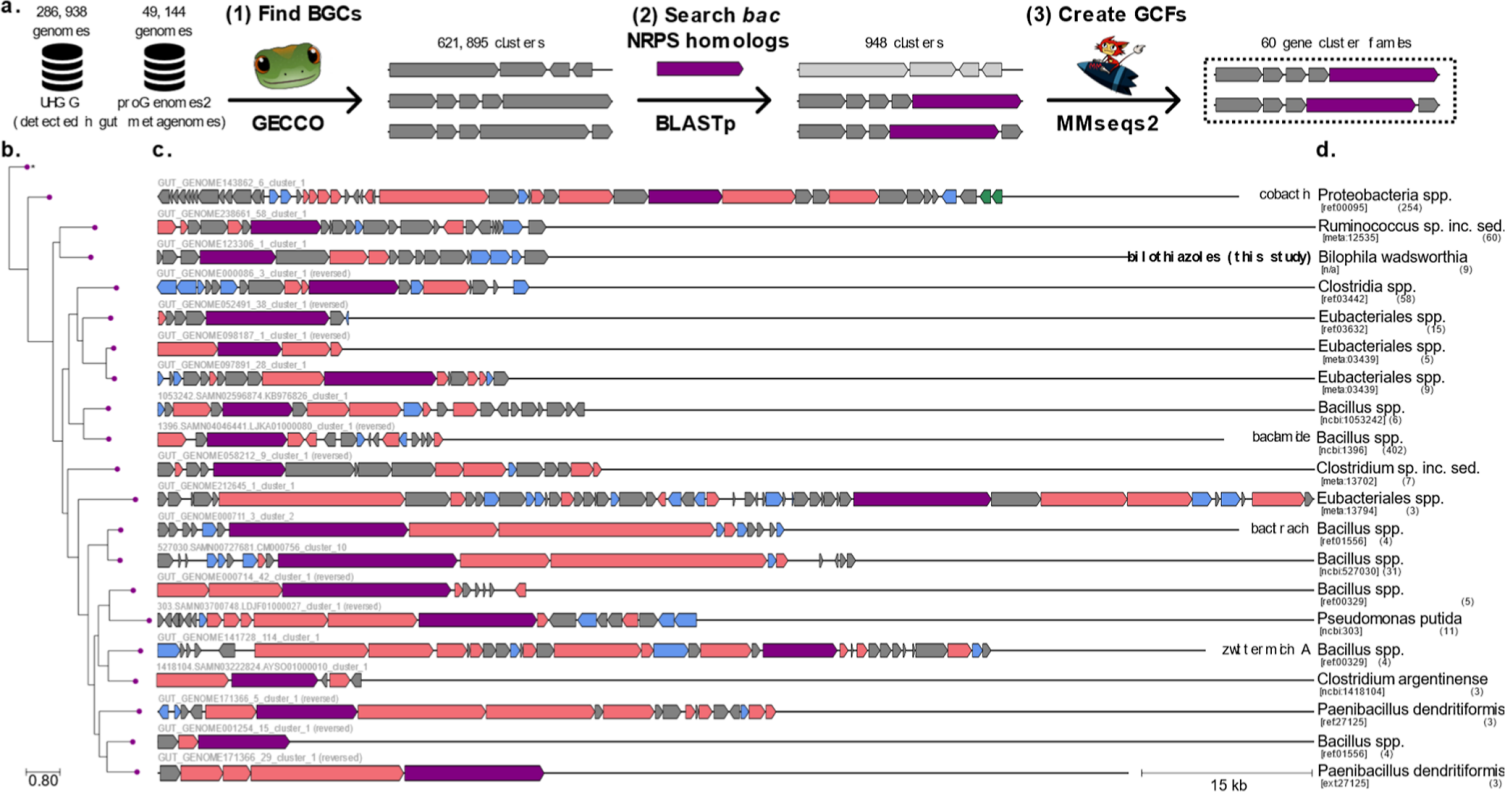
(a) Bioinformatics workflow for identifying thiazole-producing BGCs. (b) Phylogenetic tree of the NRPS proteins in the cluster representatives of the 20 most populated GCFs. The protein sequences of *bac* NRPS homologues were aligned using MUSCLE (v5.1 with default parameters).^31^ The multiple sequence alignment was passed to FastTree2 (v2.1.11 with default parameters)^32^ to build an approximately maximum-likelihood phylogenetic tree. The tree is displayed with the ETE Toolkit (v3.1.3).^33^ (c) The corresponding representative BGC for each GCF. The different cluster sequences were rendered using the dna-features-viewer package (v3.1.3).^34^ Genes are colored according to the GECCO function prediction based on the Pfam domain content of each gene, either transporter (blue), biosynthetic (pink), regulatory (green) or unknown (gray); the *bac* NRPS homologue is shown in purple. Known BGCs that could be identified in literature have the produced compounds written on the right-hand side. (d) The taxonomy and taxonomic identifiers of each cluster representative are shown in square brackets according to either the NCBI Taxonomy^35^ for isolate genomes (ncbi), or to the mOTUs 3.1 taxonomy^36^ for MAGs (ext, ref or meta). The number of BGCs in each GCF is shown in brackets.

In previous studies, we identified the thiazol(in)e structural motif as a promising predictor of biologically active NPs in the gut.^24^ Therefore, we explored two metagenomic data sets [Unified Human Gut Genome collection (UHGG)^25^ and a gut-associated subset of proGenomes2^26^ for BGCs encoding NRPSs incorporating modules typically responsible for heterocyclization enzymology similar to the *bac* BGC encoding the bacillamides. We then ordered the BGCs into Gene Cluster Families (GCFs) based on sequence similarity of their gene content (specifics see page 15, Figure 2a,b).^27^ This led to the identification of the *bil* BGC, which was found to originate from an uncultured *Bilophila* sp. strain (Figure 2c,d). The related species *Bilophila wadsworthia* is a known pathobiont in the gut and was first isolated from infected appendices.^28,29^ More recently, it was shown to produce hydrogen sulfide in the gut, which is linked to the development of inflammatory bowel disease and colorectal cancer.^30^ As there is only very limited knowledge of NPs from *Desulfovibrionia*, this makes the products of the *bil* BGC a promising starting point for NP discovery and for further evaluating the virulence and pathogenicity of this strain.

**Figure 3:**
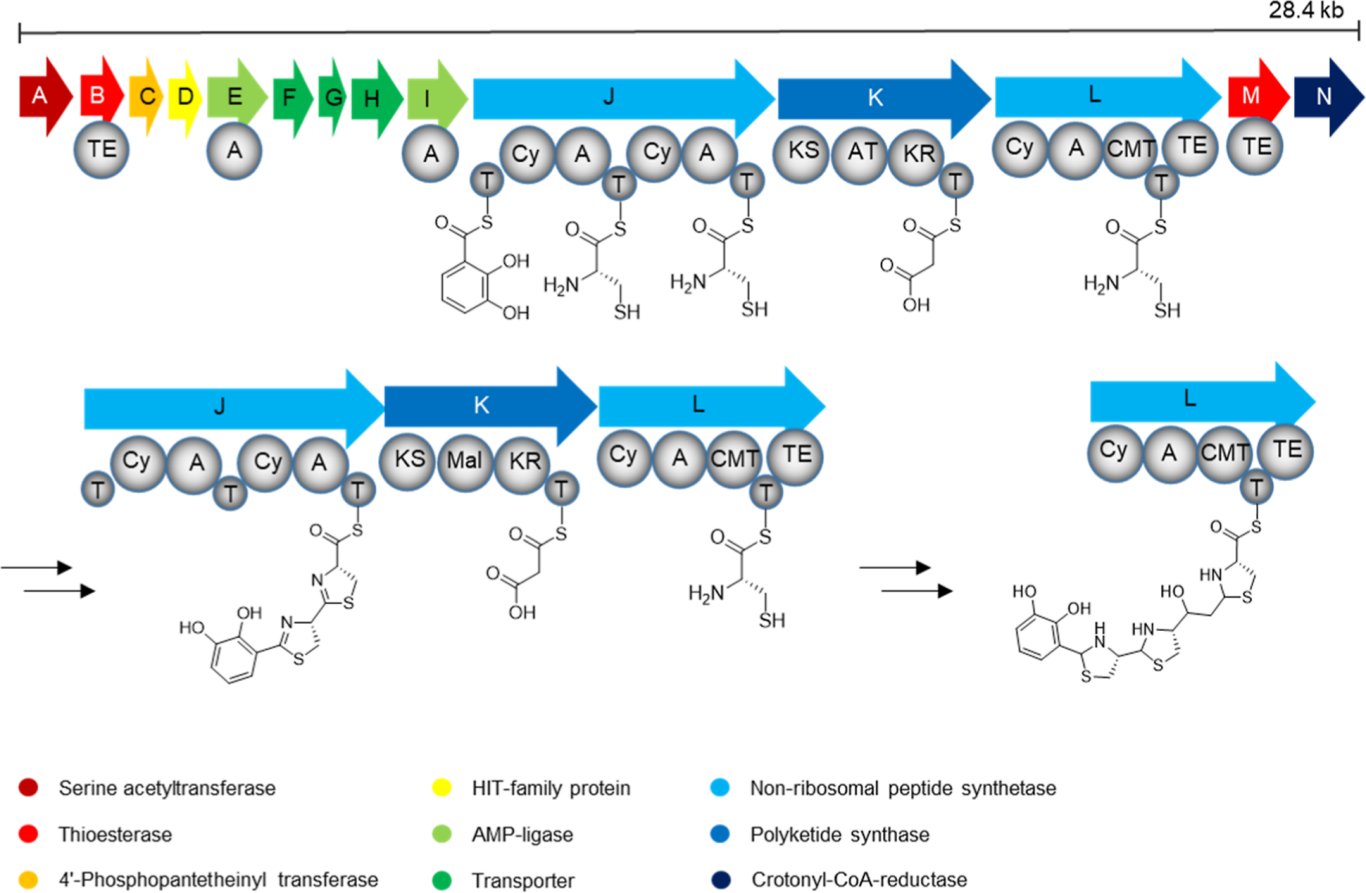
Architecture of the cryptic *bil* gene locus with predicted domain-structure of the NRPS/PKS-system and potential intermediate products of PKS/NRPS-assembly. Instead of 2,3-DHB, also 2,3-DAP could be loaded onto the first T-domain. All other A-domains are predicted to activate cysteine. A: A domain, AT: acyltransferase domain, Cy: cyclization domain, CMT: *C*-methyltransferase domain, KS: ketosynthase domain, KR: ketoreductase domain, TE: thioesterase domain, T: thiolation domain.

The *bil* BGC is composed of 14 genes *bilA*–*N*, including the three biosynthetic core genes *bilJ-L* encoding polyketide (PKS) and NRPS machinery with a total of 4 biosynthetic modules (Figure 3, Table S3). The AMP-ligase encoded by *bilI* shows a high degree of similarity to the 2,3-dihydroxybenzoate (DHB)-loading DhbE from *Bacillus subtilis* and the salicylate-loading PchD from the pyochelin (*pch*) pathway. The NRPS BilJ is similar to Irp2 from the yersiniabactin (*ybt*) BGC (33.0% identity) and to PchF (31.4% identity). The thioesterase encoded by *bilB* also shows similarities to these two pathways, but the *bil* BGC lacks the *N*-methyl transferase of the *pch* BGC and generally has a different architecture compared to *ybt*, therefore suggesting it to produce novel NP structures (see Figure S1). The gene *bilM* is predicted to encode another type II thioesterase and the AMP-ligase encoded by *bilE* is predicted to load 2,3-diaminopropionate (DAP), potentially offering another starting material. Core biosynthetic genes *bilJ* and *bilK* were not homologous to any characterized genes and the domain-structure of their encoded proteins, as assessed by Prism,^37^ is depicted in Figure 3. In short, biosynthesis is expected to start with loading of either 2,3-DAP or 2,3-DHB onto the first T-Domain, followed by stepwise fusion with two cysteine moieties, which both should undergo heterocyclization catalyzed by the cyclization domains (Cy). This intermediate would then be further extended with a malonyl-CoA building block by PKS BilK and another, potentially C-methylated thiazol(in)e heterocycle by BilL.

### Cloning of the *bil* BGC

**Figure 4:**
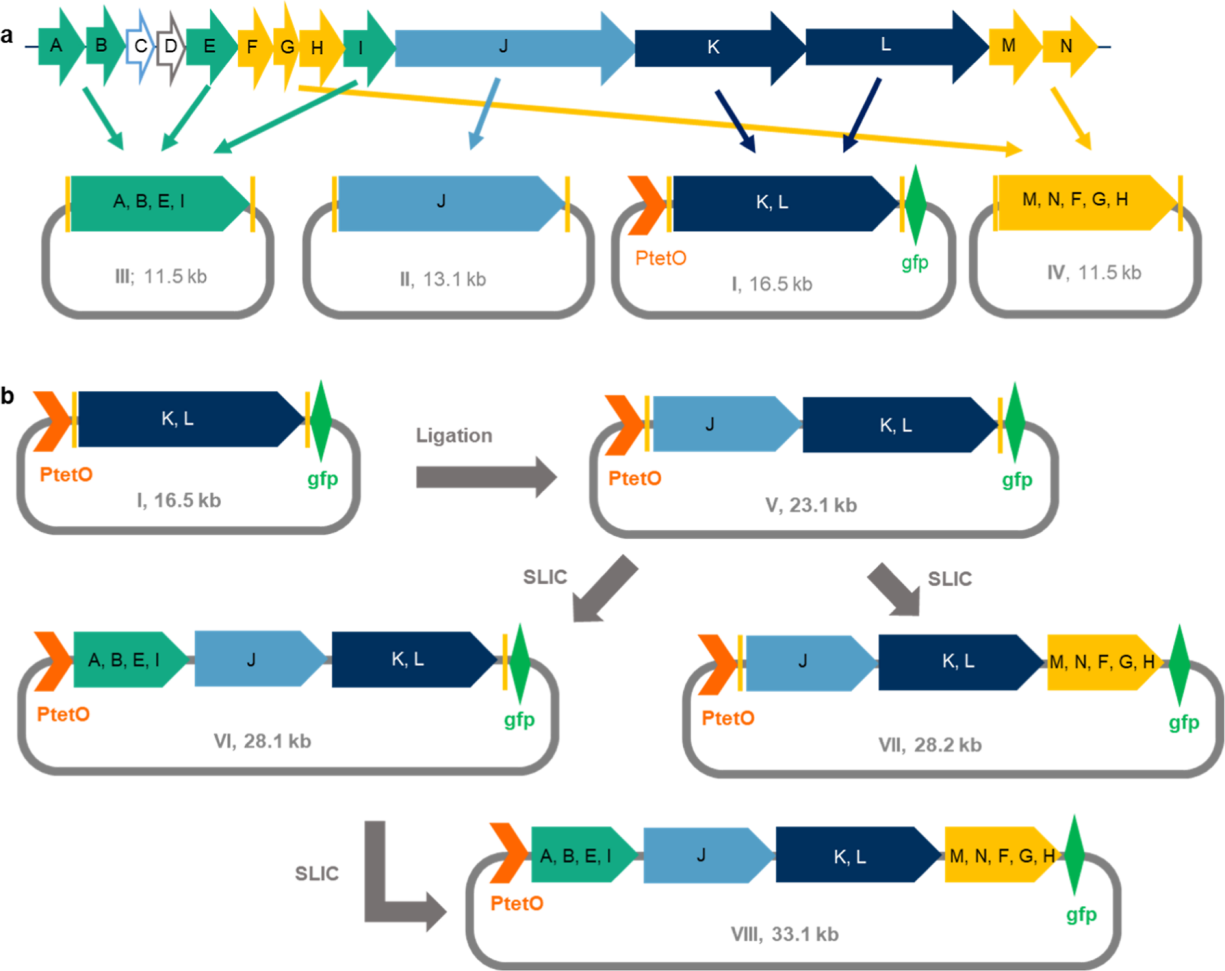
Cloning strategy for the *bil* BGC. (a) Reorganization of the biosynthetic genes into four synthetic plasmids (I, dark blue, *bilKL*; II, light blue, *bilJ*; III, green, *bilABEI*; IV, yellow, *bilMNFGH*). (b) Schematic representation of cloning strategy for stepwise assembly of the expression vectors V–VIII by Ligation and SLIC.

The cloning of BGCs identified from metagenomic data is often not possible due to the lack of information on the identity of the original BGC host strains and/or the lack of access to these strains or their gDNA. A solution to this problem is the *de-novo* synthesis of the respective genetic sequence and its subsequent introduction into a suitable expression vector for recombinant production. Construction of the expression vector can readily be achieved using Direct Pathway Cloning.^24,38−42^ Following this approach, we selected 12 of the 14 genes of the *bil* BGC to be included in the final expression construct. Gene *bilC*, encoding for a putative phosphopantetheinyl transferase (PPTase), was omitted as the foreseen heterolougous expression strain *E. coli* BAP1 harbors the promiscuous PPTase Sfp.^43^ Gene *bilD* encoding a HIT-family protein was not thought to have a biosynthetic function and was thus likewise not included. Due to general size limitations in commercial gene synthesis, all other target genes were redistributed over four synthetic gene fragments, including the PKS-encoding genes *bilKL* (fragment I, dark blue, size: 9956 bp), the NRPS-encoding gene *bilJ* (fragment II, light-blue, size: 6690 bp), genes *bilABEI* (fragment III, green, size: 4959 bp), and genes *bilMNFGH* (fragment IV, yellow, size: 4966 bp) (Figure 4a). All genes were codon-optimized for expression in *E. coli*. Genes *bilKL* were directly integrated into the vector backbone pET28b-ptetO::*gfp* to yield vector construct I. Starting from I, the cluster was reassembled into expression vectors V, VI, VII and VIII in a stepwise manner, as depicted in Figure 4b.

Assembly of vector construct V involved linearization of I by restriction digest with *Eco*RI and ligation with the excised gene *bilJ* from fragment II, digested with the same restriction enzyme. Expression vectors VI and VII were assembled from V utilizing SLIC.^44^ Briefly, V was linearized with restriction digest and the insert fragment was amplified by PCR from its synthetic vector, attaching 23–25 bp homology arms, followed by SLIC-assembly to yield the circular expression plasmid. The complete plasmid VIII was assembled from VI in an analogous fashion. Pictures of the SDS-gel analyses for cloning, colony screening, and restriction digests are provided in the Supporting Information (Figures S2–S5). Plasmids V and VI were also validated by sequencing (Figures S6 and S7). This method of stepwise construct assembly furthermore enabled comparative metabolomics between the differentially equipped expression constructs.

### Heterologous Expression and NP Isolation

**Figure 5:**
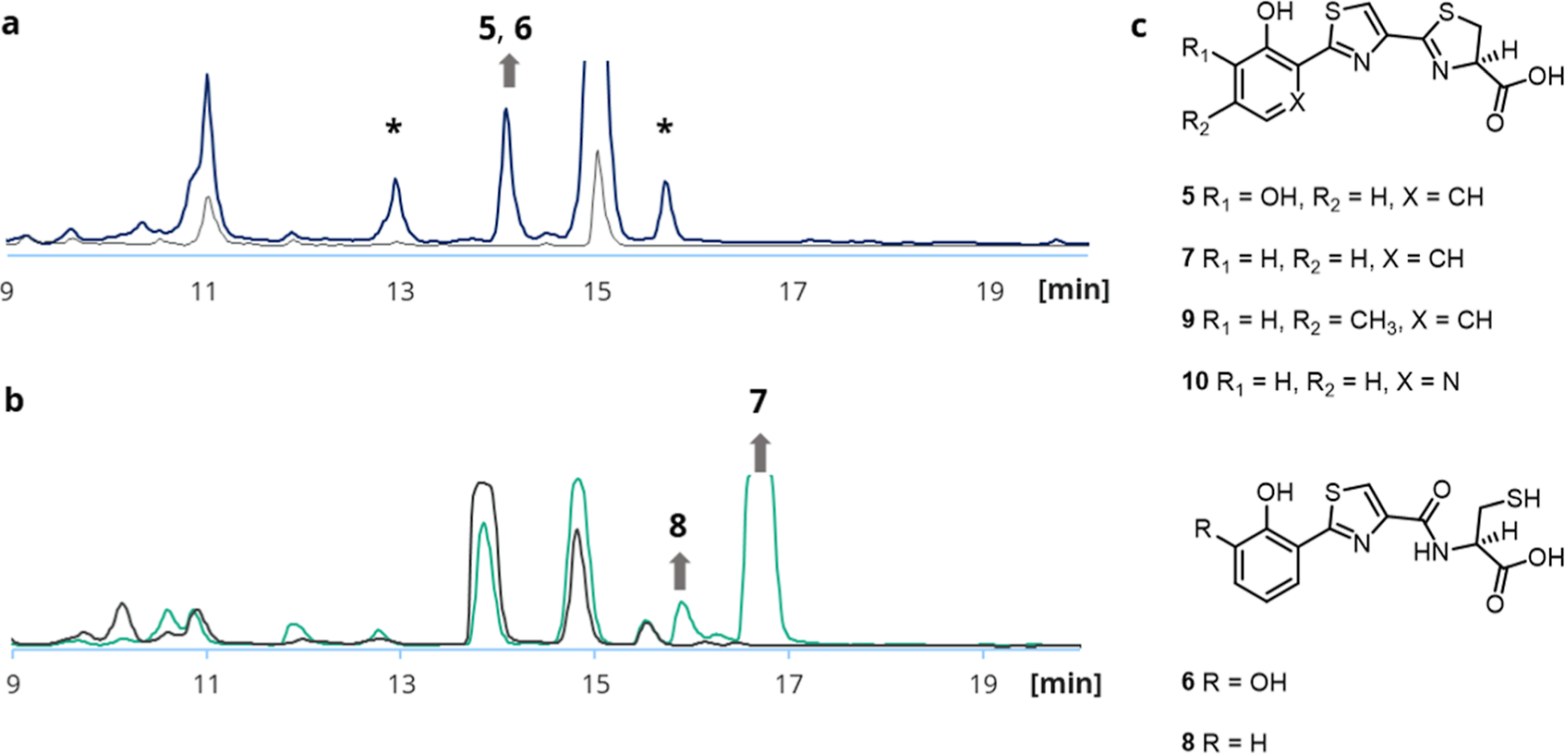
(a) Identification of **5** and **6** in extracts of culture supernatants of cultures of *E. coli* BAP1 after 50 h with construct **VI** in M9 medium (blue), compared to negative control (gray). (*) These compounds had molecular masses that were also present in the control expression, albeit at significantly lower abundance. (b) Identification of **7** and **8** in extracts of salicylic-acid-supplemented cultures (blue-green), compared to negative control (gray). (c) Structures of bilothiazoles A and B (**5** and **6**), C (**7**), D (**8**), E (**9**) and F (**10**).

The expression vectors V–VIII were amplified by transformation and cultivating *E. coli* DH5α with subsequent plasmid isolation, followed by transformation into the recombinant host of choice, *E. coli* BAP1. Heterologous expression was tested in TB, LB, and M9-media (100 mL each) for durations of 64 and 122 h after induction with tetracycline. Cells were separated from spent media by centrifugation and both samples were extracted with ethyl acetate. Initial heterologous expression experiments using vectors VI and VIII in M9 medium led to the production of a new compound eluting at 14 min during HPLC analysis, which was not present in control expressions using the empty vector pET28b-ptetO-*gfpV2* (Figure 5a). As the production titer of this molecule using construct VI was slightly higher, all further expressions were carried out with this plasmid. In expressions with the constructs not containing insert III (V and VII), the compound eluting at 14 min was not observed.

After initial isolation from small-scale cultures (100 mL M9), it was found that the HPLC-UV signal detected at 14 min corresponded to a mixture of two coeluting compounds with molecular masses at *m*/*z* 323.0153 [M + H]^+^ (**5**) and *m*/*z* 341.0260 (**6**). However, the production yield at this cultivation scale turned out to be too low for compound characterization by NMR. The *m*/*z*-values corresponded to calculated chemical formulas of C_13_H_10_N_2_O_4_S_2_ ([M + H]^+^ = 323.0155) for **5** and C_13_H_12_N_2_O_5_S_2_ ([M + H]^+^ = 341.0260) for **6**. Given the structural predictions for the *bil* assembly line (Figure 3), this suggested a potential offloading of the NRPS product **5** by thiolysis from the final T domain of NPRS BilJ, with subsequent partial hydrolytic opening of the thiazoline ring to give **6** (Figure 5c). For both molecules, the incorporation of a 2,3-dihydroxybenzoic acid (DHBA) starter unit was thus assumed. To increase production of **5** and **6**, expression cultures were supplemented with 125 μM 2,3-DHBA, which indeed greatly enhanced the production titer (from approximately 0.1 to 3.8 mg/L), thereby enabling compound isolation and NMR structure analysis in DMSO-*d*_6_.

**Figure 6:**
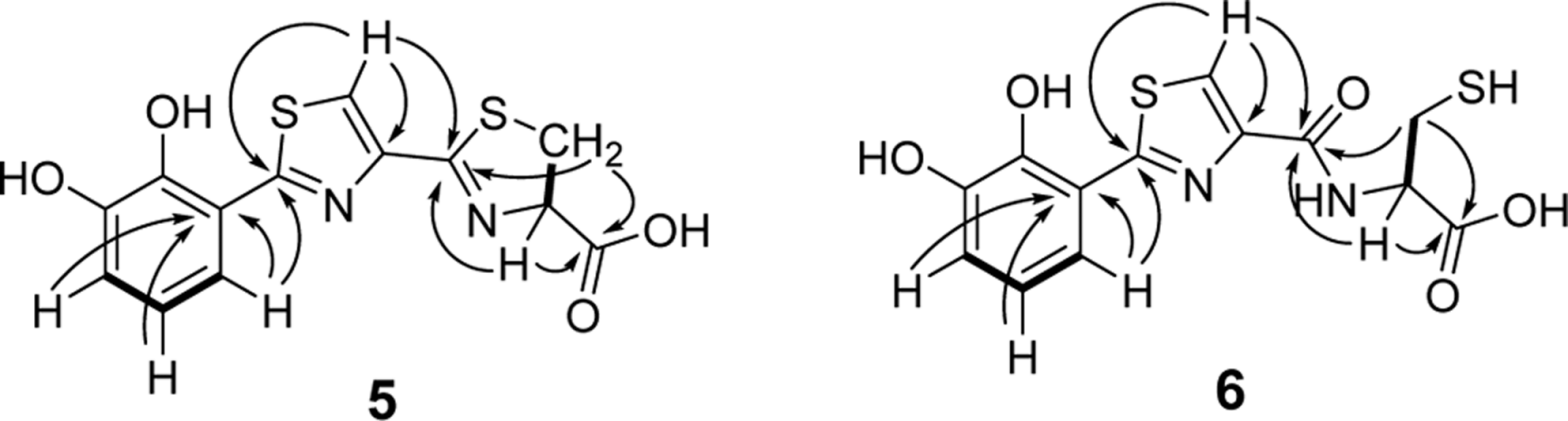
Chemical structures of bilothiazoles A (**5**) and B (**6**) with key COSY (bold) and HMBC (arrows) correlations.

The proposed chemical formulas for the identified masses indicated 10 and 9 double bond equivalents for **5** and **6**, respectively. The ^1^H NMR spectrum conferred the presence of several aromatic hydrogens, six of which could be designated to two coexisting DHB-moieties by COSY. The signal sets for **5** and **6** were very similar, except for the chemical shifts of the thiazoline/cysteine, which are located at δ(^1^H) = 5.31 and 3.62 ppm for **5** and 4.65 and 3.05 ppm for **6** (see Figure S26). The cysteine-moiety of **6** is marked by the presence of an adjacent nitrogen-bound hydrogen, as determined by 2D-NMR (^1^H–COSY, ^1^H, ^13^C-HSQC). Further ^1^H, ^13^C-HMBC-analysis confirmed the existence of a thiazole in each molecule, connecting the aforementioned structural elements (Figure 6). In conclusion, these analyses confirmed the initially proposed structures of the isolated NPs, which can thus be classified as shunt products of the *bil*-biosynthetic pathway, resulting from premature hydrolytic offloading from BilJ. The originally anticipated end product of the *bil* BGC remained absent in our heterologous expression experiments. Nonetheless the discovery of **5** and **6** is of interest, as to our knowledge, these structures have not been reported before.

### Mutasynthetic Studies for NP Diversification

Given the greatly enhanced production titers when feeding the 2,3-DHBA NRPS starter unit, a mutasynthetic approach was tested to evaluate opportunities for compound structural diversification. A total of 18 additional benzoic acid derivatives was thus screened as culture supplements. Ten of these did not result in any product formation (see Table S4 for details). For benzoic acid, 3-hydroxy benzoic acid, 4-amino salicylic acid, vanillic acid and ortho-vanilic acid, small amounts of the expected NP analogs were detectable by HR LC–MS (Figures S8–12), unfortunately at yields too low for compound isolation. Supplementation with salicylic acid (products **7** and **8**, 33.3 and 3.9 mg/L, Figure 5b), 4-methyl salicylic acid (**9**, 9.8 mg/L), or 3-hydroxy picolinic acid (**10**, 1.9 mg/L) led to the formation of sufficient quantities of products for isolation and structural characterization (Figure 5c). Bilothiazole C (**7**) was previously reported in literature as “HPTT-COOH” and its ^1^H and ^13^C NMR spectra were in agreement to the reported data.^45^ Bilothiazole D (**8**) was easily distinguishable from **7** by the presence of a nitrogen-bound hydrogen and the different chemical shifts of the cysteine not involved in heterocyclization (δ(^1^H) = 4.86 and 3.15 ppm, spectrum in Figure S33). HPTT-COOH (**7**) and HPT-Cys (**8**) were previously identified as intermediates in pyochelin biosynthesis but no bioactivities had been determined.^46,47^ Compound **7** has also been found to be a byproduct of yersiniabactin-biosynthesis, detected in urinary tract infections, and possibly playing a protective role against *Pseudomonas*.^48^

Bilothiazole E (**9**) featured the expected methyl-group attached to the aromatic system (δ(^1^H) = 2.29 ppm, δ(^13^C) = 21.1 ppm, Figure S39) and the NMR spectroscopic data was otherwise in line with those of **7**. For **10**, the aromatic system matched the expected chemical shifts for 3-hydroxy-picolinic acid (δ(^1^H) = 8.25, 7.56, 7.49), with again comparable NMR data with respect to **7** (Figure S45). Overall, this work thus proved some degree of starter unit promiscuity by the loading A domain of the *bil* BGC, facilitating incorporation of four out of a total of 19 tested starter units.

The BGC encodes two alternative free-standing A-domains, BilE and BilI, which could perform *in-trans* starter-unit selection and activation for NRPS-assembly (Figure 3). Bioinformatic sequence analysis predicts BilE is to load 2,3-diaminopropionate, while BilI is predicted to activate salicylate and, to a lesser extent, 2,3-DHB. Evaluation of the heterologous expression experiments leads to the conclusion that BilE is not involved in precursor recruitment or inactive in the recombinant production system (no products with 2,3-diaminopropionate starter unit), while BilI is indeed capable of accepting salicylic acid, 2,3-DHB, and the above-mentioned structural analogs. While molecule **5** incorporating 2,3-DHB was the main product of unsupplemented expressions of the *bil* BGC, we also found evidence for formation of **7** incorporating salicylic acid in LC–MS data of raw extracts of unsupplemented expression cultures, thus suggesting both molecules to be NPs. The lower abundance of **7** (about 1% of **5**, see Figure S13) might rather be a result of limited starter unit availability in *E. coli*, as the alternative building block 2,3-DHB is also produced in the biosynthesis of the siderophore enterobactin.^49,50^ However, salicylate seems to actually be the preferred substrate of the BGC, since it showed the highest production titer of all tested substrates within our mutasynthetic experiments (Table S4).

It is interesting to note that the formation of the open-chain derivatives **6** and **8** stem from hydrolysis of the thiazoline ring in **5** and **7** after their biosynthetic assembly. This was suspected to be catalyzed by acidic conditions, e. g., during extraction from the culture broth or HPLC/MPLC-purification. In raw extracts of expressions supplemented with 2,3-DHBA, the ratio of **5** to **6** was roughly 13:1, as assessed by MS (Figure S14). After purification, the ratio changed to 4:5, as determined by ^1^H NMR (based on the characteristic peaks of the terminal cysteine for **6** at δ 4.65 and 3.05 ppm, Figure S26). This occurred despite choosing a non-TFA-supplemented mobile phase during chromatographic purification, indicating a lability of the molecule already under neutral conditions. In contrast to the mixture of **5** and **6**, compound **7** had a longer retention time compared to its open-chain derivative **8** and therefore their separation was readily achieved by prep-HPLC. Interestingly, both, compounds **9** and **10**, seemed far less prone to ring-opening and only traces of their ring-opened forms were detected.

### Bioactivity

As the thiazole structural moiety had previously been linked to DNA-binding activity and thiazole-containing molecules in the gut are known as genotoxins^51,52^ and cytotoxins,^24^ we initially tested the activity of NPs **7** and **8** against human colorectal cancer HCT116 cells. These substances were selected due to their higher availability and easier purification, compared to the others, and to determine whether the open or closed state of the thiazoline-ring had an effect on activity. While it was confirmed that **7** is readily taken up into the cytosol (Figure S15), inhibitory activity was only observed at concentrations > 250 μM in clonogenic survival assays (Figure S16). In contrast, almost no uptake of **8** into the cells was observed, which consequently showed no cytotoxic activity. For more accurate quantification, the activities of bilothiazoles C–E (**7**–**9**) were analyzed in SRB-assays, which again turned out to be weak (Figure S17).

**Figure 7:**
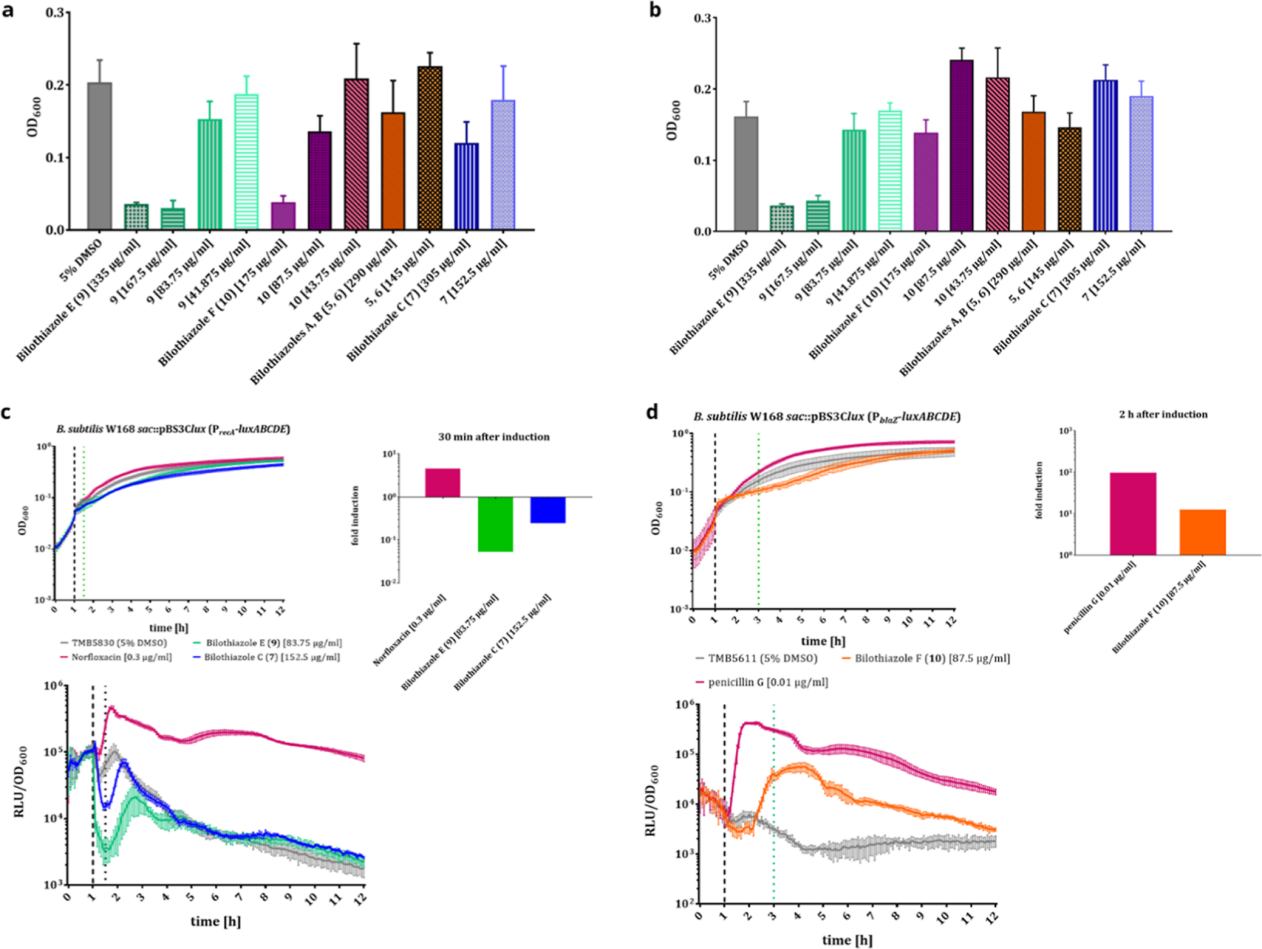
Antibiotic activity of the bilothiazoles: (A) OD_600_ measurements 3 h after treatment: **9** and **10** show the strongest activity against *B. subtilis* W168. (B) OD_600_ measurements 3 h after induction: only **9** is active against *S. aureus*. (C) Repression of the P_*recA*_-promoter by **7** and **9** compared to positive control (norfloxacin). (D) Induction of P_*blaZ*_-promoter by **10**, compared to positive (penicillin G) and negative control.

Next, we turned to the evaluation of antibacterial activity. In overlay-assays, inhibitory activity against a panel of Gram-positive and -negative bacteria was tested. This indicated weak inhibition of *B. subtilis* and *Staphylococcus aureus* upon treatment with **7** and, to a lesser extent, **8** (Figure S18). Given the presence of the *bil* BGC in the human gut metagenome, this encouraged further tests of all molecules produced by the BGC, including mutasynthetic analogs, against these bacterial pathogens. While **5**, **6** and **8** were generally less effective against bacterial strains, mutasynthetic derivatives **9** and **10** showed the most potent, yet still rather weak effects at an inhibition of up to 83.8 μg/mL for **9** against *B. subtilis* (Figure S19). Compound **9** was the only bilothiazole inhibiting the growth of *S. aureus*. When tested against *Penicillium chrysogenum*, none of the NPs displayed antifungal properties (see Table S5, Figure 7a,b).

In a follow-up experiment, we aimed to get further insights into the potential molecular targets of the antibacterial bilothiazoles. Therefore, we employed *B. subtilis* whole-cell biosensor strains, containing a promoter (P_*recA*_, P_*blaZ*_, P_*liaI*_, P_*bceA*_, P_*psdA*_, P_*yrzI*_, P_*helD*_, Py_*fiLMN*_) fused to a luciferase cassette. The induction and activity of the used promoters results in bioluminescense that can be quantified by plate-reader assays. For the bilothiazoles, we identified two separate potential functions: bilothiazoles C (**7**) and E (**9**) were found to strongly repress the P_*recA*_-promoter (>10-fold) in *B. subtilis* upon treatment (Figures 7c and S20), which indicates their involvement in the repression of DNA-repair. Recombinases from the RecA-family are found in virtually all bacteria and are regulated within the SOS-stress-response.^53,54^ However, the mechanism underlying the RecA-repression of **7** and **9** remains unknown.

Interestingly, bilothiazole F (**10**) was instead found to induce the P_*blaZ*_-biosensor, which is characteristic for β-lactam antibiotics (Figures 7d and S21). BlaZ is a β-lactamase, conferring resistance to these antibiotics by hydrolysis of the β-lactam ring.^55,56^ While **10** does not possess this structural moiety, these results might indicate the compound to also inhibit transpeptidases and therefore induce β-lactamase activity.

## Conclusion

In conclusion, we report the discovery of the *bil*-BGC from a metagenomic data set, its assembly from synthetic DNA into several different expression vectors, and its heterologous expression in *E. coli* leading to the discovery of the new NPs bilothiazoles A (**5**) and B (**6**). Furthermore, mutasynthetic experiments revealed some degree of substrate promiscuity concerning the NRPS starter unit, which allowed for the production of bilothiazole derivatives **7**–**10**. Among those, **7** and **8** were previously known as intermediate products of pyochelin biosynthesis, and **7** is known to possess Fe(III)-binding activity.^48^ Preliminary tests in our hands (lack of detection of metal adducts in MS analysis; no detectable Fe(III)-solubilization; no effect of potential Fe(III)-binding on cytotoxic activity) did, however, not indicate significant metal-binding properties. Since the biosynthesis of the bilothiazoles can be entirely explained without *bilK* and *bilL*, the observed compounds are shunt products of the *bil* BGC. However, in our heterologous expressions under varied expression conditions, we did not find evidence for a larger final product of the *bil*-BGC. This indicates that *bilKL* might be inactive under our culture conditions or that the final product is unstable outside the cells. This phenomenon is known from other BGCs putatively encoding thiazol(in)e-containing products, such as the enigmatic coelibactin from *Streptomyces coelicolor* A3(2).^57^

Furthermore, in-depth biological activity testing revealed that the bilothiazoles show weak antibacterial activity. While **7** and **9** were found to suppress the P_*recA*_-promoter, indicating inhibition of DNA-repair, **10** surprisingly induced P_*blaZ*_, which might indicate inhibitory effects on transpeptidases. These findings shed light on the metabolism of *Bilophila* species, bacterial strains that possibly have detrimental effects on gut health. Members of the *Bilophila* genus have been linked to appendiceal infections and hydrogen sulfide production in the intestine, which has effects on disease pathology. Further studies on the bilothiazoles are currently underway in our group to better understand their potential role in human intestinal health.

## Materials and Methods

### Strains, Plasmids, Cell Lines, Enzymes

Bacterial strains and plasmids used in this study are listed in Table S1. *E. coli* strains were cultivated at 37 °C in LB medium supplemented with a suitable selection antibiotic while shaking at 180 rpm, or on LB-Agar supplemented with selection antibiotic at 37 °C unless otherwise specified. DNA was kept in Milli-Q water for short-term storage. For long-term storage, plasmids were transformed into *E. coli* DH5α and cryostocks (75% LB-medium, 25% glycerol) were stored at −80 °C. *B. subtilis* and *S. aureus* were routinely grown in Lysogeny broth (LB-Medium (Luria/Miller), Carl Roth GmbH & Co., KG, Karlsruhe, Germany) at 37 °C with agitation. 1.5% (w/v) agar (Agar–Agar Kobe I, Carl Roth GmbH & Co., KG, Karlsruhe, Germany) was added to prepare the corresponding solid media. Due to their BSL-2 status, all experiments involving BSL-2-microorganisms were conducted in a BSL-2 laboratory (Institute for Microbiology, TU Dresden, Dresden, Germany).

All restriction enzymes for this study were purchased from NEB. Culture supplements were generally dissolved to 1 or 0.5 M stock solutions in DMSO.

### Bioinformatics

We applied GECCO (v0.9.2)^58^ to a set of 49,144 isolate genomes of bacterial species and 286,938 metagenome-assembled genomes (MAGs) originating from the human gut.^25,26^ GECCO predicted 621,895 candidate biosynthetic gene clusters (BGCs). We screened the GECCO predictions to find BGCs containing homologues to the *bac* NRPS,^24^ using *blastp* (NCBI BLAST + v2.5.0 with default parameters),^59^ yielding 948 clusters with at least one hit. We then clustered the selected clusters into Gene Cluster Families (GCFs) using MMseqs2 linclust pipeline (MMseqs2 v13.45111 with --cov-mode 1 --cluster-mode 1 -c 0.7 min-seq-id 0.5),^60^ yielding 60 GCFs including 29 singletons. Further analysis of BGCs was performed with AntiSMASH (Version 6)^20^ and PRISM (Version 3).^37^ All sequences and plasmids were analyzed, edited and saved in the Geneious software package (Version 8.1.9).^61^ Gene cluster comparison was performed with Clinker (https://cagecat.bioinformatics.nl/)^62^ using standard input parameters.

### Cloning

Constructs for this study were prepared either by ligation cloning (pET28b-ptetO::7246p1+2_*gfp*) or SLIC (all other constructs). For the ligation step, linearized fragments of insert and backbone were prepared by preparative restriction digest with *Eco*RI and purified by gel extraction. The linearized backbone fragment was dephosphorylated with Antarctic phosphatase (NEB) prior to purification to prevent religation. The ligation reaction was performed with T4 DNA ligase (NEB) using 0.02 pmol of linearized backbone and 0.06 pmol insert. For SLIC-cloning, linear fragments of the insets were generated by PCR using primers listed in Table S2 and plasmids listed in Table S1. In general, homology arms of 20–25 base pairs were used and placed on the inset fragment by PCR with Q5-Polymerase (NEB) in 50 μL batches consisting of: 1 × Q5 reaction buffer, 200 μM dexynucleotide triphosphates, 500 nM of forward and reverse primer, 10 ng plasmid-template and 0.01 U/μL Q5 High-Fidelity DNA polymerase (NEB). Thermal cycling was performed in a T100 Thermal Cycler (Bio-Rad) as follows: (1) Initial denaturation, 98 °C for 30 s; (2) Denaturation, 98 °C for 10 s; (3) Primer annealing for 20 s; (4) Extension, 72 °C for 40 s/kb; (5) Final extension, 72 °C for 5 min. Steps (2 to 4) were repeated for 30 cycles in total. The annealing temperatures for specific primer pairs were estimated with the NEB Tm Calculator tool (https://tmcalculator.neb.com/). Vector backbones were linearized by restriction digest as described previously. SLIC-cloning was performed as described previously.^39^

Transformants were selected on LB-agar plates with kanamycin (kan) as selection antibiotic and initially screened by colony-PCR using Onetaq polymerase (NEB) for correctly assembled constructs. Clones were picked, resuspended in 12 μL of LB-medium supplemented with kan, and examined in a 25 μL PCR reaction composed as following: 1 × Onetaq Buffer, 200 μM deoxynucleotide triphosphates, 200 nM of forward and reverse primer, 2 μL cell suspension (DNA template) and *Onetaq* DNA polymerase (NEB). Thermocycling conditions were set as described above, with the exception of 94 °C denaturation and 68 °C extension temperatures. Positive clones were confirmed by restriction digest, terminal-end Sanger sequencing by Azenta and additionally full plasmid sequencing by SNPsaurus.

### Analytical and Preparative HPLC

Analytical high-performance liquid chromatography (HPLC) was performed on an Azura HPLC device manufactured by Knauer, consisting of the following components: AS 6.1L sampler, P 6.1L pump, DAD 2.1L detector. Components were separated on a Phenomenex Luna 3u C-18 column (150 × 4.6 mm) at a flow rate of 1 mL/min with the eluents water (A) and acetonitrile (B), both supplemented with 0.05% trifluoracetic acid. The elution method consisted of equilibration at 5% B for 2 min, followed by a gradient of 5–100% B over 28 min. Column washing was performed at 100% B for 5 min and the column was re-equilibrated at 5% B for 2 min before the next measurement.

Preparative HPLC was performed on a Jasco HPLC system consisting of an UV-1575 Intelligent UV/vis detector, two PU-2068 Intelligent preparation pumps, a Mika 1000 dynamic mixing chamber (1000 μL; Portmann Instruments AG Biel-Benken) and a LC-NetII/ADC anda Rheodyne injection valve. The system was controlled by Galaxie software. Chromatographic separation was performed on a Eurospher II 100-5 C18 A (250 × 16 mm) column with precolumn (30 × 16 mm) provided by Knauer at a flow-rate of 10 mL/min and the eluents were water (A) and acetonitrile (B). The gradient was adjusted depending on the polarity of the compounds. Collected product fractions were combined, the organic solvent was evaporated under reduced pressure at 40 °C and water was removed by lyophilization.

### MPLC Purification

Medium-pressure liquid chromatography (MPLC) was conducted on a Büchi Pure C-800 Flash MPLC system with a Reveleris 40 μm C18 cartridge (12 g) with the eluents water (A) and acetonitrile (B). Purification was generally achieved with a 20–40% B gradient over 20 min, followed by a 40–80% B gradient over 5 min. Collected product fractions were combined, the organic solvent was evaporated under reduced pressure at 40 °C and water was removed by lyophilization.

### HR LC–MS Measurement

For liquid chromatography (LC) coupled to high resolution mass spectrometry (HR-MS), a Bruker Elute UHPLC-system with an Intensity Solo 2 C18-column (100 × 2.1 mm) coupled to a Bruker Impact II ultrahigh resolution Q TOF mass spectrometer with electron-spray ionization (ESI) were used. For LC, water (A) and acetonitrile (B) were used as eluents, both supplemented with 0.1% formic acid, at a flow-rate of 0.3 mL/min. The elution method consisted of equilibration at 5% B for 2 min, a gradient of 5–95% B over 23 min, washing at 95% B for 3 min and re-equilibration at 5% B for 2 min.

### General Procedure for Heterologous Expression and Extraction of Organic Molecules

Culture conditions for heterologous expression experiments were based on those described previously for the pET28b-ptetO-*gfp* vector system.^40,63^ The desired expression plasmids and pET28b-ptetO-*gfp* (empty) as a negative control were individually chemically transformed into *E. coli* BAP1 and selected on an LB-agar plate containing kanamycin. Precultures were inoculated from a single colony, grown in LB-medium o/n and used to inoculate expression cultures with 1% (v/v) in TB, LB or M9-medium. Expression cultures were incubated while shaking at 180 rpm at 37 °C until an OD_600_ of 0.8 for TB and LB cultures or 0.4 for M9 cultures was reached and subsequently cooled to 4 °C for 60 min. Expression was induced by adding 0.5 μg/mL tetracycline and varying amounts of culture supplement when applicable. The cultures were incubated at 20 °C while shaking at 180 rpm in darkness. Test expressions were performed in 100 mL scale in 250 mL Erlenmeyer flasks for 3 and 5 days, respectively; upscaled expressions in 1 L growth medium in 2 L Erlenmeyer flasks.

After incubation, cultures were centrifuged (6000*g* for 15 min) to separate *E. coli* biomass from growth medium. The culture supernatants were adjusted to a pH of 3–4 by addition of conc. HCl and extracted with ethyl acetate (2 × 80 mL per 100 mL of growth medium). The combined extracts were washed with saturated brine, dried over MgSO_4_, and filtered. The solvent was removed under reduced pressure at 40 °C. Dried extracts were redissolved in HPLC-grade methanol and filtered through a syringe driven 0.2 μm PTFE membrane filter (Fisherbrand, USA) prior to HPLC analysis.

### Heterologous Expression and Isolation of Bilothiazoles **A** and **B** (**5** and **6**)

A preculture was inoculated from a cryostock of *E. coli* BAP1 containing the plasmid pET28b-ptetO::7246p1+2+T1_gfp (VI). The expression was supplemented with 125 μM 2,3-DHBA and carried out in 8 L of M9-medium for 3 days. After extraction, the crude extract was purified on a MPLC system with a gradient of 15–40% B over 20 min, where the products eluted between 8 and 13 min. A mixture of **5** and **6** was isolated as yellow-brown oil (30.6 mg; 3.8 mg/L)

### Heterologous Expression and Isolation of Bilothiazoles **C** and **D** (**7** and **8**)

A preculture was inoculated from a cryostock of *E. coli* BAP1 containing the plasmid VI. The expression was supplemented with 250 μM salicylic acid and carried out in 4 L of M9-medium for 5 days. After extraction and evaporation of the organic phase, **7** precipitated as beige crystalline substance and was washed with cold methanol to remove other organic components. 133 mg of **7** were collected (33.3 mg/L). The methanol fraction was purified on an MPLC system with a 20–40% B gradient over 20 min, where **7** and **8** eluted as a mixed fraction between 15 and 18 min. Compound **8** was subsequently purified by preparative HPLC using a gradient from 30–50% B over 20 min where it eluted at 17 min. Compound **8** was isolated as white crystals (15.5 mg; 3.9 mg/L).

### Heterologous Expression and Isolation of Bilothiazole **E** (**9**)

A preculture was inoculated from a cryostock of *E. coli* BAP1 containing the plasmid VI. The expression was supplemented with 250 μM 4-methylsalicylic acid and carried out in 8 L of M9-medium for 5 days. The crude extract was purified on a MPLC system as described before with an adjusted gradient of 20–45% B over 25 min, where the product eluted between 16 and 21 min. Compound **9** was isolated as white crystals (78,2 mg; 9.8 mg/L).

### Heterologous Expression and Isolation of Bilothiazole **F** (**10**)

A preculture was inoculated from a cryostock of *E. coli* BAP1 containing the plasmid VI. The expression was carried out in 4 L M9-medium and supplemented with 125 μM 3-hydroxy picolinic acid. The crude extract was purified on a MPLC system as described before, where a crude product eluted between 10 and 14 min. Final purification ensued by preparative HPLC with a gradient of 35–50% B over 15 min, where the product eluted at 12 min. Compound **10** was isolated as yellow solid (7.5 mg; 1.9 mg/L).

### Specific Rotation

Specific rotations were measured with a Krüss P3000 polarimeter at 20 °C in methanol. Concentrations *c* are given in mg/mL.

### NMR-Measurement

^1^H and ^13^C nuclear magnetic resonance spectra (NMR) were recorded on Bruker AVANCE 300 and AVANCE 600 spectrometers at room temperature. The chemical shifts are given in δ-values (ppm) downfield from TMS and are referenced on the residual peak of the deuterated solvents (DMSO-*d*_6_: δ_H_ = 2.50 ppm, δ_C_ = 39.5 ppm, Methanol-d_4_: δ_H_ = 3.31 ppm, δ_C_ = 49.1 ppm). The coupling constants *J* are given in Hertz [Hz].

### Spot-on-Lawn Assay

Screening for antimicrobial activity of purified bilothiazoles was performed by plate-spreading soft agar inoculated with a bacterial or fungal strain (Gram-positive *B. subtilis* W168, *B. subtilis* subsp. *Spizizenii* ATCC 6633, *S. aureus* ATCC 25923 and *Enterococcus faecalis* ATCC 29212; Gram-negative *E. coli* K12, *P. aeruginosa* ATCC 27853 and *Enterobacter cloacae* ATCC 23355; *P. chrysogenum*). Due to their BSL-2 status, all experiments involving these microorganisms were conducted in a BSL-2 laboratory (Institute for Microbiology, TU Dresden, Dresden, Germany). The overnight cultures of the bacterial strains were inoculated in LB medium. Next, day cultures of the antagonist strains were inoculated 1:250 in fresh LB w/o antibiotic and incubated at 37 °C (220 rpm) until an OD_600_ of around 0.4–0.7 was reached. Ten mL of melted LB soft (0.75%) agar were inoculated with the day cultures to achieve a final OD_600_ of 0.01 and poured onto the surface of the LB plates. The fungal strain *P. chrysogenum* was kept as a spore suspension at −20 °C and used in inoculation of LB soft (0.75%) agar (100 μL in 10 mL LB soft agar). After a drying period of at least 10 min, the microorganism lawn was inoculated with 15 μL of the bilothiazodes (**5**,**6** [5.8 mg/mL]; **7** [6.1 mg/mL]; **8** [7.9 mg/mL]; **9** [7.1 mg/mL] and **10** [7.9 mg/mL]). Fifteen μL of either an antibiotic (positive control; nisin (40 mg/mL) for *B. subtilis* W168 and *B. subtilis* subsp. *Spizizenii* ATCC 6633, ciprofloxacin (200 μg/mL) for all pathogenic bacterial strains, norfloxacin (100 μg/mL) for *E. coli* K12, amphotericin B (250 μg/mL) for *P. chrysogenum*) and a 99.8% (v/v) DMSO solution (negative control) were applied to the surface of the plates after spread coating. Subsequently, another drying period was conducted to allow the pure compounds to be completely absorbed into the agar. Afterward, all plates were incubated upside down overnight at 37 °C for bacterial strains and 28 °C for *P. chrysogenum*. Plates were documented photographically on a black background using a P.CAM360 (1.48× magnification, overhead light level 3).

### Determination of Inhibitory Concentrations

The sensitivity of *B. subtilis* W168, *S. aureus* ATCC 25923 and *E. coli* K-12 toward bilothiazoles were determined in LB medium. Fresh cultures were grown to an optical density (OD_600_) of about 0.5 (mid log) and then diluted to a final OD_600_ of 0.05. Subsequently, 95 μL of the diluted day culture were added to each well and grown in Synergy HTX multimode microplate reader from BioTek (Winooski, USA) at 37 °C with aeration. After 1 h of incubation, serial dilutions (1:2) of the bilothiazodes were prepared and 5 μL of each concentration were added to each well. DMSO (5%, v/v) was used as the negative control. For the determination of inhibitory concentrations of bilothiazoles, the OD_600_ was measured in 5 min intervals for at least 12 h. Microplate reader experiments were performed in biological and technical triplicates.

### Luciferase-Assays in LB-Liquid Medium

The potential molecular functions of the bilothiazoles were determinated using the *B. subtilis* biosensor strains (TMB1617, TMB1619, TMB2120, TMB5611, TMB5830, TMB5831, TMB5845 and TMB5600) harboring pBS3C*lux*-derivates. Therefore, overnight cultures were grown in LB supplemented with chloramphenicol (final concentration 5 μg/mL) for selection. Day cultures, without antibiotics, were inoculated 1:250 in fresh LB-medium and grown to an OD_600_ of 0.3–0.4. Subsequently, cells were diluted to an OD_600_ 0.05 and 95 μL were incubated in a 96-microtiter well plate (black walls, clear bottom, Greiner Bio-One, Frickenhausen, Germany) at 37 °C using a Synergy HTX plate reader (BioTek Intruments GmbH, Bad Friedrichshall, Germany). Luminescence and OD_600_ were measured in 5 min intervals. After 1 h, the biosensor cells were induced with 5 μL of bilothiazoles and antibiotics as a positive control (bacitracin for TMB1617 and TMB1619, nisin for TMB2120, penicillin G for TMB5611, norfloxacin for TMB5830, erythromycin for TMB5831, rifampicin for TMB5845 and amphotericin B for TMB5600), to get final concentrations of 0.3 μg/mL norfloxacin, 0.01 μg/mL penicillin G, 152.5 μg/mL bilothiazole C (**7**), 83.75 μg/mL bilothiazole E (**9**) and 87.5 μg/mL bilothiazole F (**10**). The microplate was subsequently placed back in the microplate reader to continue luminescence and OD_600_ measurements in the aforementioned intervals for 11 h. Quantification of luminescence was achieved by calculating relative luminescence units (RLU), or the luminescence divided by the OD_600_ at a given time point. Visualization of biosensor induction was realized by plotting RLU as a function of time using GraphPad Prism (version 5, San Diego, California). Experiments were performed in biological and technical triplicates.

### Analytical Data

#### Bilothiazole A (**5**)

^1^H NMR (600 MHz, DMSO-*d*_6_): δ8.31 (s, 1H), 7.56 (dd, *J* = 8.0, 1.5 Hz, 1H), 6.93–6.91 (m, 1H), 6.81 (dd, *J* = 15.6, 7.8 Hz, 1H), 5.31 (dd, *J* = 9.6, 8.2 Hz, 1H), 3.62 (ddd, *J* = 19.3, 11.1, 8.9 Hz, 2H) ppm. ^13^C NMR (151 MHz, DMSO-*d*_6_): δ 171.9, 163.8, 163.7, 146.6, 146.0, 144.3, 121.9, 119.6, 119.3, 117.5, 116.7, 78.4, 34.4 Hz. HRMS (ESI^+^) *m*/*z*: 323.0154 [M + H]^+^, calcd, 323.0155.

#### Bilothiazole B (**6**)

^1^H NMR (600 MHz, DMSO-*d*_6_): δ 8.62 (d, *J* = 8.1 Hz, 1H), 8.30 (s, 1H), 7.76–7.74 (m, 1H), 6.93–6.91 (m, 1H), 6.81 (dd, *J* = 15.6, 7.8 Hz, 1H), 4.65 (td, *J* = 7.6, 4.6: Hz, 1H), 3.10–3.00 (m, 2H) ppm. ^13^C NMR (151 MHz, DMSO-*d*_6_): δ 171.6, 163.7, 160.6, 148.0, 146.0, 144.2, 124.6, 119.3, 119.2, 117.9, 116.6, 54.3, 25.4 Hz. HRMS (ESI^+^) *m*/*z*: 341.0258 [M + H]^+^, calcd, 341.0260.

#### Bilothiazole C (**7**)

^1^H NMR (600 MHz, DMSO-*d*_6_): δ 11.26 (br s, 1H), 8.32 (s, 1H), 8.14 (dd, *J* = 7.9, 1.7 Hz, 1H), 7.34 (ddd, *J* = 8.4, 7.3, 1.7 Hz, 1H), 7.05 (dd, *J* = 8.2, 0.8 Hz, 1H), 6.99 (ddd, *J* = 7.9, 7.2, 1.1 Hz, 1H), 5.30 (dd, *J* = 9.6, 8.2 Hz, 1H), 3.67 (dd, *J* = 11.1, 9.8 Hz, 1H), 3.57 (dd, *J* = 11.1, 8.2 Hz, 1H) ppm. ^13^C NMR (151 MHz, DMSO-*d*_6_): δ 171.9, 163.8, 162.9, 155.1, 146.8, 131.5, 127.4, 122.1, 119.7, 118.8, 116.5, 78.45, 34.37 ppm. HRMS (ESI^+^) *m*/*z*: 307.0202 [M + H]^+^: calcd, 307.0206. [α]_D_^20^ + 34.5 (*c* 2.3, MeOH). The spectroscopic data was in agreement to those reported in the literature.^45^

#### Bilothiazole D (**8**)

^1^H NMR (600 MHz, Methanol-*d*_4_): δ 8.24 (s, 1H), 8.20 (dd, *J* = 7.9, 1.6 Hz, 1H), 7.33 (ddd, *J* = 8.3, 7.3, 1.7 Hz, 1H), 7.01 (m, 1H), 6.98 (dd, *J* = 8.0, 0.9 Hz, 1H), 4.86 (dd, *J* = 5.8, 4.7 Hz, 1H), 3.15 (qd, *J* = 14.6, 5.3 Hz, 2H) ppm. ^13^C NMR (151 MHz, Methanol-d_4_): δ 173.0, 166.8, 163.3, 156.9, 149.2, 132.8, 129.1, 125.2, 120.9, 120.0, 117.5, 55.70, 26.82 ppm. HRMS (ESI^+^) *m*/*z*: 325.0308 [M + H]^+^, calcd, 325.0311.

#### Bilothiazole E (**9**)

^1^H NMR (600 MHz, DMSO-*d*_6_): δ 11.15 (br s, 1H), 8.27 (s, 1H), 8.01 (d, *J* = 8.0 Hz, 1H), 6.85 (s, 1H), 6.81 (dd, *J* = 8.2, 2.1 Hz, 1H), 5.29 (dd, *J* = 9.7, 8.2 Hz, 1H), 3.66 (dd, *J* = 11.1, 9.7 Hz, 1H), 3.57 (dd, *J* = 11.2, 8.2 Hz, 1H), 2.29 (s, 3H) ppm. ^13^C NMR (151 MHz, DMSO-*d*_6_): δ 171.9, 163.8, 163.3, 155.1, 146.7, 141.7, 127.3, 121.5, 120.7, 116.8, 116.3, 78.45, 34.36, 21.12 ppm. HRMS (ESI^+^) *m*/*z*: 321.0360 [M + H]^+^, calcd, 321.0362. [α]_D_^20^ + 27.0 (*c* 7.8, MeOH).

#### Bilothiazole F (**10**)

^1^H NMR (600 MHz, DMSO-*d*_6_): δ 11.27 (br s, 1H), 8.52 (s, 1H), 8.26–8.24 (m, 1H), 7.58–7.55 (m, 1H), 7.49 (dd, *J* = 8.5, 4.5 Hz, 1H), 5.36 (dd, *J* = 9.6, 8.2 Hz, 1H), 3.70 (ddd, *J* = 19.3, 11.2, 9.0 Hz, 2H) ppm. ^13^C NMR (151 MHz, DMSO-*d*_6_): δ 171.6, 169.5, 162.2, 152.2, 147.1, 141.5, 134.1, 127.2, 125.4, 123.8, 78.43, 34.85 ppm. HRMS (ESI^+^) *m*/*z*: 308.0158 [M + H]^+^, calcd, 308.0158. [α]_D_^20^ + 4.1 (*c* 4.9, MeOH).

## Supporting Information Available

Lists of strains, plasmids, oligonucleotides, genes and substrates; gene cluster annotation; gel photos for cloning procedure; plasmid sequencing results; LC–MS data; bioactivity results; NMR-spectra (PDF)
