# Supplementary material for: Heterologous Expression of a Cryptic BGC from Bilophila sp. Provides Access to a Novel Family of Antibacterial Thiazoles
Source: ACS Synth Biol. 2025 Feb 25;14(3):967–78. doi: 10.1021/acssynbio.5c00042 (PMC11934131; doi:10.1021/acssynbio.5c00042)
Supplement: Supplementary file 1 — sb5c00042_si_001.pdf [file sb5c00042_si_001.pdf]

Heterologous Expression of a Cryptic BGC from *Bilophila sp.* Provides Access to a Novel Family of Antibacterial Thiazoles

Maximilian Hohmann,[1] Denis Iliasov,[2] Martin Larralde,[3] Widya Johannes,[4] Klaus-Peter Janßen,[4] Georg Zeller,[5] Thorsten Mascher,[2] Tobias A. M. Gulder[1,6]*

[1] Chair of Technical Biochemistry, TUD Dresden University of Technology, Bergstraße 66, 01069 Dresden, Germany.

[2] General Microbiology, TUD Dresden University of Technology, Zellescher Weg 20b, 01217 Dresden, Germany.

[3] Leiden University Center for Infectious Diseases (LUCID), Leiden University Medical Center, 2333 ZA Leiden, Netherlands.

[4] Department of Surgery, School of Medicine and Health, Klinikum rechts der Isar, Technical University of Munich, 81675 Munich, Germany.

[5] Leiden University Center for Infectious Diseases (LUCID) and Center for Microbiome Analyses and Therapeutics (CMAT), Leiden University Medical Center, 2333 ZA Leiden, Netherlands.

[6] Department of Natural Product Biotechnology, Helmholtz Institute for Pharmaceutical Research Saarland (HIPS), Helmholtz Centre for Infection Research (HZI) and Department of Pharmacy, PharmaScienceHub (PSH), Saarland University, Campus E8.1, 66123 Saarbrücken, Germany.

* E-mail: tobias.gulder@helmholtz-hips.de

SUPPORTING INFORMATION

Table of Contents

Supplementary Tables....2

Supplementary Figures....8

NMR-Data....23

Bilothiazoles A, B (**5**, **6**)....23

Bilothiazole C (**7**)....27

Bilothiazole D (**8**)....30

Bilothiazole E (**9**)....33

Bilothiazole F (**10**)....36

Supplementary References....39

Supplementary Tables

Table S1. Bacterial strains and plasmids used in this study.

**Strains**	**Description**[A]	**Reference/Source**
Escherichia. coli DH5α	Host strain for cloning	NEB
E. coli BAP1	Heterologous expression strain	Pfeifer *et al*., 2001[1]
E. coli K-12	F- lambda- *ilvG*- *rfb*-50 *rph*-1	Laboratory stock
Staphylococcus aureus ATCC 25923	Wildtype strain, facultatively pathogenic	Laboratory stock
Bacillus subtilis W168	Wild type; *trpC2*	Laboratory stock
B. subtilis subsp. *spizizenii* ATCC 6633	Wildtype strain, Subtilin producer	Laboratory stock
Enterococcus faecalis ATCC 29212	Wildtype strain, facultatively pathogenic	Laboratory stock
Pseudomonas aeruginosa ATCC 27853	Wildtype strain, facultatively pathogenic	Laboratory stock
Enterobacter cloacae ATCC 23355	Wildtype strain, facultatively pathogenic	Laboratory stock
Penicillium chrysogenum	Wildtype strain	Laboratory stock
TMB5611	*B. subtilis* W168 *penP*::*kan*^{R} *thrC*::pBS4S-P_{lepA}_*blaI* *lacA*::pBS2E-P_{veg}_*blaR1* pBS3C-P_{blaZ}-*luxABCDE*, *cm*^{R}	Lautenschläger *et al*. 2020[2]
TMB5830	*B. subtilis* W168 *sacA*::*cm*^{R}, P_{recA}- *luxABCDE*	Milzarek *et al*. 2023[3]
**Plasmids**	**Description**	**Reference/Source**
pET28b-ptetO-*gfp* (6552 bp)	Tetracycline inducible expression plasmid ColE1, Kan^R, *gfp* reporter gene downstream of promotor	Duell *et al*., 2019[4]

[A] - *cm*^{R}, chloramphenicol resistance; *kan*^{R}, kanamycin resistance

pET28b-ptetO::7246p2_*gfp* (16502 bp, **I**)	pET28b-ptetO-*gfp*v2 with genes K and L of the 7246 cluster cloned between $Ptet_O$ and *gfp*	Gene synthesis (Azenta)
pUC-GW-Amp::7246p1 (9361 bp)	pUC-GW-Amp vector (Amp^R) with gene J of the 7246 cluster	Gene synthesis (Azenta)
pTwist_Chlor::7246::T1 (6984 bp)	pTwist Chlor vector (Cm^R) with genes A, B, E & I of the 7246 cluster	Gene synthesis (Twist Bioscience)
pTwist_Chlor::7246::T2 (6991 bp)	pTwist Chlor vector (Cm^R) with genes F, G, H, M & N of the 7246 cluster	Gene synthesis (Twist Bioscience)
pET28b-ptetO::7246p1+2_*gfp* (23182 bp, **II**)	pET28b-ptetO-*gfp*v2 with genes J, K and L of the cluster 7246 cloned between $Ptet_O$ and *gfp*	This study
pET28b-ptetO::7246p1+2+T1_*gfp* (28141 bp, **III**)	pET28b-ptetO-*gfp*v2 with genes A, B, E, I, J, K & L of the cluster 7246 cloned between $Ptet_O$ and *gfp*	This study
pET28b-ptetO::7246p1+2+T2_*gfp* (28158 bp, **IV**)	pET28b-ptetO-*gfp*v2 with genes *J, K, L, F, G, H, M* & *N* of the cluster 7246 cloned between $Ptet_O$ and *gfp*	This study
pET28b-ptetO::7246p1+2+T1+2_*gfp* (33111 bp, **V**)	pET28b-ptetO-*gfp*v2 with the full 7246 cluster (genes *A, B, E, I, J, K, L, F, G, H, M* & *N*) cloned between $Ptet_O$ and *gfp*	This study

Table S2. List of oligonucleotides used for cloning and screening purposes.

Name	Sequence (5’ → 3’)	Description
screen_7246_p1_R	GCCCAATCCAATAAGCATAC	Colony screening
screen_7246_p1_F	CCTGATTATATGGTACCGAGCG	Colony screening
screen_7246_p2_R	CACCAGGCTACTGCTGCAC	Colony screening
screen_7246_p2_F	CTATGAAATGGCGGTGCAGC	Colony screening
screen_7246_TailoringI_F	GTCCTGGGGTGTAAAATGG	Colony screening
screen_7246_TailoringI_R	CTCGTAAGACAGCTTTTGAC	Colony screening
screen_7246_TailoringII_R	CTTCATCTCCAACCGCTTCG	Colony screening
Screen_ptetF2	TCCGACCTCATTAAGCAGC	Colony screening
Screen_GFP_R	TTACCGTTGGTCGCATCACC	Colony screening
gib_7246-TailoringI::ptet_F	<u>GAGGATCGACCCATGGAATTCAGT</u>ATGTTCCCATCATCTGGAATTG	Forward primer for amplification of genes A, B, E & I with <u>homology arms</u>
gib_7246-TailoringI::NRPSp1_R	<u>CAATGACCGCCGGATCTTTCATAGT</u>TTACACTTTACGTCCTGCATATC	Reverse primer for amplification of genes A, B, E & I with <u>homology arms</u>
gib_7246-p2_T2_F	<u>GATTGGCAGCGTGAAATAAGGATC</u>ATGTCAGATAATGTGAGAAG	Forward primer for amplification of genes F, G, H, M & N with <u>homology arms</u>
gib_ptet_7246_T2_BamHI_R	<u>CACCTTTGCTAACCATGG**GGATCC**</u>TCATTCCATCATGTTGG	Reverse primer for amplification of genes F, G, H, M & N with <u>homology arms</u> and **BamHI restriction site**

Table S3. Predicted functions of gene products of the *bil* BGC based on BLAST searches.

Protein	Size [aa]	Accession-Nr	Closest Characterized Homolog (Identity, Organism, Accession-Nr.)	Function of Homolog / Proposed Function
BilA	303	WP_302551529.1	CysE (50.0%, *Phocaeicola vulgatus*, QEW34739.1)	Serine acetyltransferase
BilB	240	WP_302551530.1	LgrE (42.6%, *Brevibacillus parabrevis*, WDV98005.1)	Type II thioesterase subunit
BilC	241	WP_302551531.1	-	4'-Phosphopantetheinyl transferase
BilD	139	WP_302551532.1	-	HIT-family protein
BilE	569	WP_302551533.1	-	2,3-Diaminopropionate-AMP-ligase
BilF	344	WP_302551534.1	DctP (28.8%, *Sagittula stellata*, WP_005857562.1)	TRAP dicarboxylate transporter
BilG	166	WP_302551535.1	-	TRAP transporter
BilH	433	WP_302551536.1	-	TRAP transporter
BilI	534	WP_302551537.1	DhbE (46.4%, *B. subtilis*, AAN15214.1)	2,3-Dihydroxybenzoate-AMP ligase
BilJ	2223	WP_302551538.1	-	Non-ribosomal peptide synthetase
BilK	1558	WP_302551539.1	-	Polyketide synthetase
BilL	1750	WP_302551540.1	Irp2 (33.0%, *Yersinia pestis*, CAA21390.1)	Yersiniabactin non-ribosomal peptide synthetase
BilM	253	WP_302551541.1	PikAV (32.2%, *Streptomyces venezuelae*, AAC69333.1)	Type II thioesterase
BilN	456	WP_302551542.1	Ccr (50.7%, *Streptomyces collinus*, AAA92890.1)	Crotonyl-CoA-Reductase

Table S4. Substrate-scope of the *bil* BGC.

Entry	Substrate	Production Titer	Comment
1	Benzoic acid	<1 mg/L	Not sufficient for NMR, for mass see Figure S8
2	Salicylic acid	33.3 mg/L	**7** and **8**
3	3-Hydroxy benzoic acid	In traces	Figure S9
4	4-Hydroxy benzoic acid	No product	
5	2,3-Dihydroxy benzoic acid	3.8 mg/L	Mixture of **5** and **6**
6	2,4-Dihydroxy benzoic acid	No product	
7	3,4-Dihydroxy benzoic acid	No product	
8	3,5-Dihydroxy benzoic acid	No product	
9	Gallic acid	No product	
10	2,4,6-Trihydroxy benzoic acid	No product	
11	2,3,4-Trihydroxy benzoic acid	No product	
12	4-Methyl salicylic acid	9.8 mg/L	**9**
13	Anthranilic acid	No product	
14	4-Amino benzoic acid	No product	
15	4-Amino salicylic acid	In traces	Figure S10
16	3-Hydroxy anthranilic acid	No product	
17	Vanillic acid	In traces	Figure S11
18	*Ortho*-vanillic acid	In traces	Figure S12
19	3-Hydroxy picolinic acid	1.9 mg/L	**10**

Table S5. Results of bioactivity testing bilothiazoles A-F (**5**-**10**).

**Microbial Strains**	**MIC (μg/mL)**				
	**5,6**	**7**	**8**	**9**	**10**
B. subtilis W168	290	153	>300	83.8	87.5
E. coli K12	>300	>300	>300	>300	>300
Enterobacter cloacae	>300	>300	>300	>300	>300
Enterococcus faecalis	>300	>300	>300	>300	>300
P. aeruginosa	>300	>300	>300	>300	>300
S. aureus	>300	>300	>300	168	>300
P. chrysogenum	>300	>300	>300	>300	>300
**Cell Line**	**IC_{50} (μM)**				
	**5,6**	**7**	**8**	**9**	**10**
HCT116	-	100	>100	-	-

Supplementary Figures

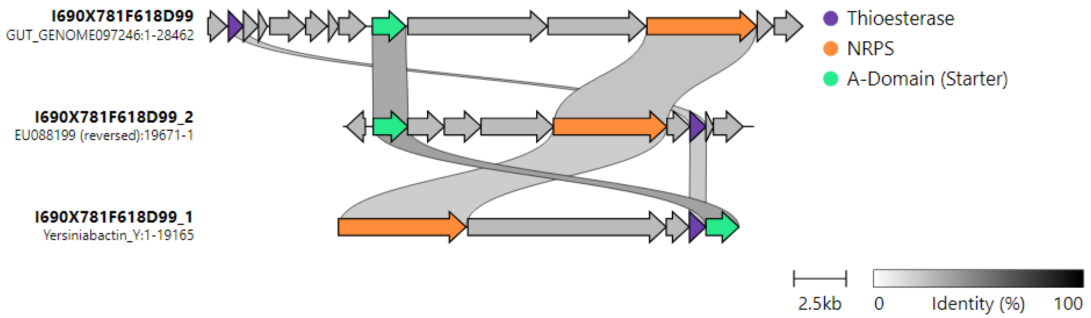

Figure S1. Cluster similarity analysis of 7246- (upper), pyochelin (middle, Genbank accession EU088199) and yersiniabactin (Genbank accession AM236324.1.) BGCs (analysis with Clinker).

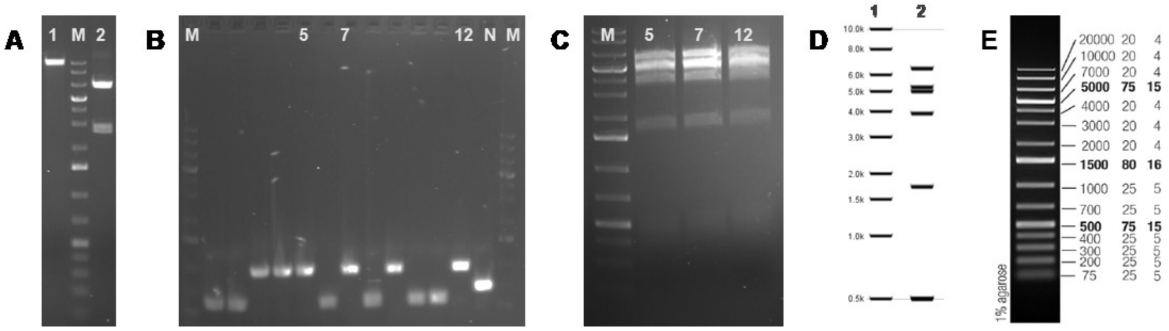

Figure S2. Gel photos for the cloning process of construct V. **A**. 1 – Linearized construct I, digested with EcoRI (16.5 kb); M – DNA-Marker; 2 – Digestion of pUC-GW-Amp::7246p1 with EcoRI, size of insert: 6.7 kb. **B**. Colony-screening of ligation products with primers screen_7246_p1_F, screen_7246_p2_R and screen_ptetF2 to distinguish positive clones (1 kb) from clones where the insert was inserted in the wrong direction (0.6 kb), from the negative control (0.7 kb). M – DNA-Ladder; 5, 7, 12 – selected positive clones, N – negative control. **C**. Restriction digests of positive clones 5, 7 and 12 with NdeI. M – DNA-Ladder. **D**. 1 – DNA-Ladder for virtual digest; 2 – Virtual digest with NdeI. **E**. Annotation of DNA-Ladder.

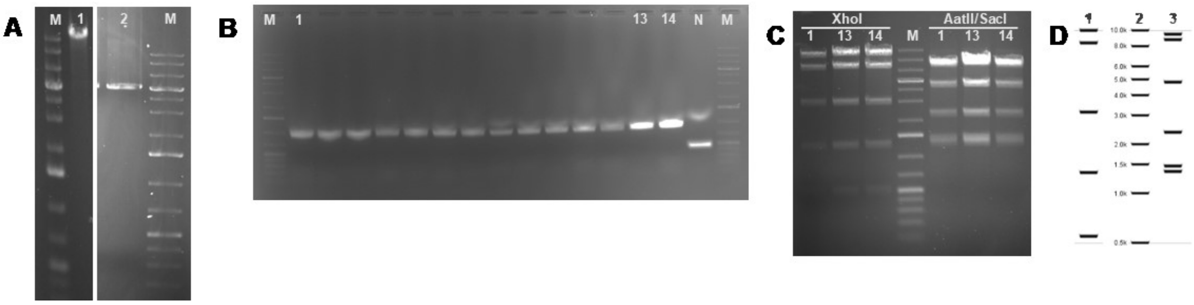

Figure S3. Gel photos for the cloning process of construct VI. **A**. M – DNA-Marker; 1 – Linearized construct V, digested with ScaI (23.1 kb); 2 – PCR-amplification of genes *A*, *B*, *E* and *I* with Q5-polymerase and primers containing homology arms for SLIC-assembly into the linearized construct II. **B**. Colony screening of SLIC-transformants with primers screen_7246_TailoringI_F and screen_7246_p1_R to distinguish positive (1.0 kb) from negative (0.5 kb) clones. M – DNA-Marker, 1, 13, 14 – selected positive clones, N – negative control. **C**. Restriction digests of positive clones 1, 13 and 14 with XhoI and AatII/SacI, M – DNA-Marker. **D**. 1 – virtual digest with XhoI, 2 – DNA-Ladder, 3 – virtual digest with AatII and SacI.

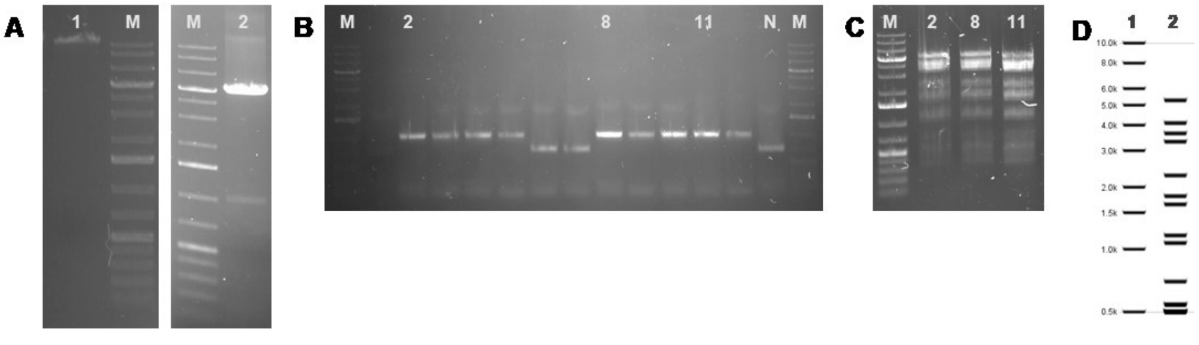

Figure S4. Gel photos for cloning procedure of construct VII. **A**. M – DNA-Marker, 1 – Linearized construct V, digested with BamHI (23.1 kb), 2 – PCR-amplification of genes *F*, *G*, *H*, *M* and *N* with Q5-polymerase and primers containing homology arms for SLIC-assembly into the linearized construct II (5.0 kb). **B**. Colony screening of SLIC-transformants with primers screen_7246_TailoringII_R and screen_7246_p2_F to distinguish positive (1.0 kb) from negative (0.7 kb) clones. M – DNA-Marker, 2, 8, 11 – selected positive clones, N – negative control. **C**. Restriction digests of positive clones 2, 8 and 11 with AvaI, M – DNA-Marker. **D**. 1 – DNA-Marker for virtual digest, 2 – virtual digest with AvaI.

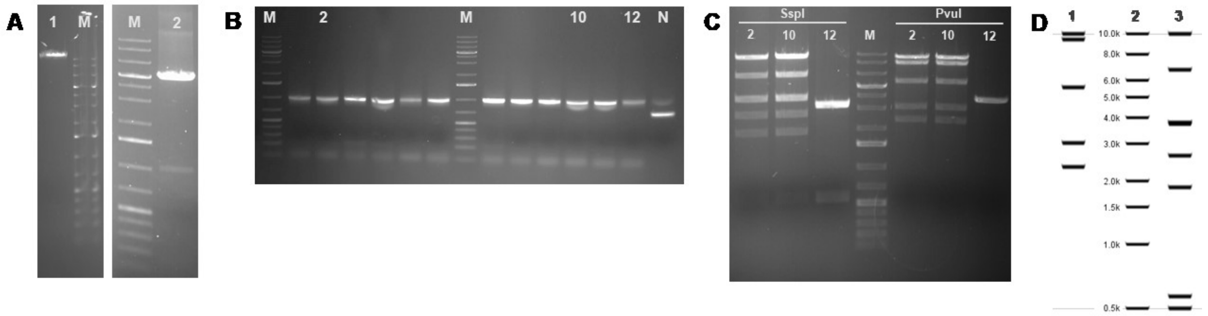

Figure S5. Gel photos for cloning procedure of construct VIII. **A**. M – DNA-Marker, 1 – Linearized construct VI, digested with ScaI (28.1 kb), 2 – PCR-amplification of genes *F*, *G*, *H*, *M* and *N* with Q5-polymerase (same as in Figure S4A). **B**. Colony screening of SLIC-transformants with primers screen_7246_TailoringII_R and screen_7246_p2_F to distinguish positive (1.0 kb) from negative (0.7 kb) clones. M – DNA-Marker, 2, 10, 12 – selected positive clones, N – negative control. **C**. Restriction digests of positive clones 2 and 10 and negative clone 12 with SspI and PvuI, M – DNA-Marker. **D**. 1 – virtual digest with SspI, 2 – DNA-Marker for virtual digest, 3 – virtual digest with PvuI.

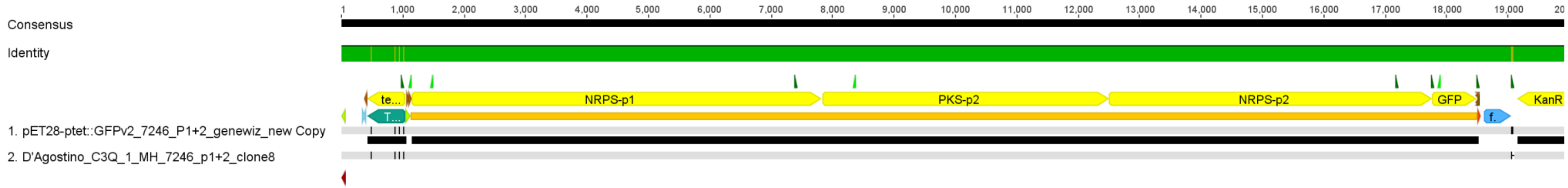

Figure S6. Schematic representation of the plasmid sequencing results of construct V (clone 8). The pairwise identity within the insert region is 100%.

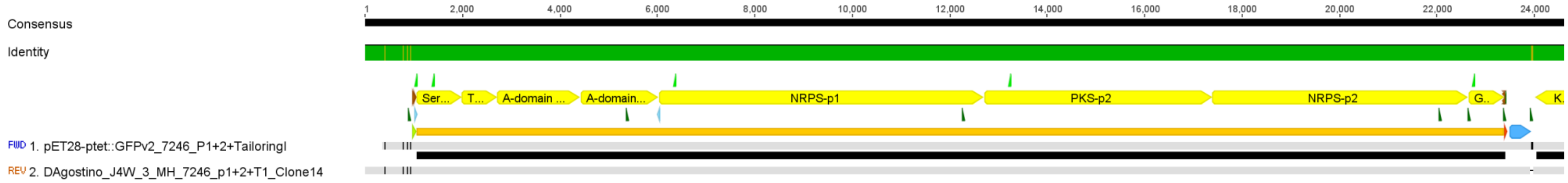

Figure S7. Schematic representation of the plasmid sequencing results of construct VI (clone 14). The pairwise identity within the insert region is 100%.

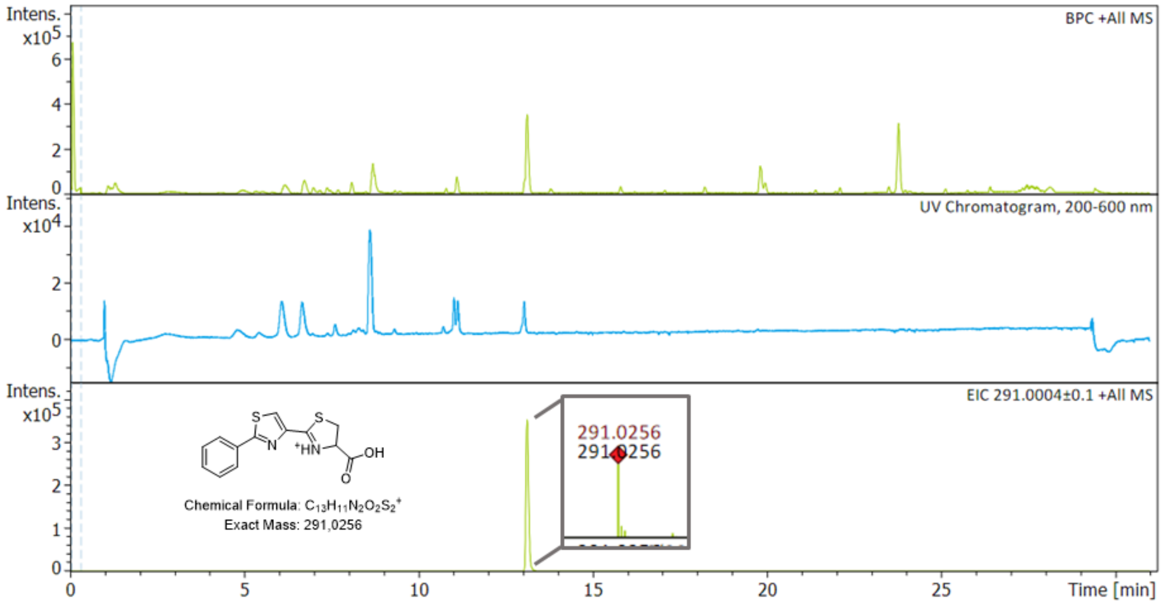

Figure S8. LC-MS analysis of extract of expression of construct VI, supplemented with benzoic acid. The product is identified in significant amounts, however the yield is not sufficient for purification: 291.0256 [M+H]+, calc.: 291.0256.

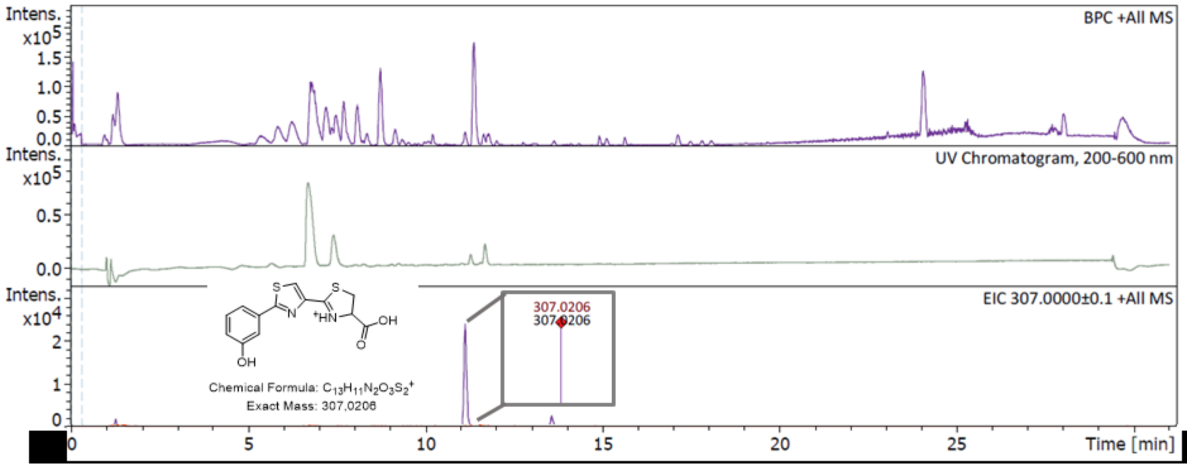

Figure S9. LC-MS analysis of extract of expression of construct VI, supplemented with 3-hydroxy benzoic acid. The product is identified in traces and can be distinguished from the background signal of **3** (13.5 min peak) due to the shorter retention time (11 min). The mass is identical to **3**: 307.0206 [M+H]+, calc.: 307.0206.

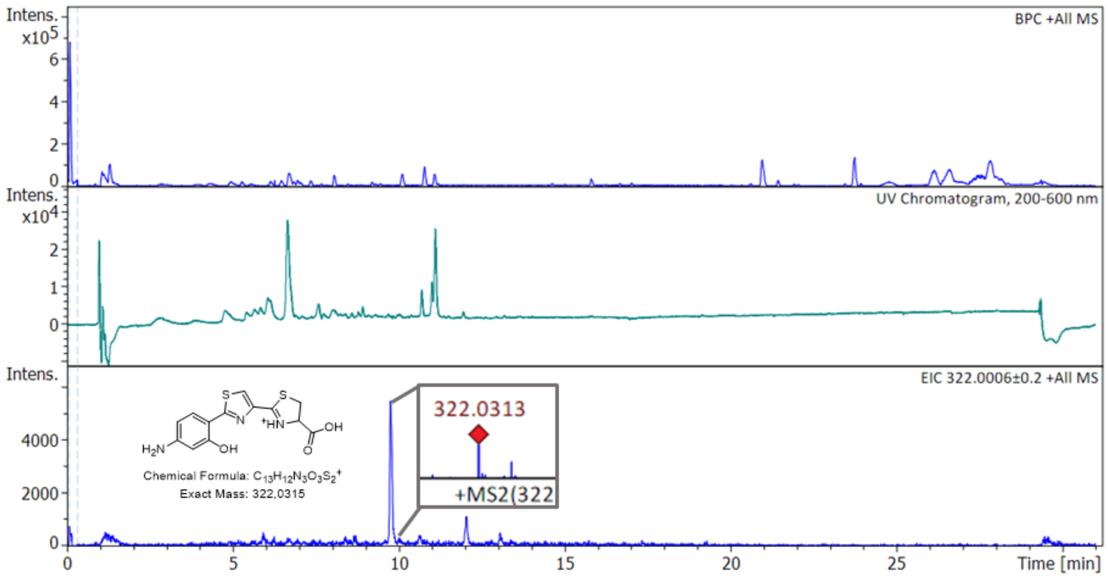

Figure S10. LC-MS analysis of extract of expression of construct VI, supplemented with 4-amino salicylic acid. The product is identified in traces: 322.0313 [M+H]$^+$, calc.: 322.0315.

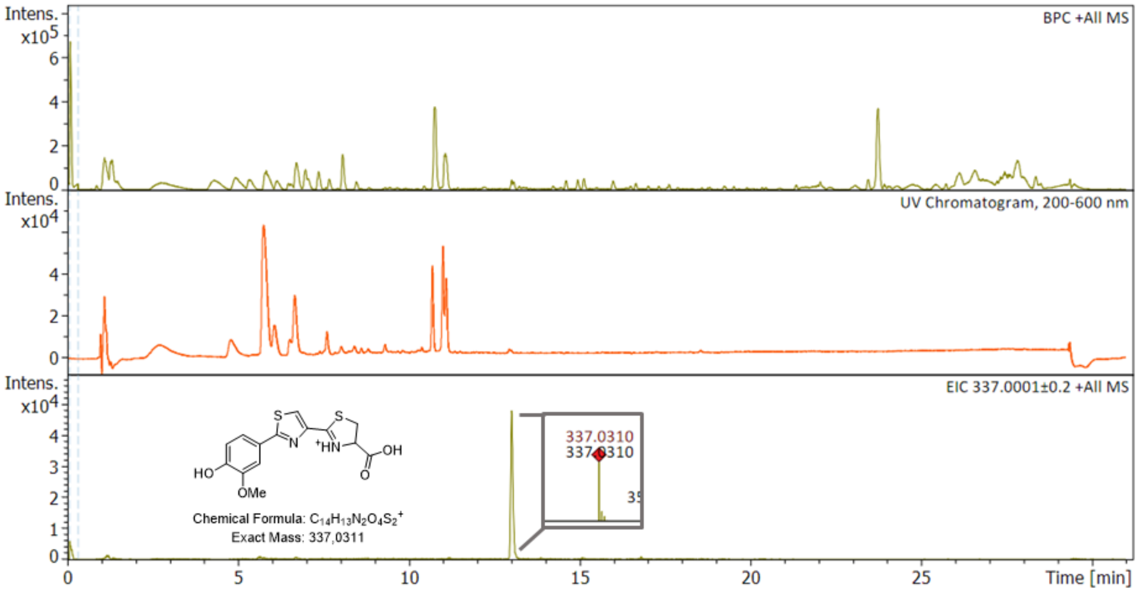

Figure S11. LC-MS analysis of extract of expression of construct VI, supplemented with vanillic acid. The product is identified in traces: 337.0310 [M+H]+, calc.: 337.0311.

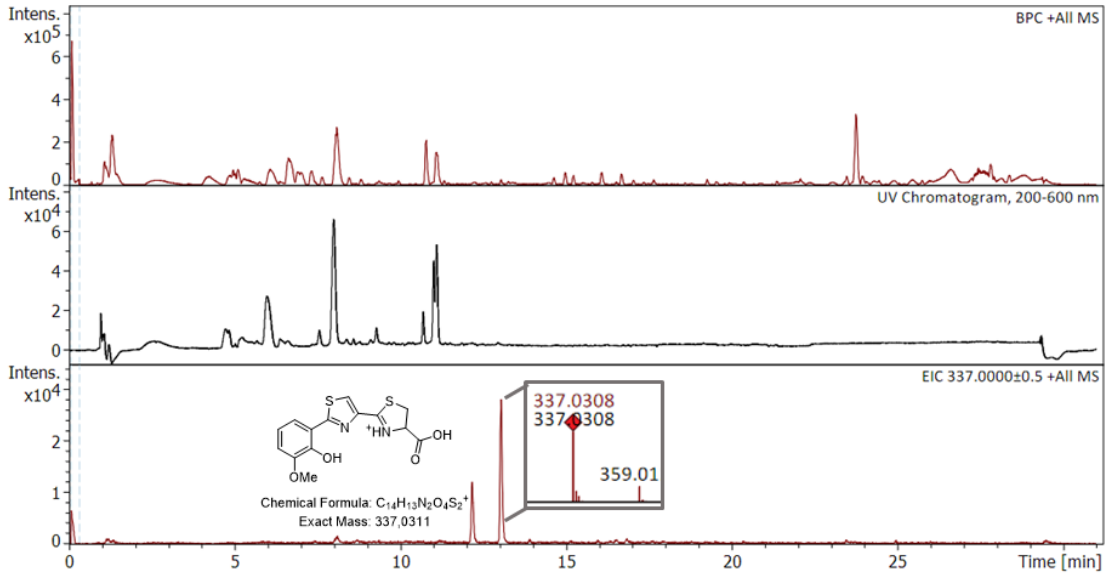

Figure S12. LC-MS analysis of extract of expression of construct VI, supplemented with *ortho*-vanillic acid. The product is identified in traces: 337.0308 [M+H]+, calc.: 337.0311.

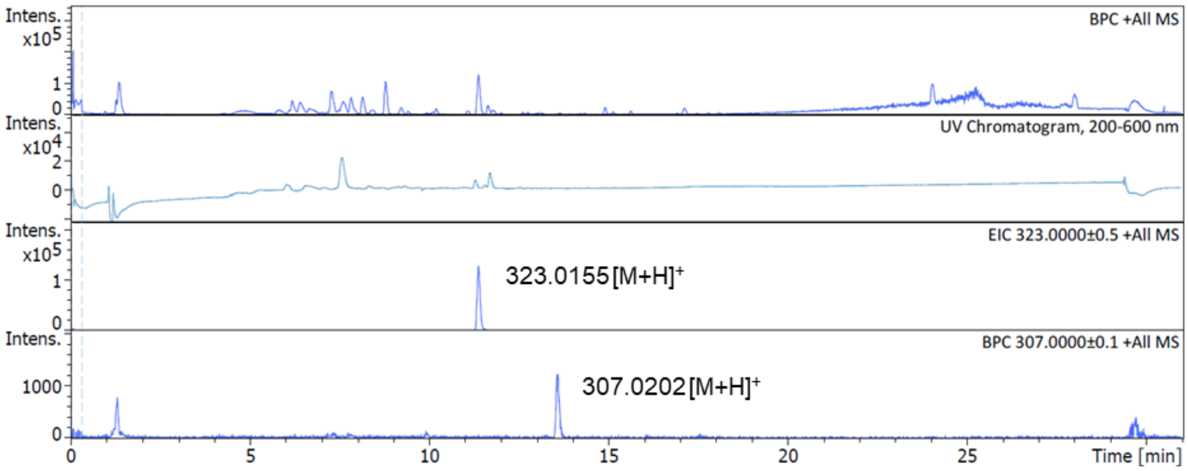

Figure S13. LC-MS analysis of unsupplemented expression of the 7246 BGC after 3 days in M9 medium. **5** (calc. [M+H]+ = 323.0155) was found in higher abundance than **7** (calc. [M+H]+ = 307.0206).

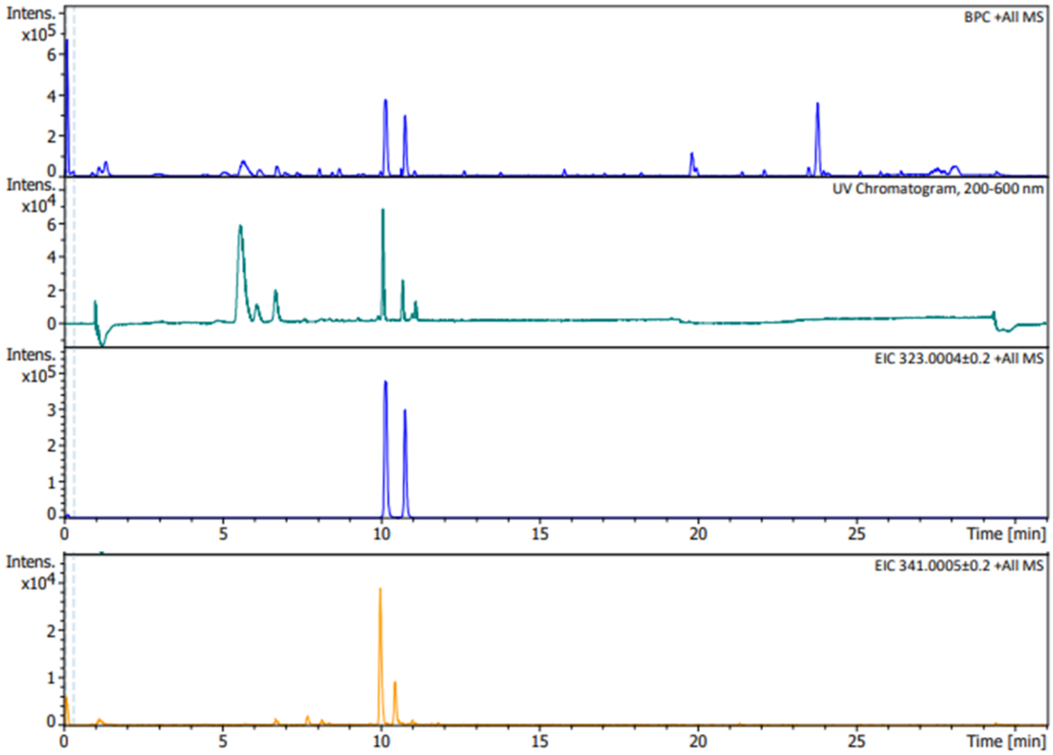

Figure S14. HR-MS spectra of raw extract of expression supplemented with 2,3-DHBA. The abundances were determined as 4×10^5 for **5** and 3×10^4 for **6** which equals a ratio of 13:1.

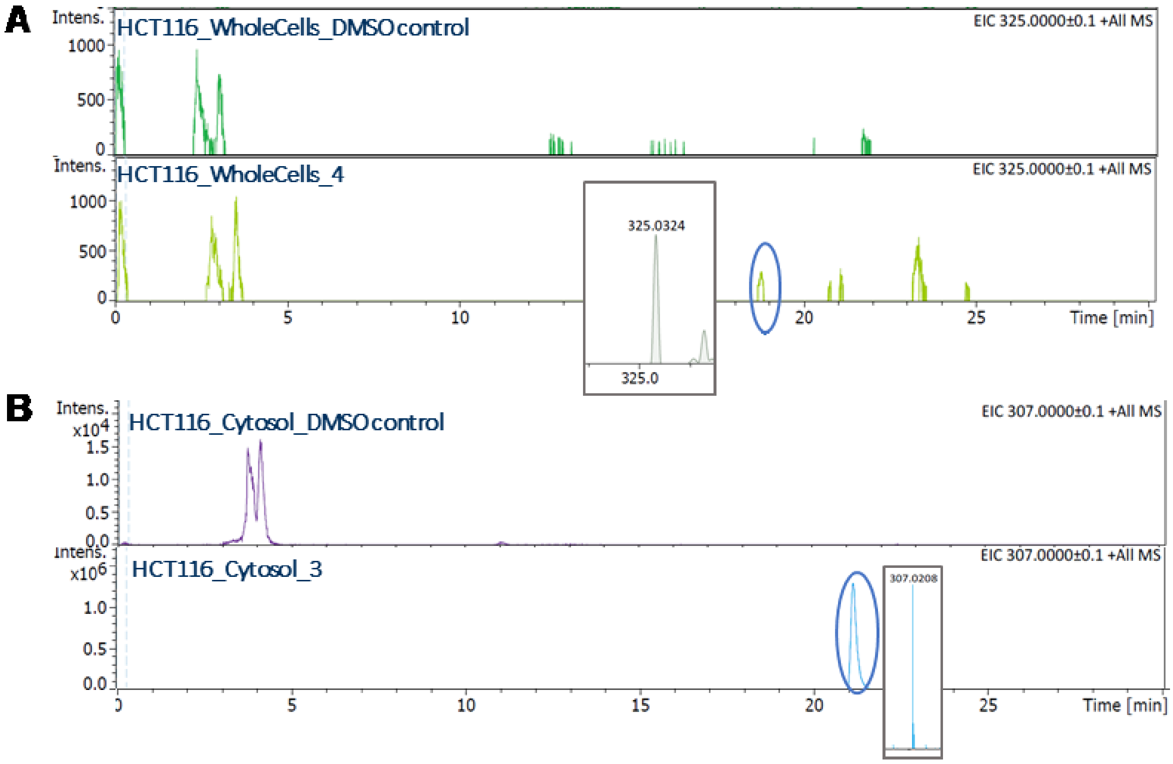

Figure S15. LC-MS measurements of fractionated HCT116 cell samples. **A**: compound **4** was found only in traces in the whole cell fractions, indicating severely limited uptake. **B**: high concentrations of compound **3** were selectively taken up into the cytosol of HCT116 cells. Only traces could be identified in nucleic fractions.

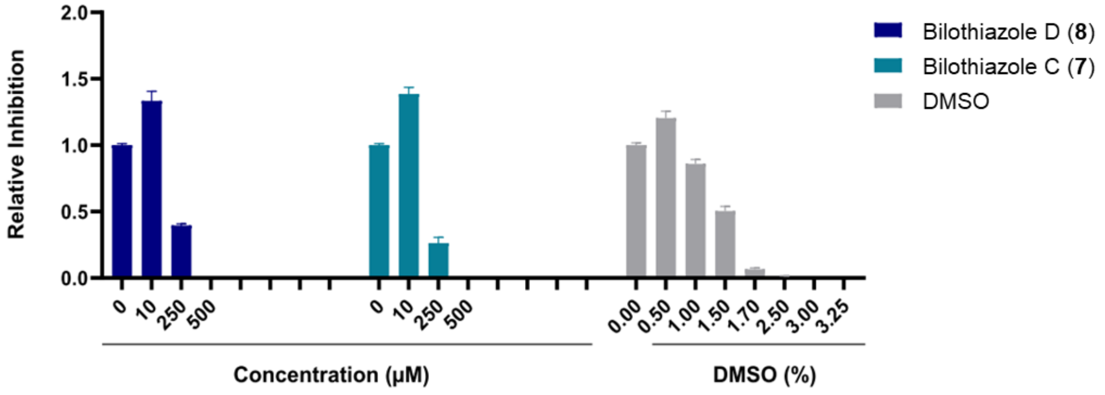

Figure S16. Quantification of clonogenic assays shows weak cytotoxicity of **7** and **8** at >250 μM concentrations (t = 7 d).

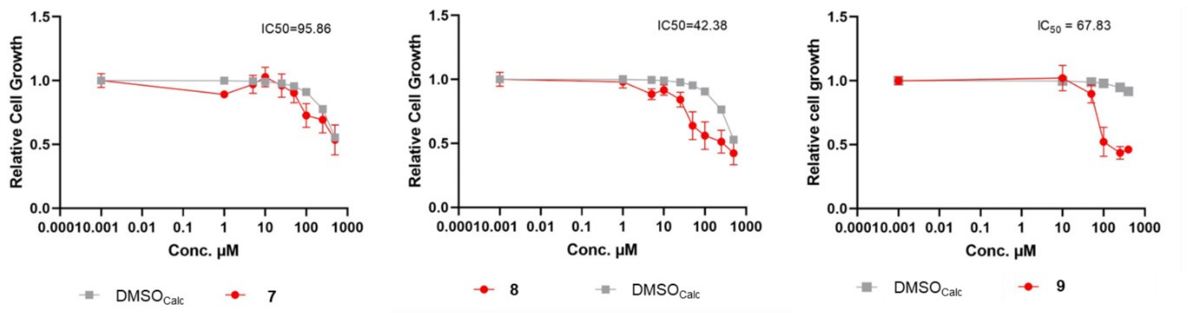

Figure S17. SRB-Assays for quantification of cytotoxicity of bilothioazoles **7**–**9** against HCT116 showed only weak cytotoxic activities.

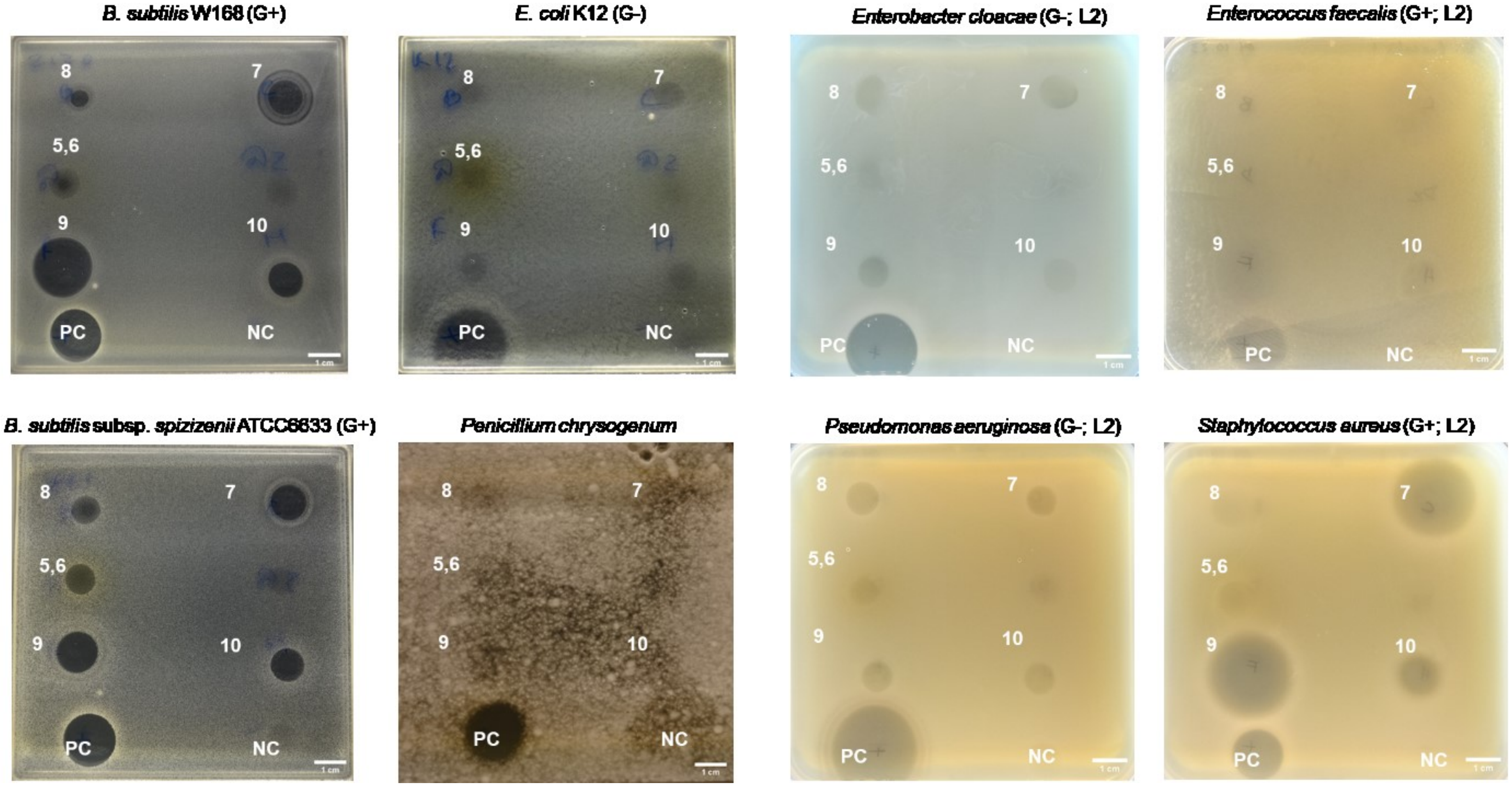

Figure S18. Overlay assays against a range of bacteria and fungi for initial determination of bioactivity of the bilazoles A–F (**5**–**10**).

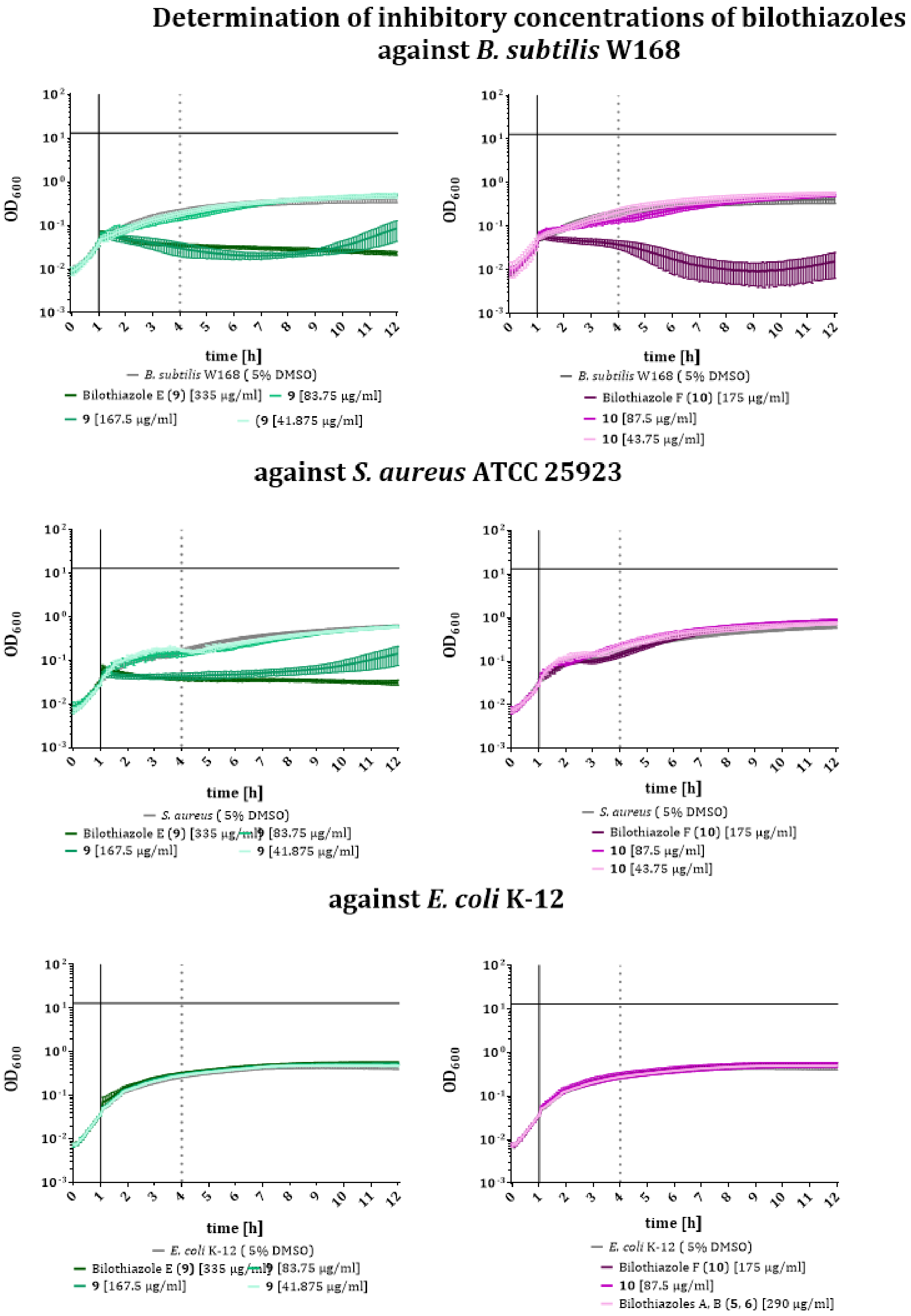

Figure S19. Determination of inhibitory concentrations of bilothiazoles against *B. subtilis* W168 and *S. aureus* ATCC 25923. Exponential growth phase cells of *B. subtilis* W168 and *S. aureus* ATCC 25923 were induced with defined concentrations of **9** and **10** and DMSO (negative control). Addition of 5 µL of the samples to the strains is indicated at time point 1 hour (solid black line). Dotted black lines indicate time point at which OD600 averages were calculated (4 h).

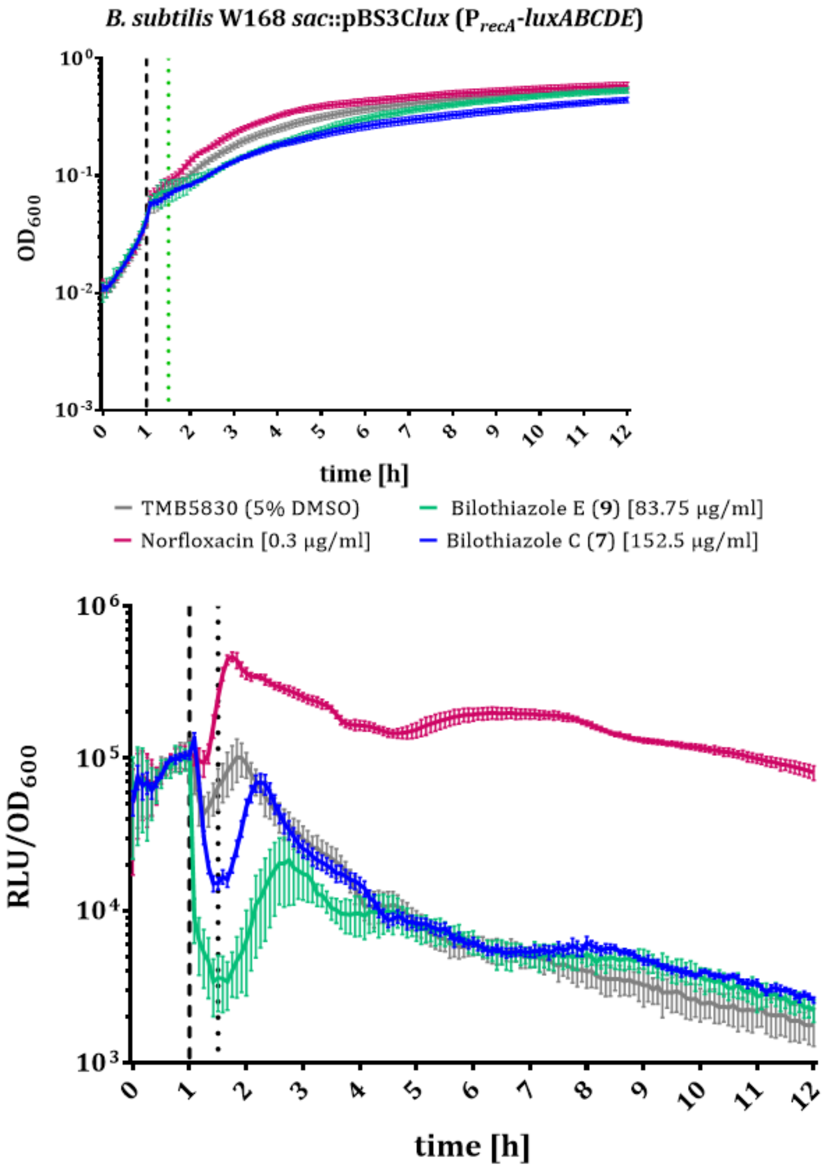

Figure S20. Induction assay of the biosensor TMB5830 (P_{recA}-lux) with **7** and **9**. Exponential growth phase cells of TMB5830 (*B. subtilis* W168, P_{recA}-lux) were induced with **7** and **9** and norfloxacin (positive control). Induction with DMSO was used as negative controls. Relative luminescence (RLU) proposing recA-promoter activity is shown 30 min after induction. All tested bilothiazoles potentially repressed P_{recA}-activity (>5-fold). Addition of 5 µL of the samples to the strains is indicated at time point 1 h (solid black line). Dotted black lines indicate time point at which OD_{600} averages were calculated (4 h).

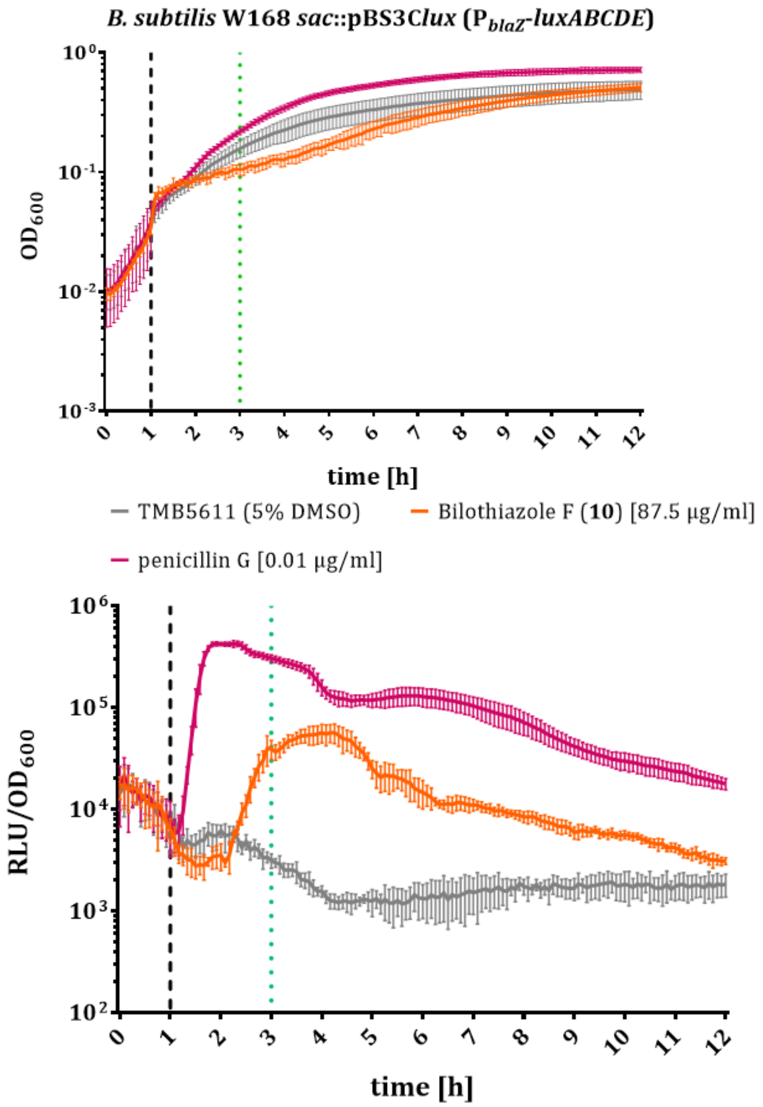

Figure S21. Induction assay of the biosensor TMB5611 (P_{blaZ}-lux) with **10**. Exponential growth phase cells of TMB5611 (*B. subtilis* W168, P_{blaZ}-lux) were induced with defined concentrations of **10** and penicillin G (positive control). Induction with DMSO was used as negative control. Relative luminescence (RLU) proposing blaZ-promoter activity is shown 2 h after induction. Compound **10** potentially induced P_{blaZ}-activity (~10-fold). Addition of 5 µL of the samples to the strains is indicated at time point 1 h (solid black line). Dotted black lines indicate time point at which OD_{600} averages were calculated (4 h).

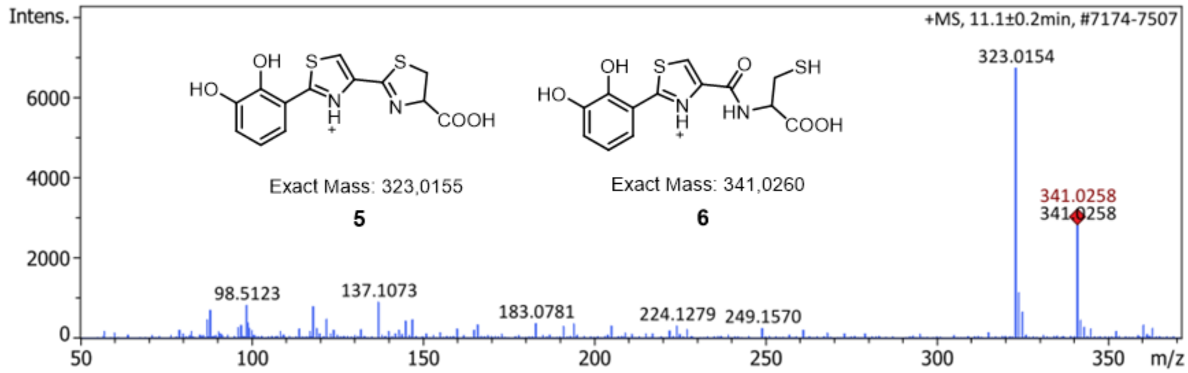

Figure S22. HR-ESI-MS spectrum of mixture of compounds **5** $[M+H]^+$ (323.0154 m/z) and **6** $[M+H]^+$ (341.0258 m/z).

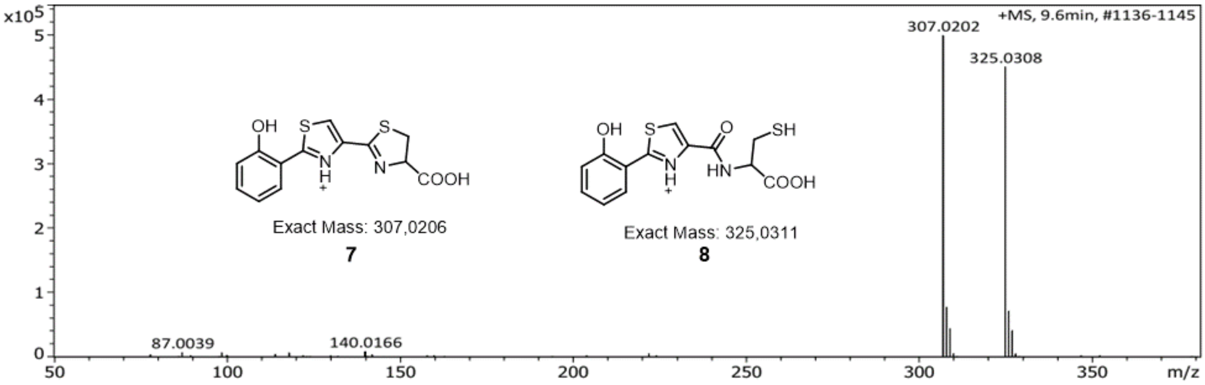

Figure S23. HR-ESI-MS spectrum of mixture of compounds **7** $[M+H]^+$ (307.0202 m/z) and **8** $[M+H]^+$ (325.0308 m/z).

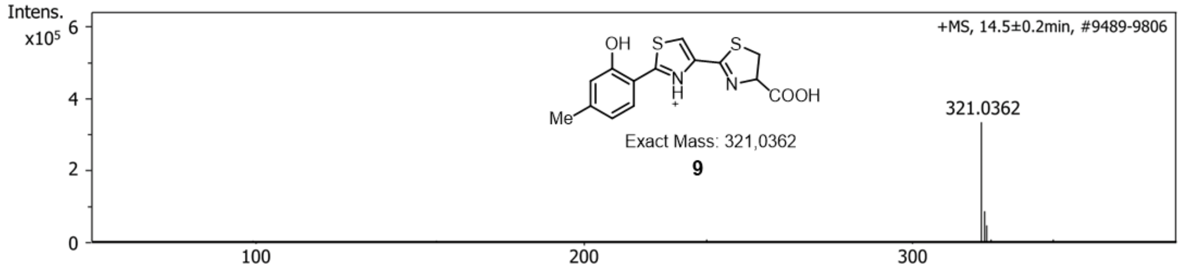

Figure S24. HR-ESI-MS spectrum of compound **9** $[M+H]^+$ (321.0362 m/z)

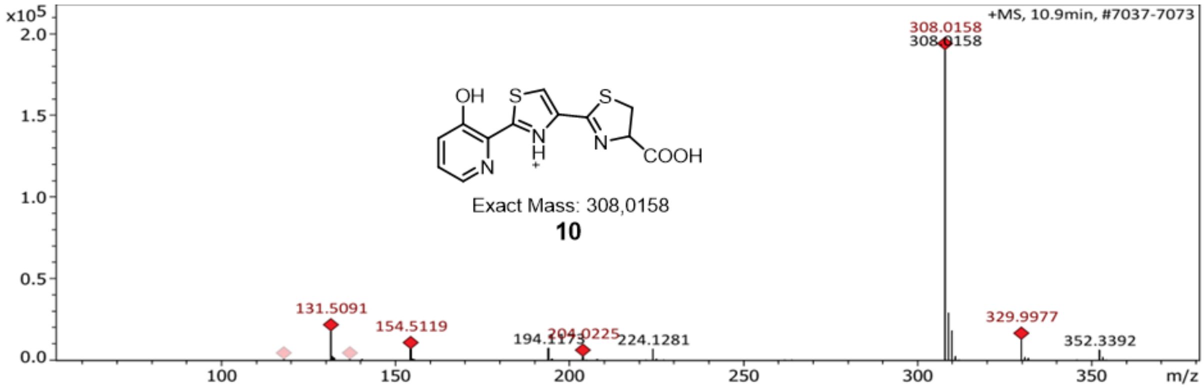

Figure S25. HR-ESI-MS spectrum of compound **10** $[M+H]^+$ (308.00158 m/z)

NMR-Data

Bilothiazoles A, B (**5**, **6**)

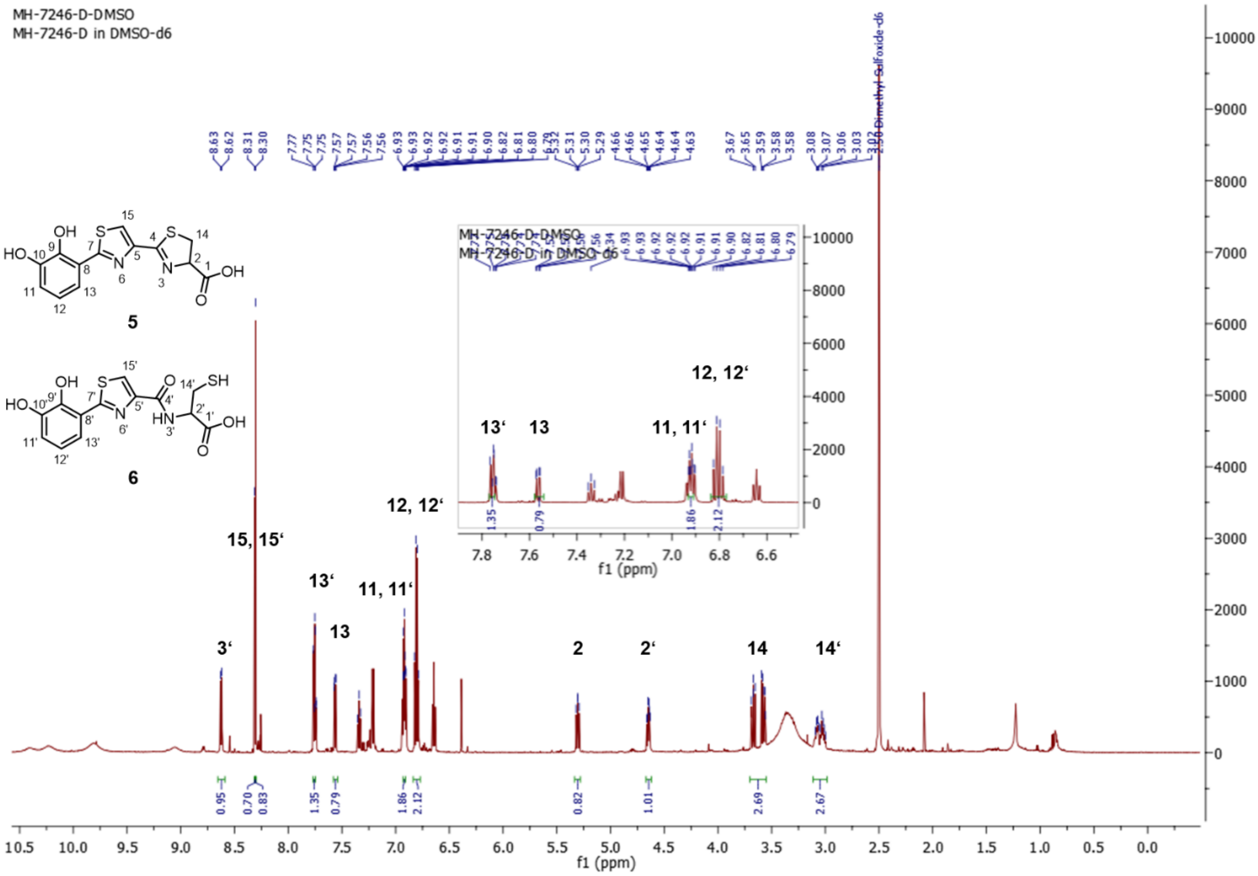

Figure S26. ^{1}H-NMR spectrum of a mixture of **5** and **6** in the ratio 4:5 in DMSO-d_6. The ^{1}H-signals belonging to the two molecules are numbered.

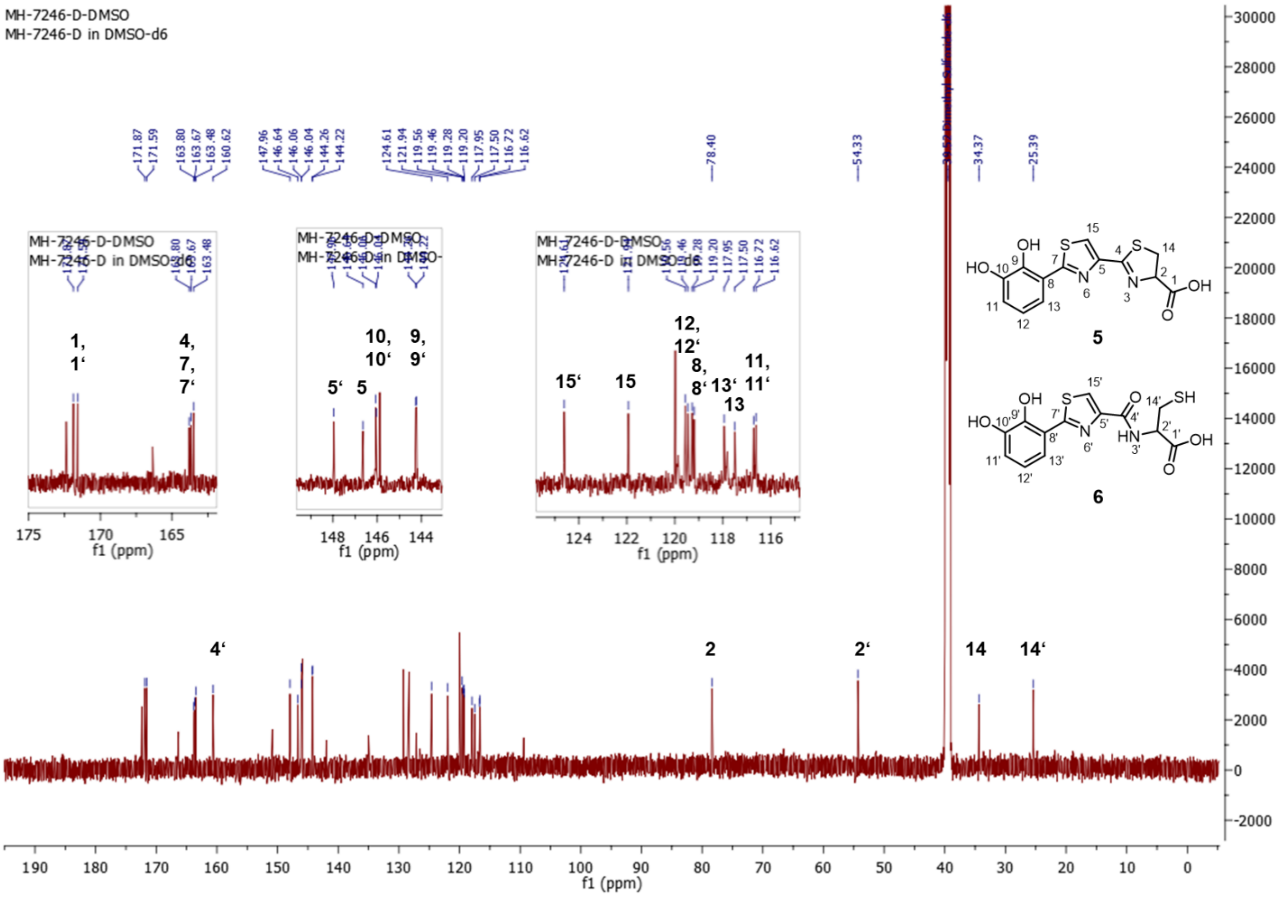

Figure S27. ^{13}C-NMR spectrum of a mixture of **5** and **6** in the ratio 4:5 in DMSO-d$_6$. The ^{13}C-signals belonging to the two molecules are numbered.

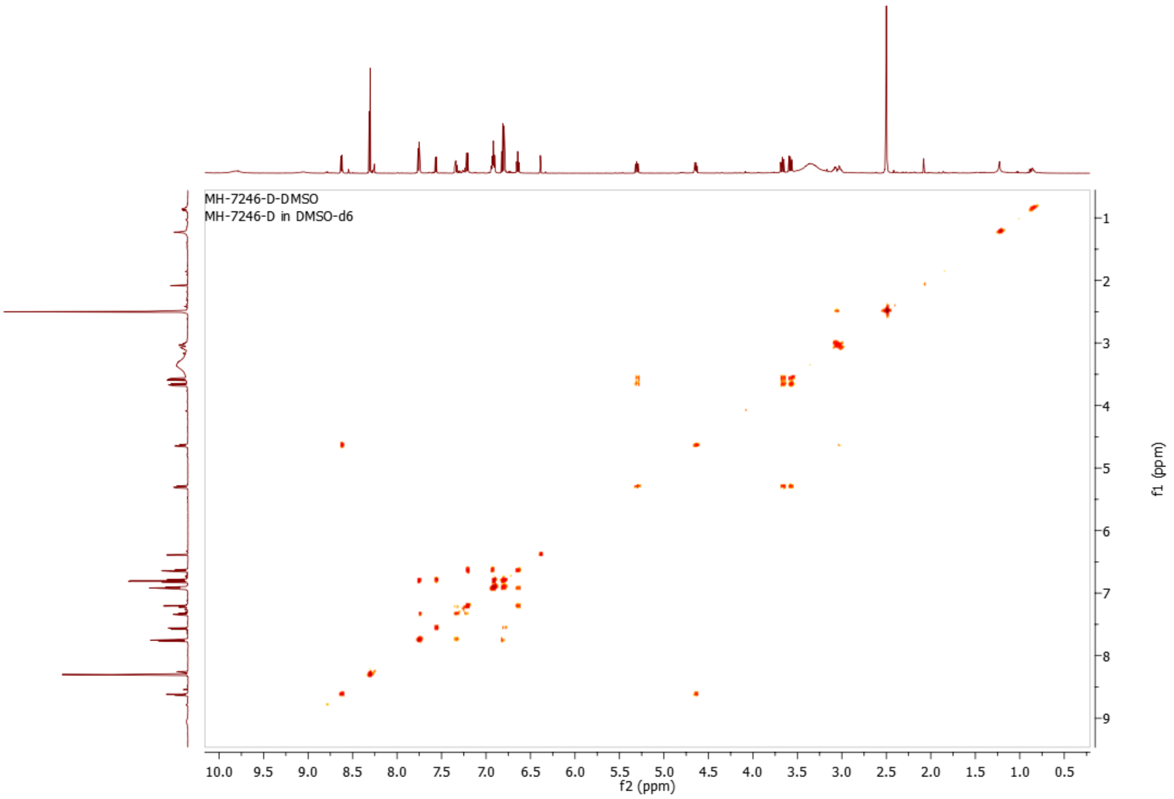

Figure S28. ^{1}H-^{1}H COSY spectrum of a mixture of **5** and **6** in the ratio 4:5 in DMSO-d_6.

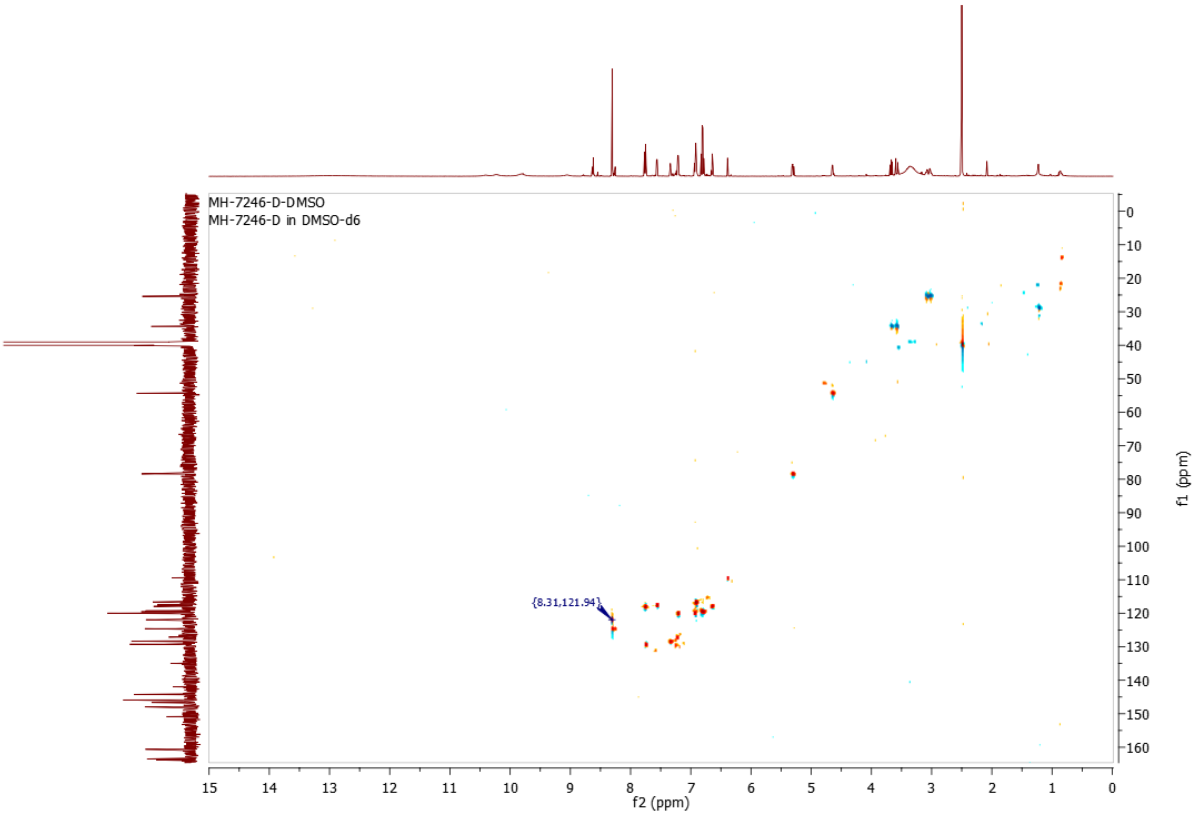

Figure S29. ^{1}H-^{13}C HMBC spectrum of a mixture of **5** and **6** in the ratio 4:5 in DMSO-d_6.

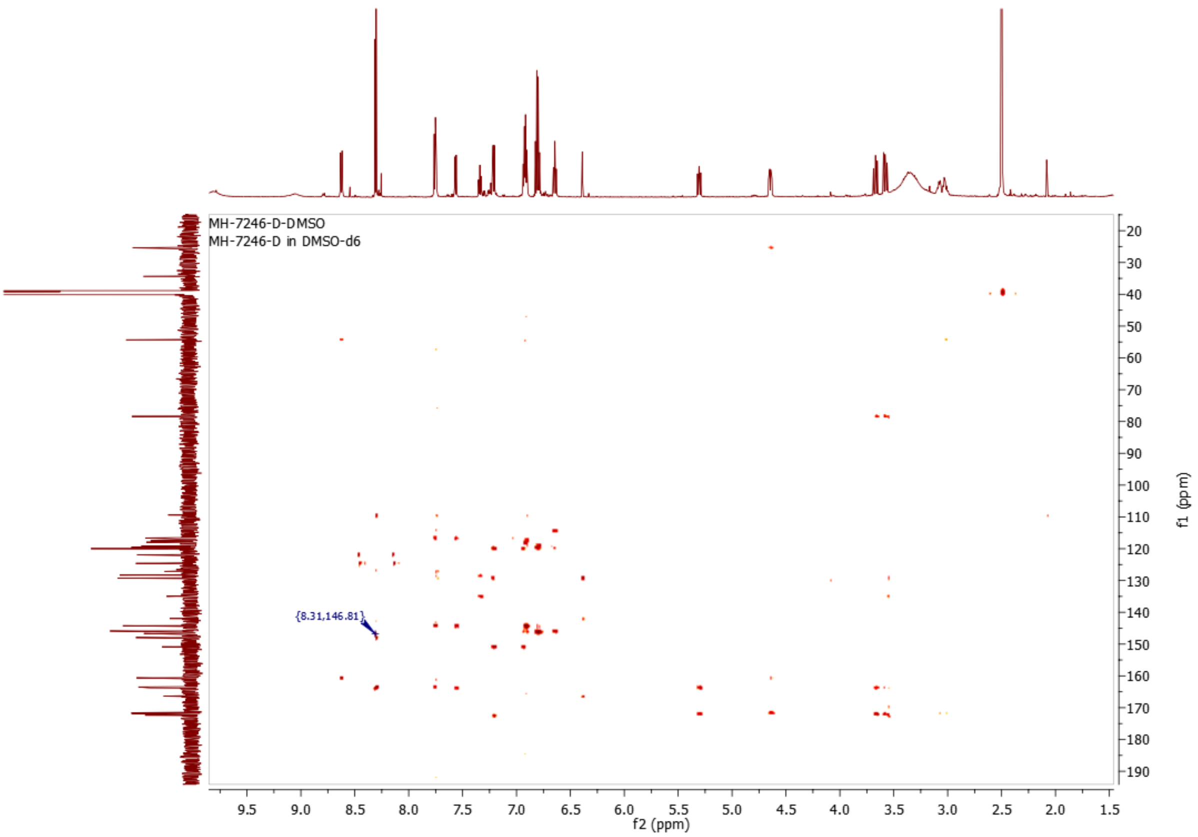

Figure S30. ^{13}C-^{13}C HMBC spectrum of a mixture of **5** and **6** in the ratio 4:5 in DMSO-d_6.

Bilothiazole C (**7**)

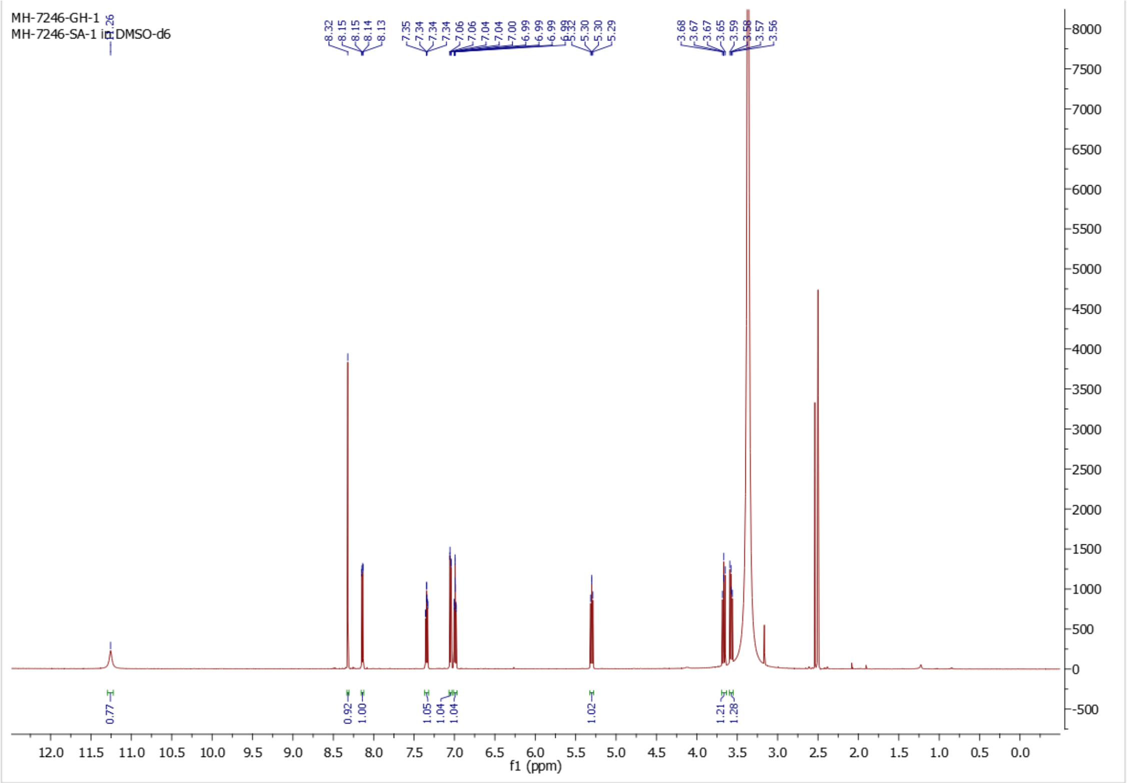

Figure S31. ^{1}H-NMR spectrum of **7** in DMSO-d_6.

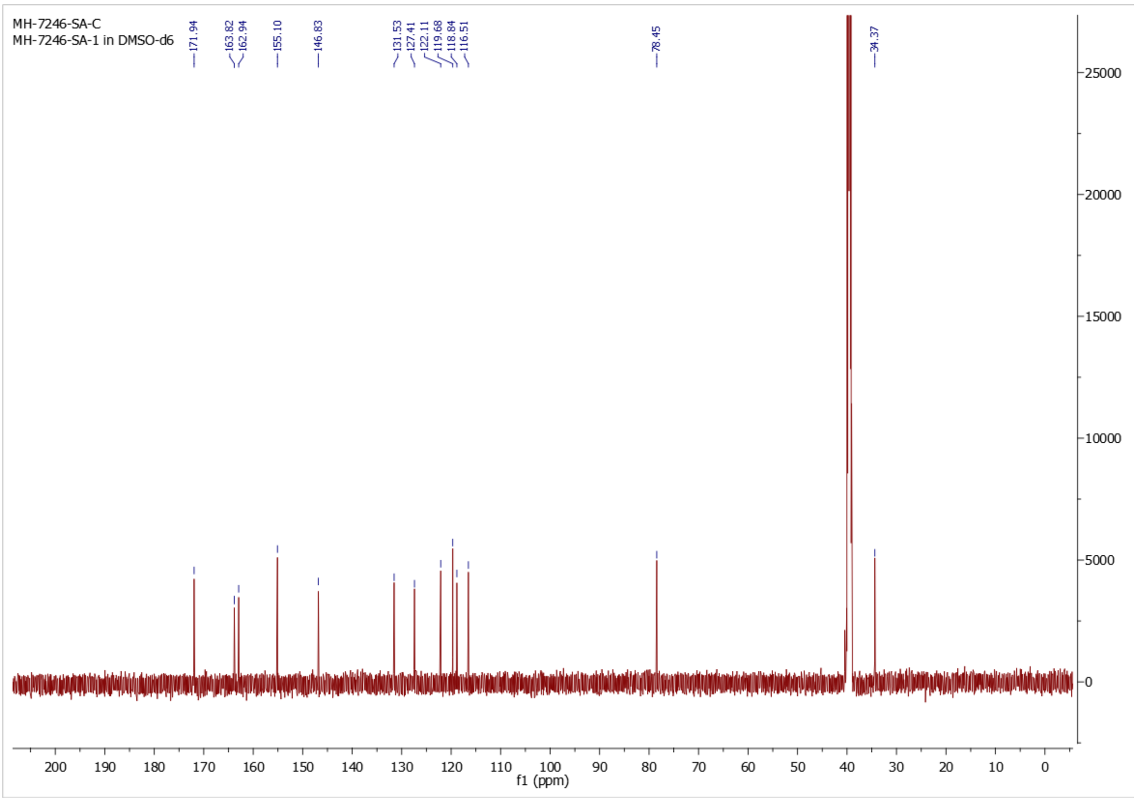

Figure S32. ^{13}C-NMR spectrum of **7** in DMSO-d_6.

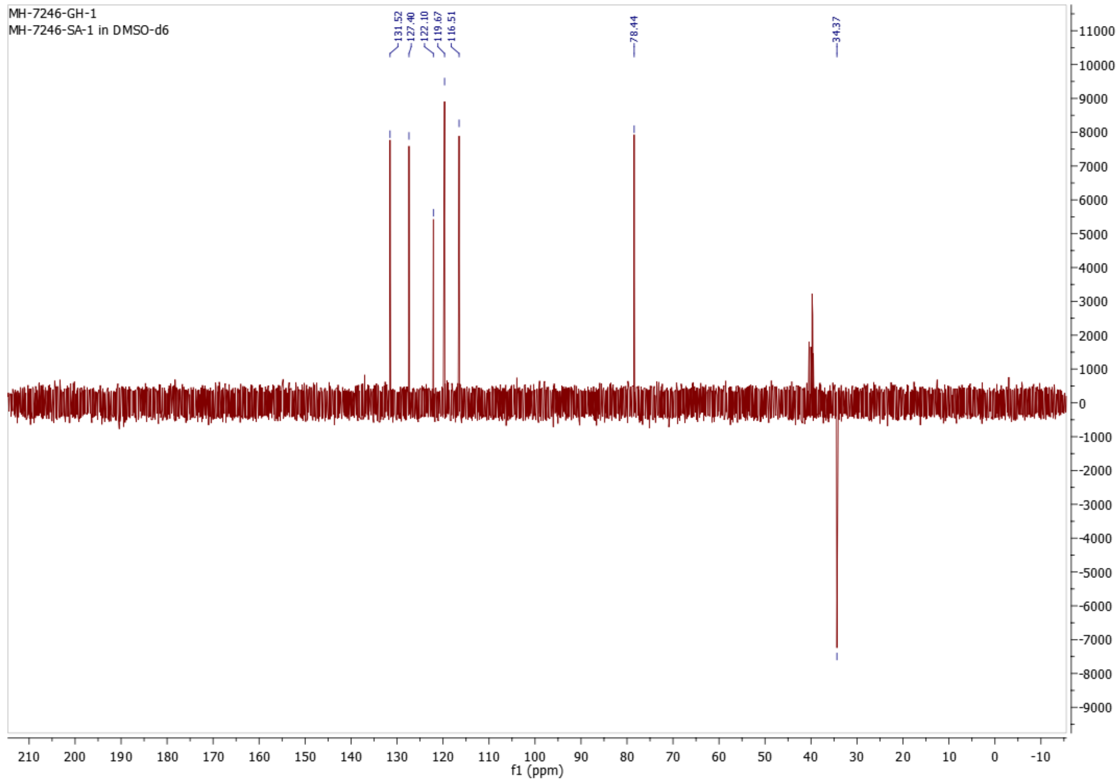

Figure S33. ^{13}C-DEPT spectrum of **7** in DMSO-d$_6$.

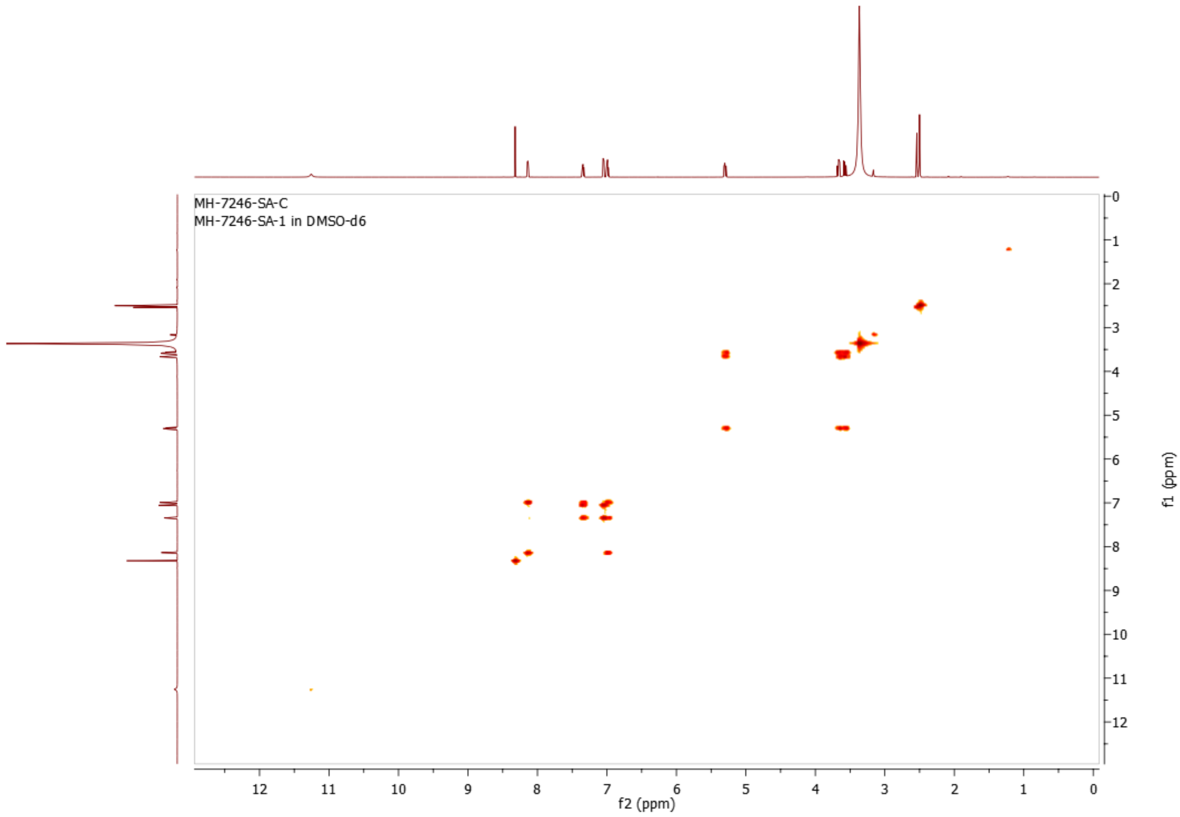

Figure S34. ^{1}H-^{1}H COSY spectrum of **7** in DMSO-d$_6$.

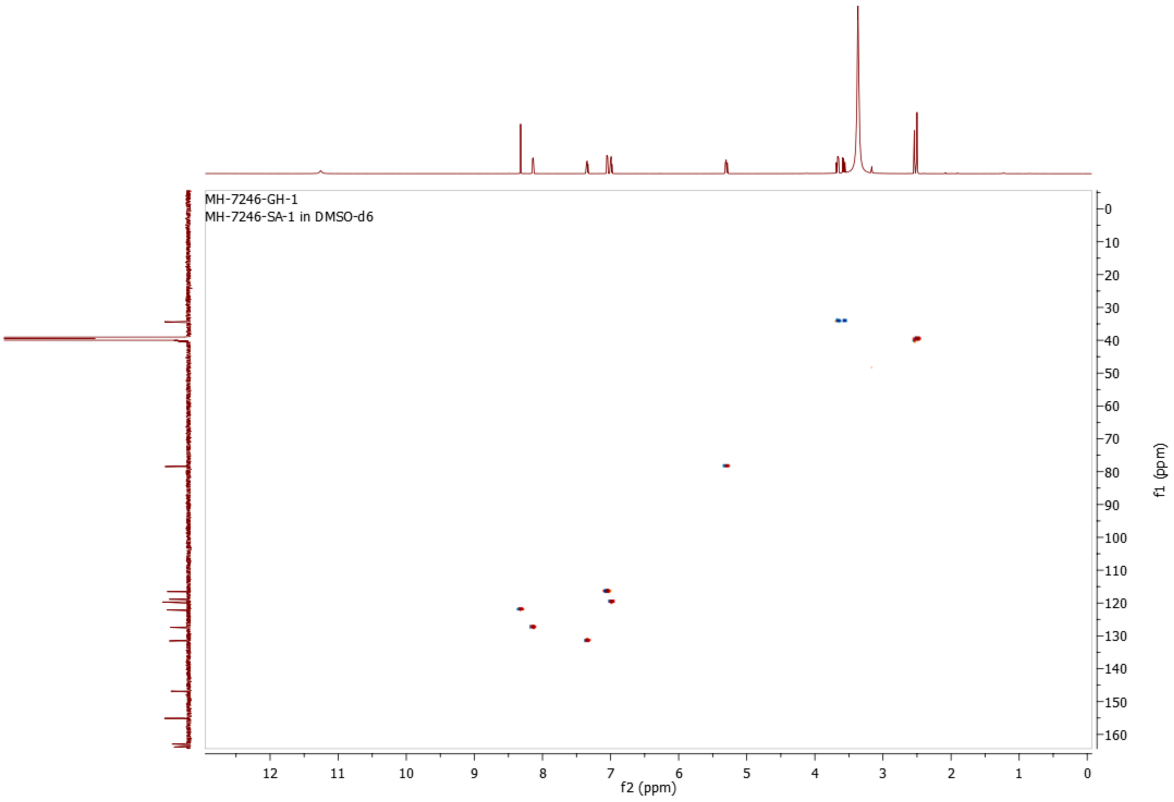

Figure S35. ^{1}H-^{13}C HSQC spectrum of **7** in DMSO-d$_6$.

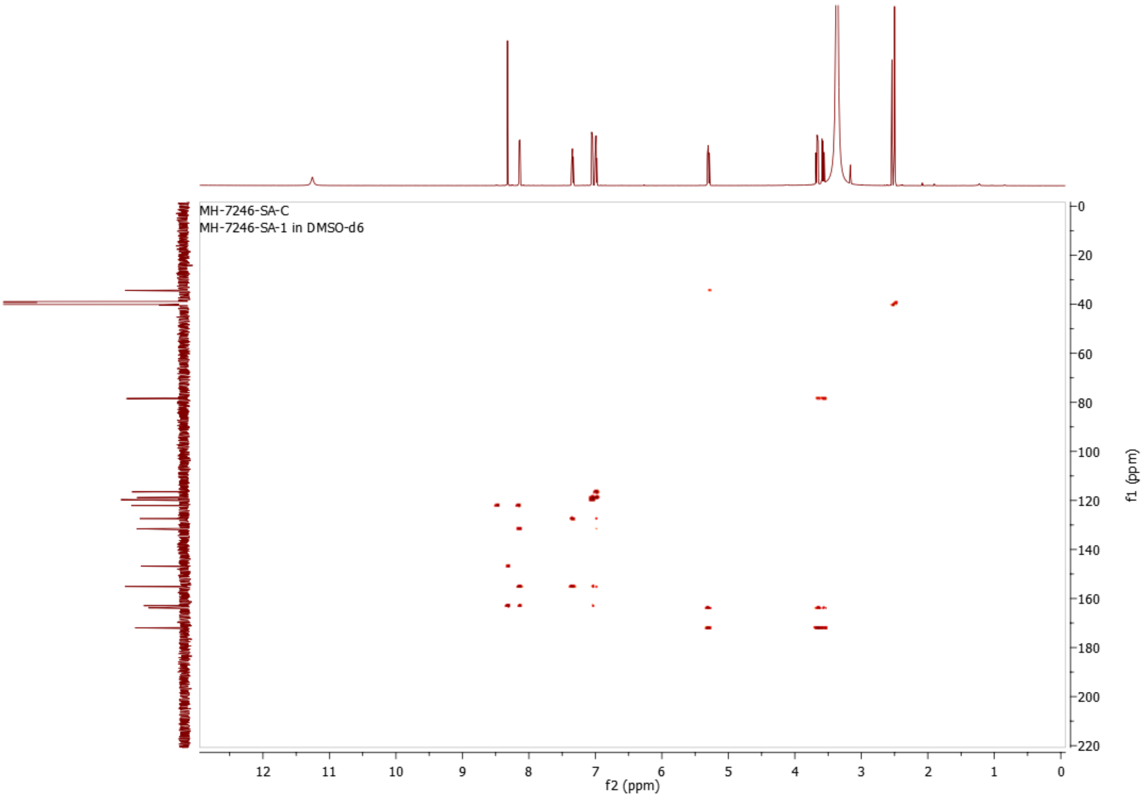

Figure S36. ^{1}H-^{13}C HMBC spectrum of **7** in DMSO-d$_6$.

Bilothiazole D (**8**)

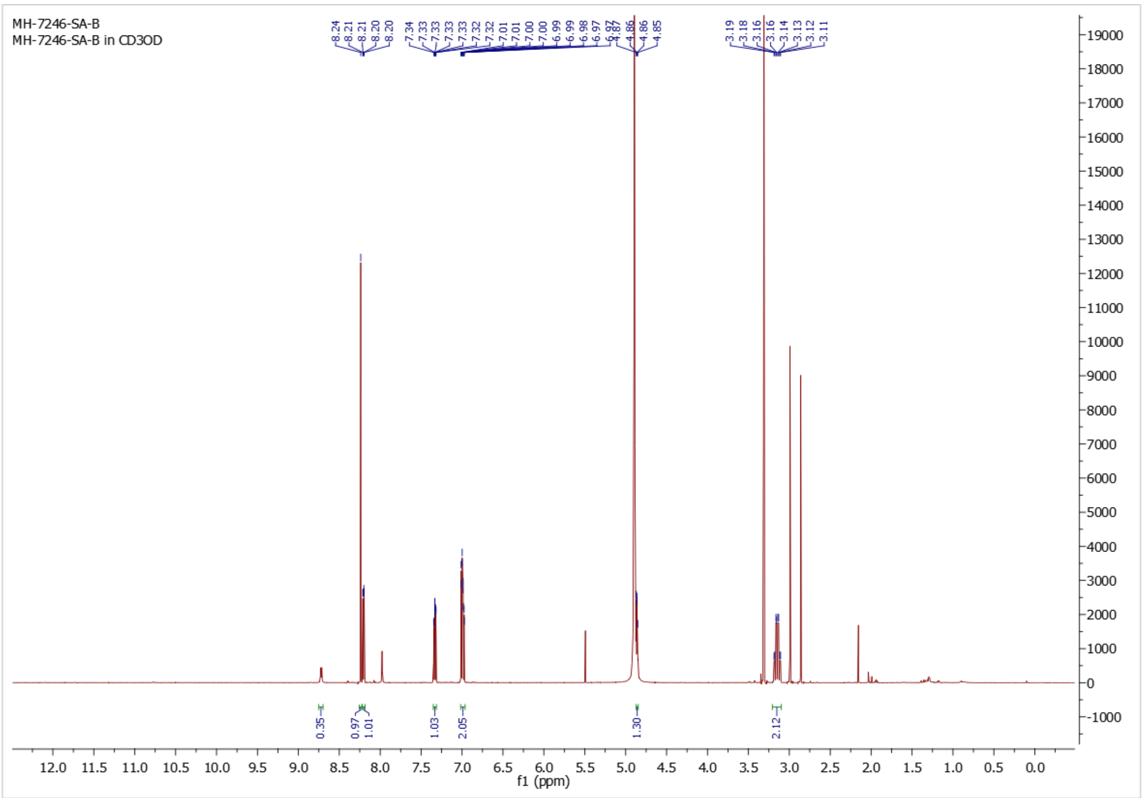

Figure S37. ^{1}H-NMR spectrum of **8** in CD_3OD.

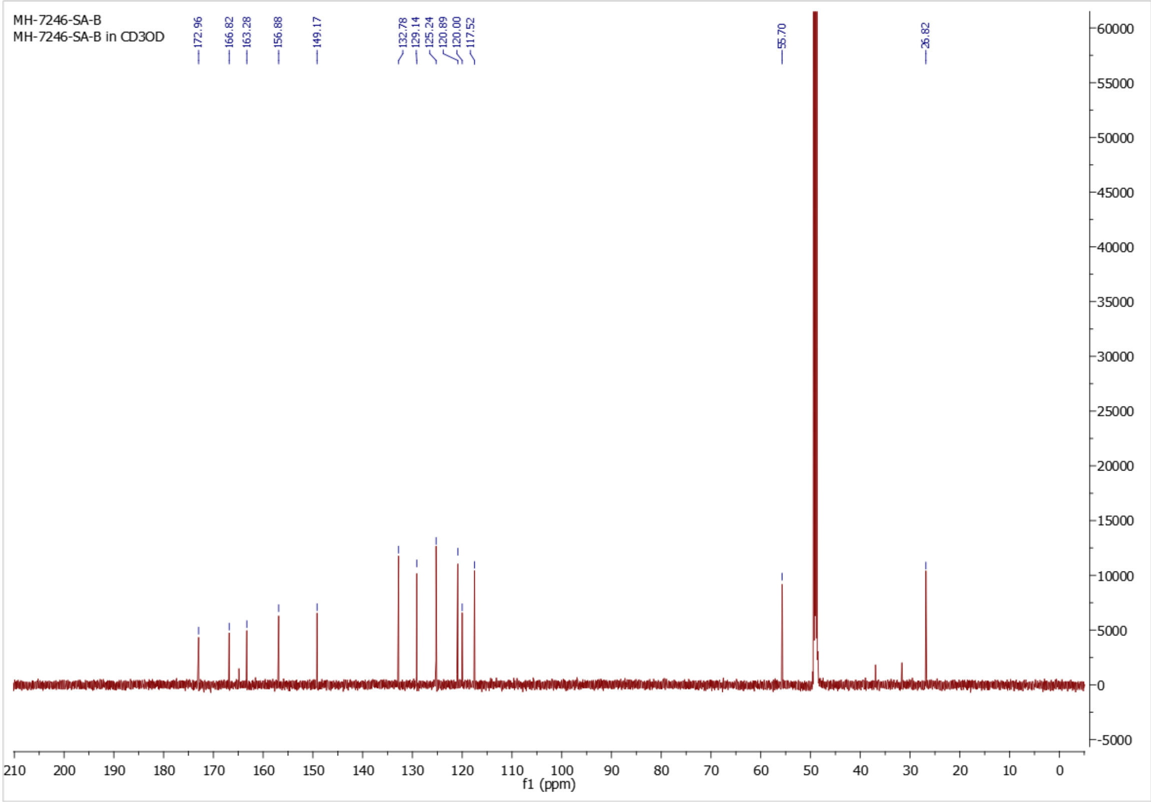

Figure S38. ^{1}H-NMR spectrum of **8** in CD_3OD.

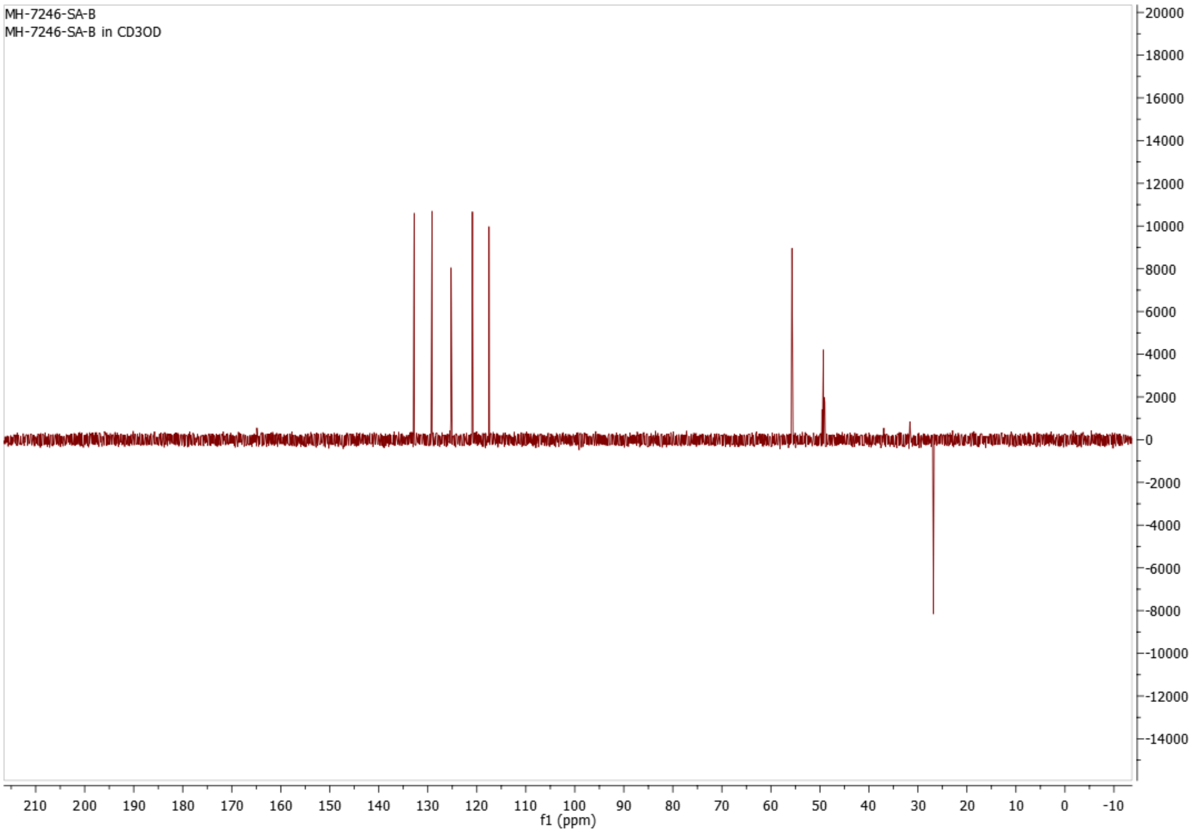

Figure S39. ^{13}C DEPT spectrum of **8** in CD_3OD.

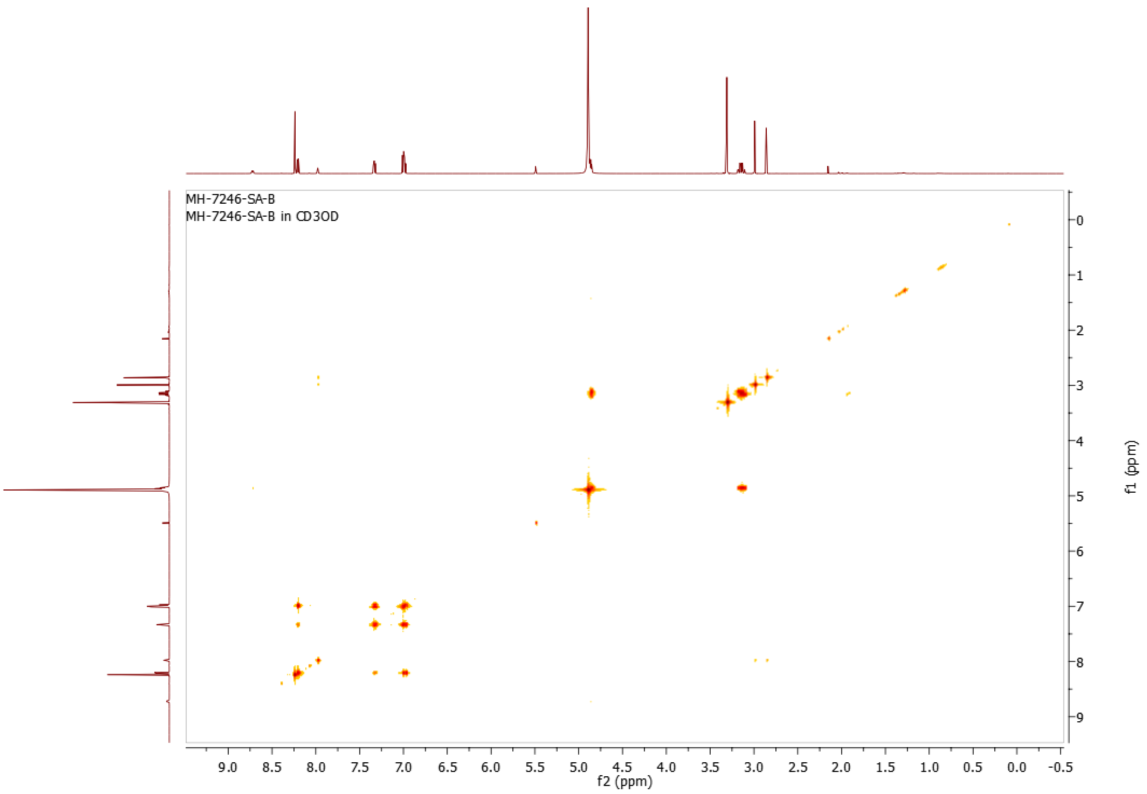

Figure S40. ^{1}H-^{1}H COSY spectrum of **8** in CD_3OD.

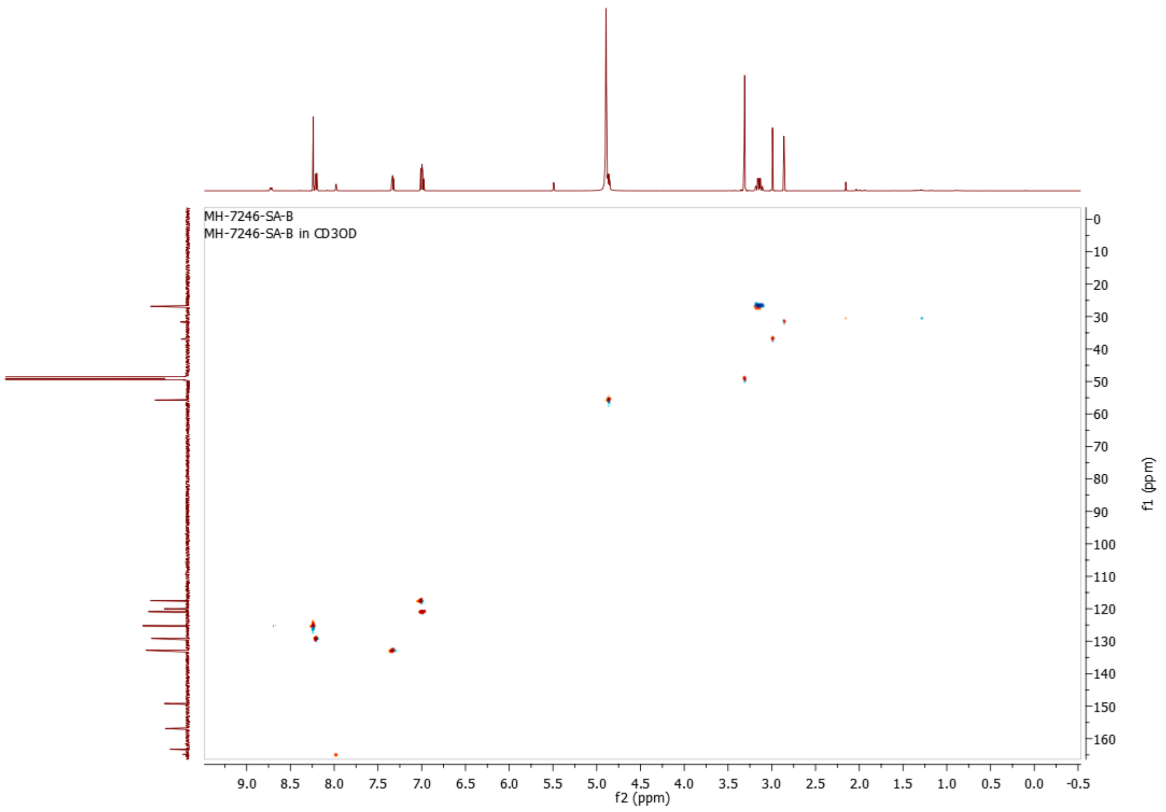

Figure S41. ^{1}H-^{13}C HSQC spectrum of **8** in CD_3OD.

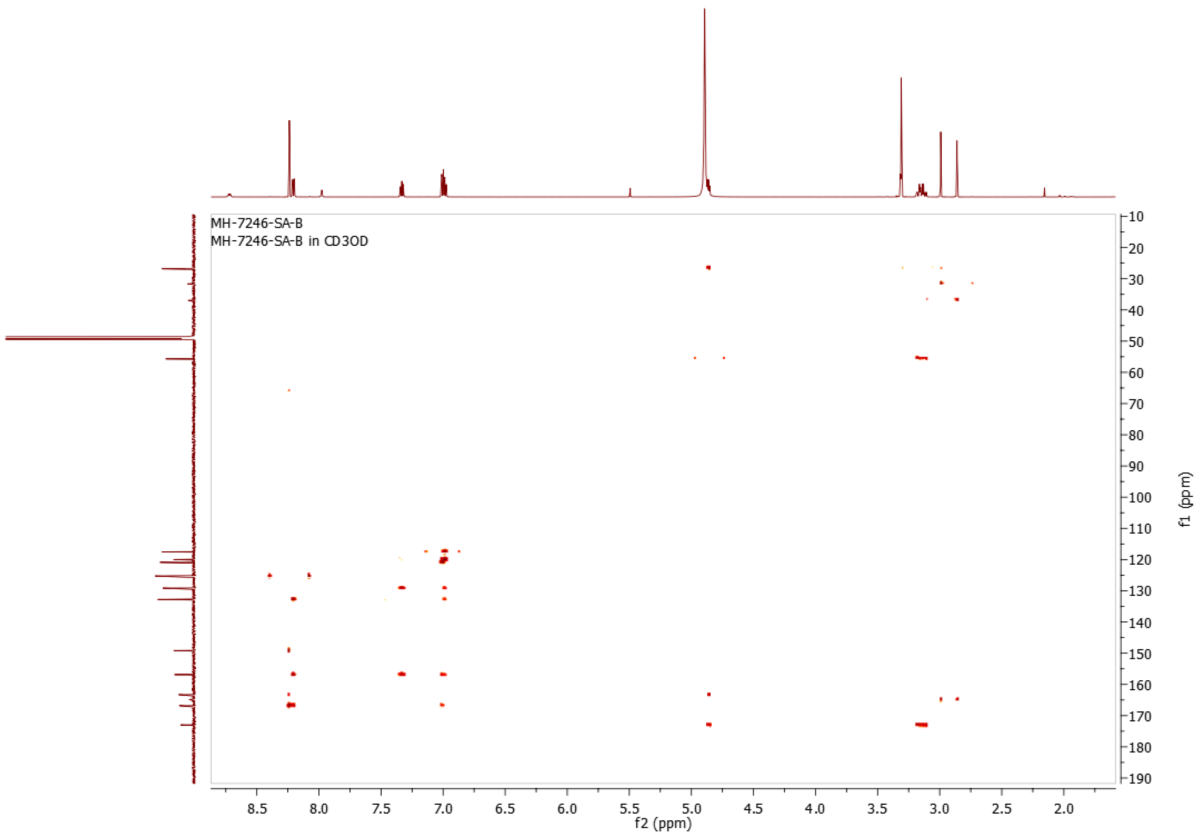

Figure S42. ^{1}H-^{13}C HMBC spectrum of **8** in CD_3OD.

Bilothiazole E (**9**)

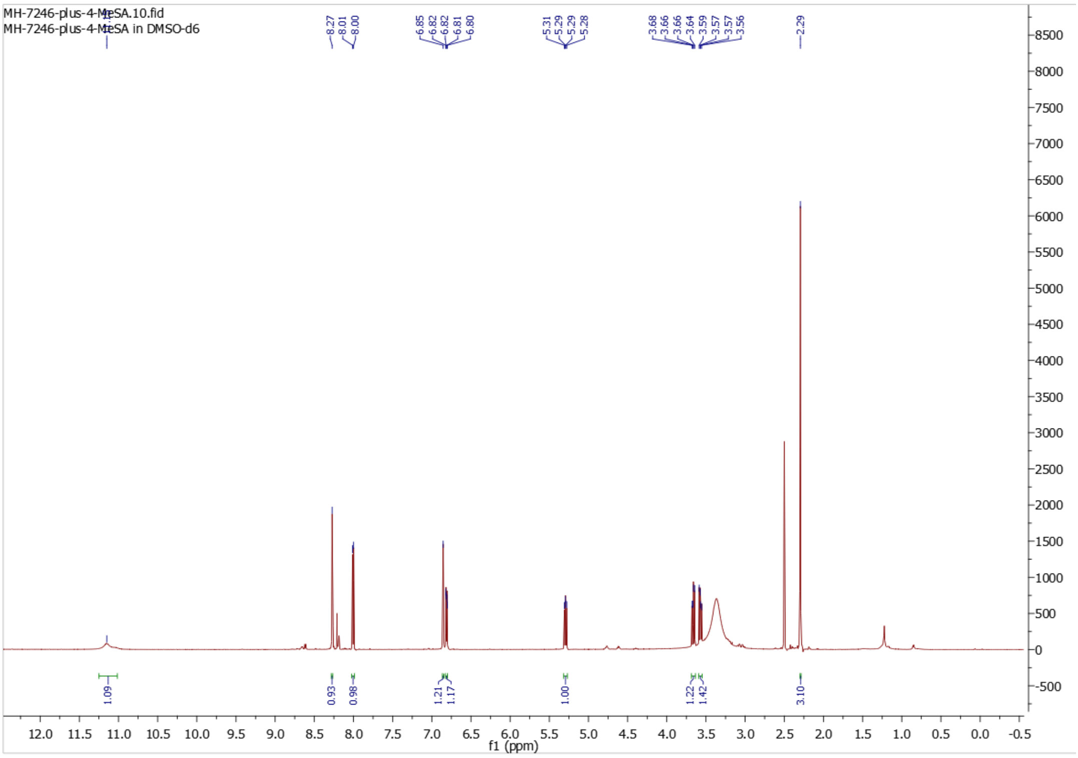

Figure S43. ^{1}H-NMR spectrum of **9** in DMSO-d_6.

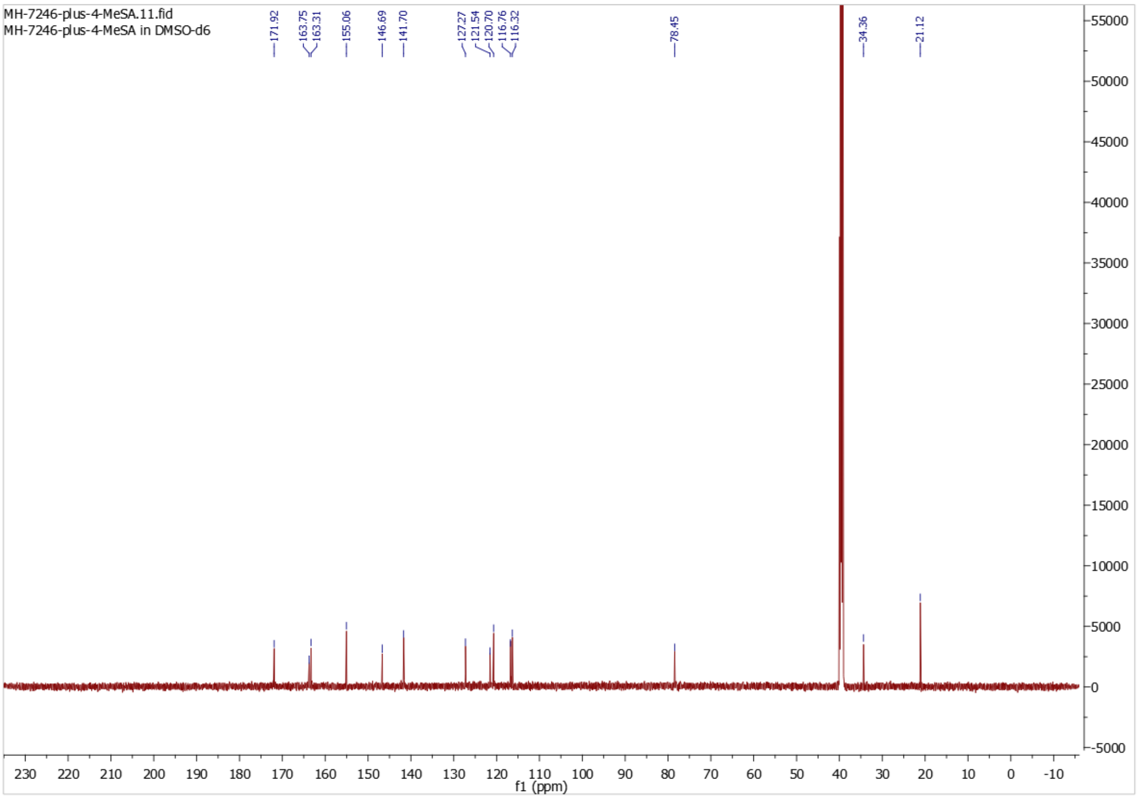

Figure S44. ^{13}C-NMR spectrum of **9** in DMSO-d_6.

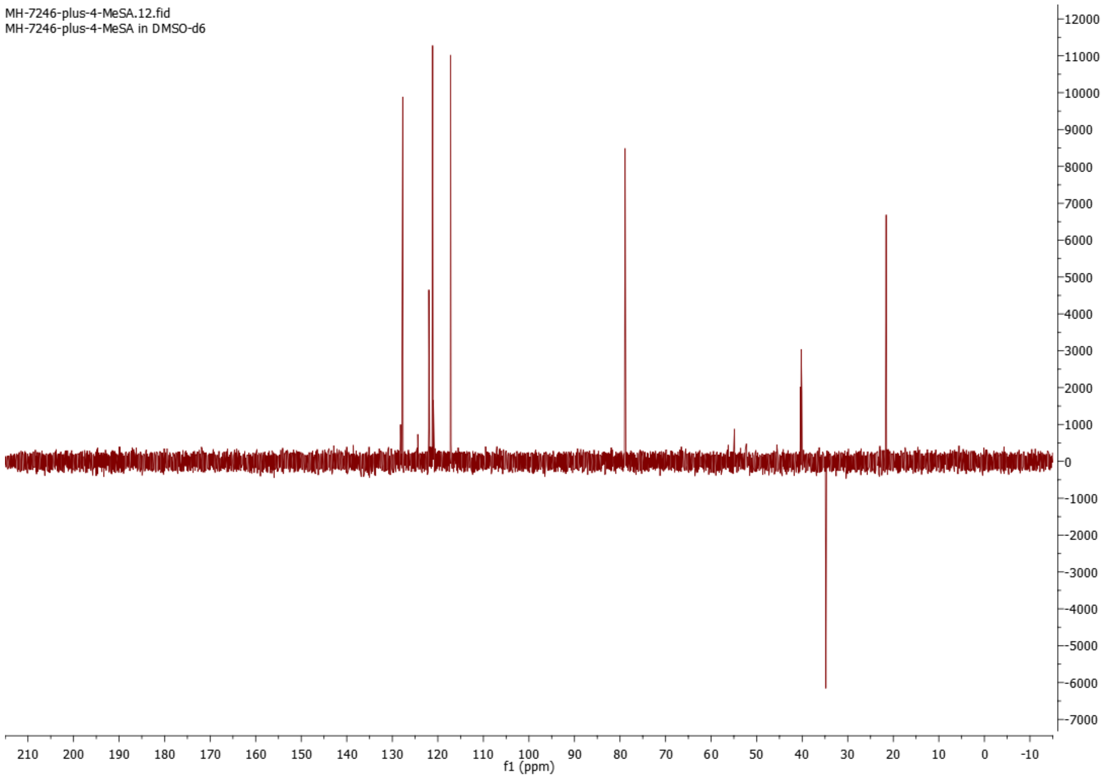

Figure S45. ^{13}C-DEPT spectrum of **9** in DMSO-d_6.

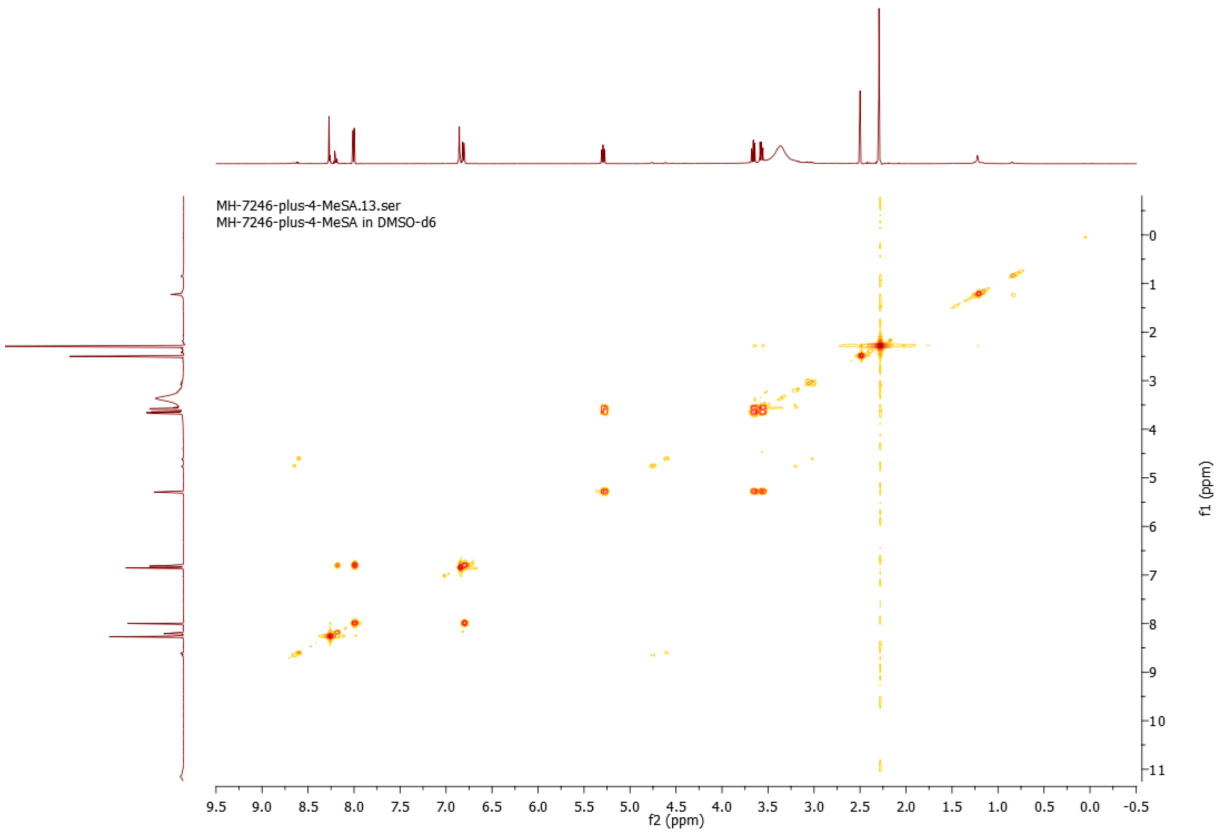

Figure S46. ^{1}H-^{1}H-COSY spectrum of **9** in DMSO-d_6.

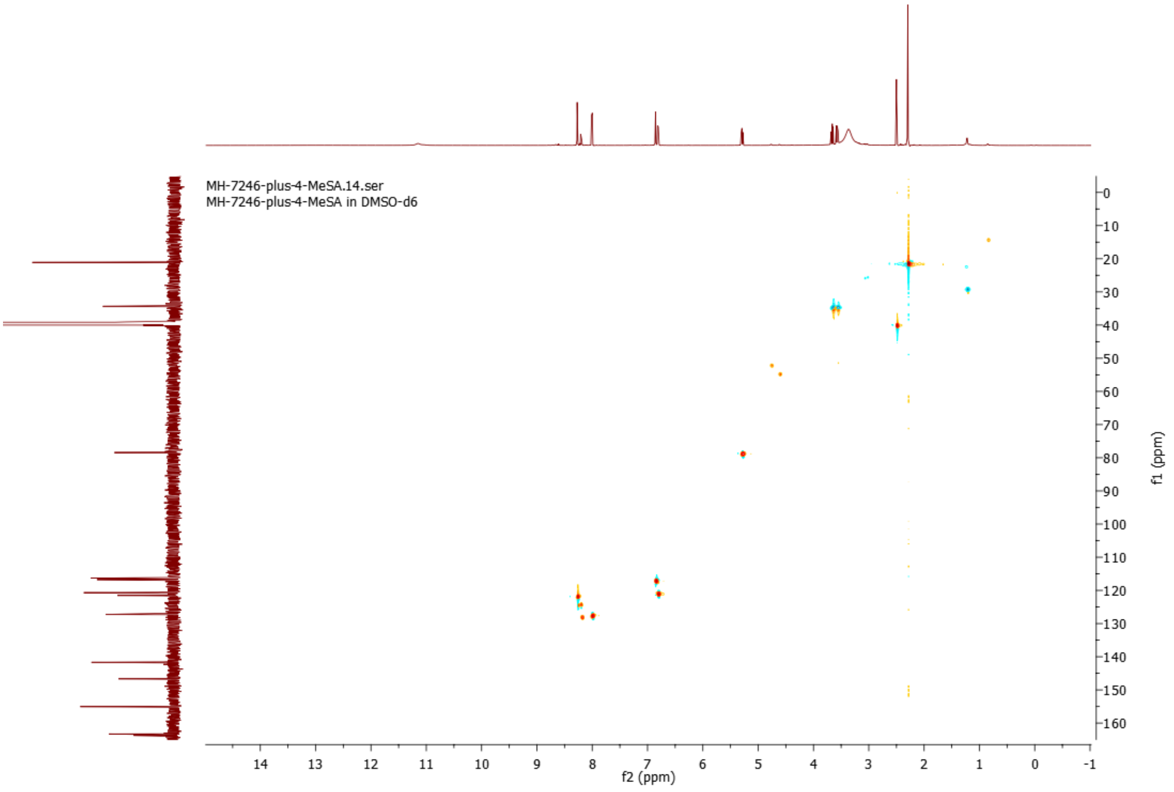

Figure S47. ^{1}H-^{13}C-HSQC spectrum of **9** in DMSO-d_6.

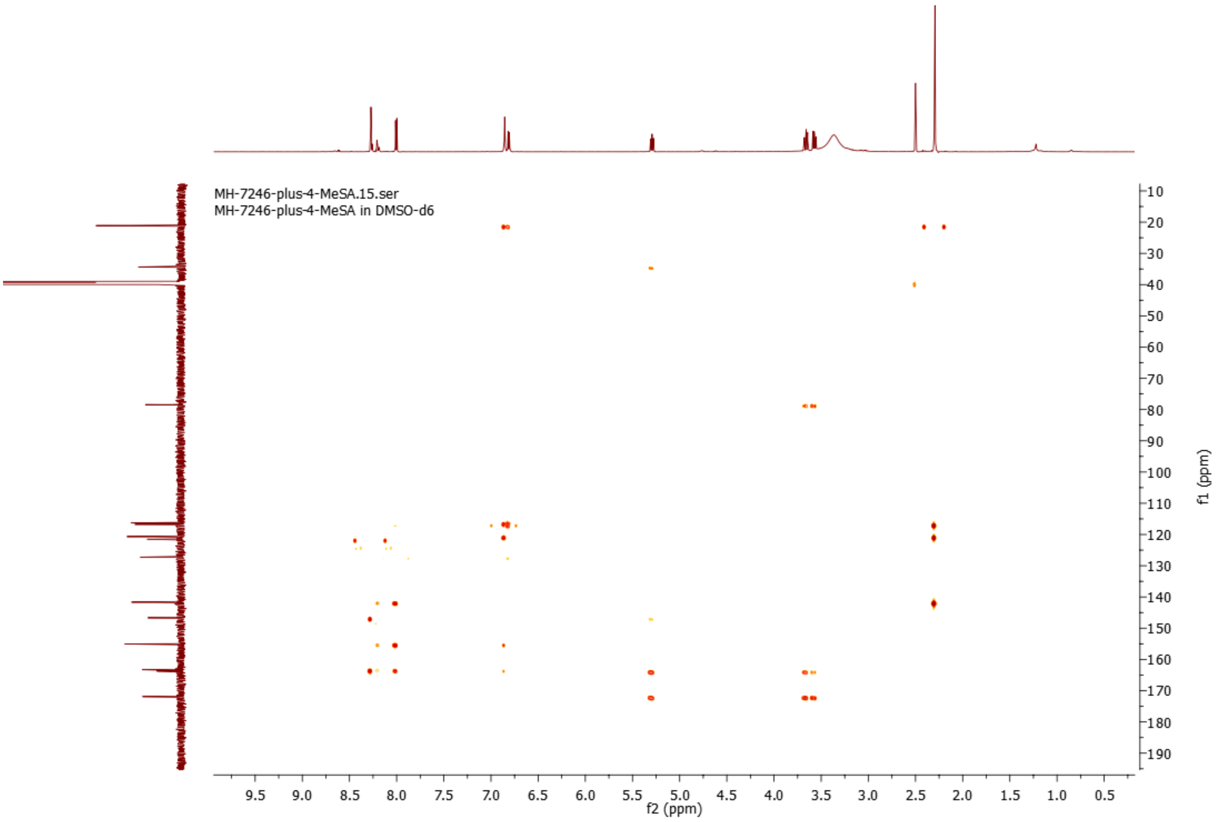

Figure S48. ^{1}H-^{13}C-HMBC spectrum of **9** in DMSO-d_6.

Bilothiazole F (**10**)

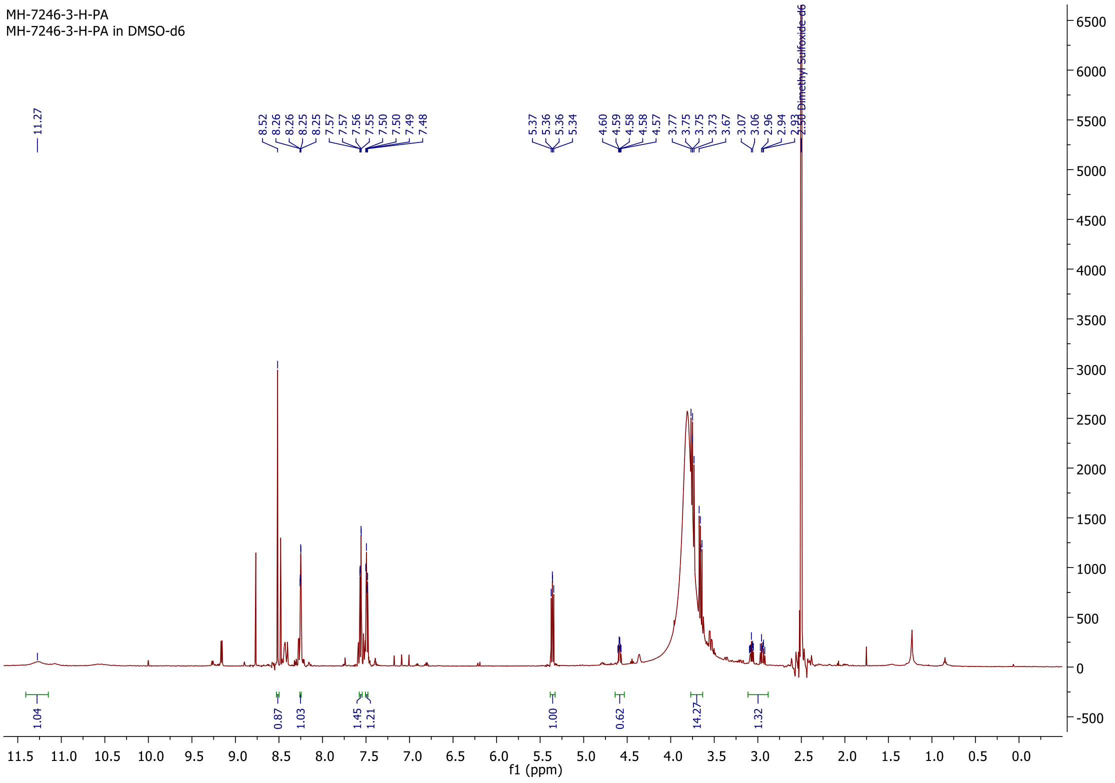

Figure S49. ^{1}H-NMR spectrum of **10** in DMSO-d6.

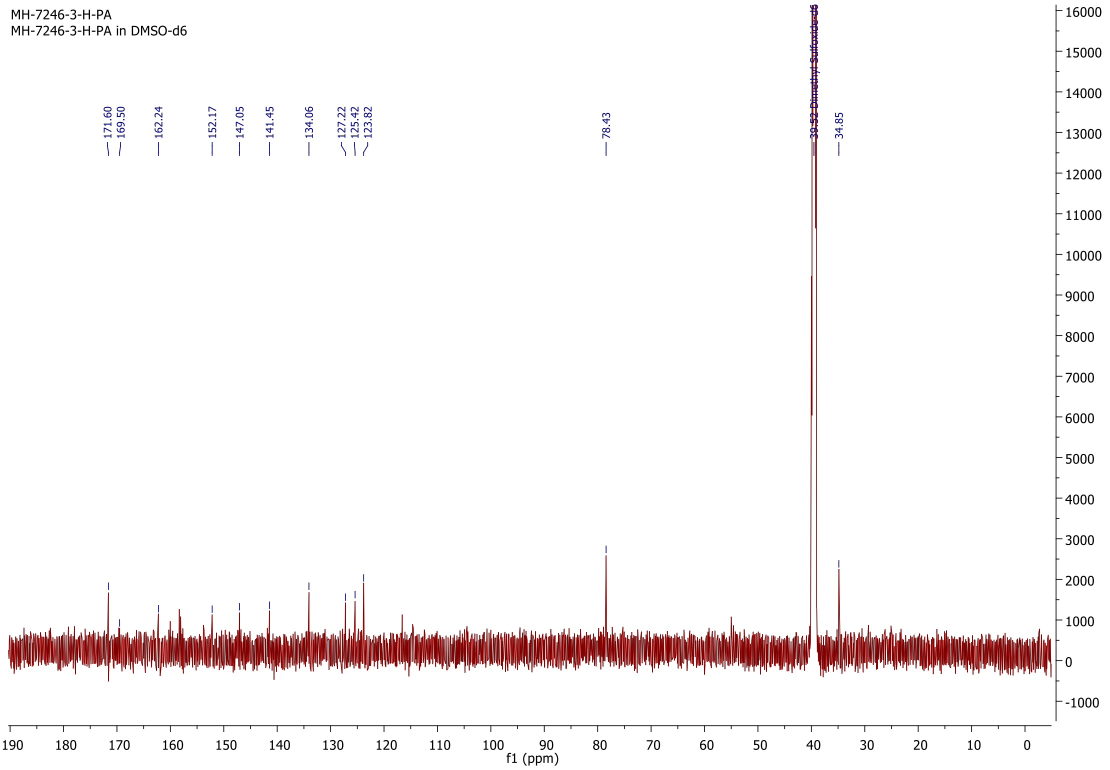

Figure S50. ^{13}C-NMR spectrum of **10** in DMSO-d6.

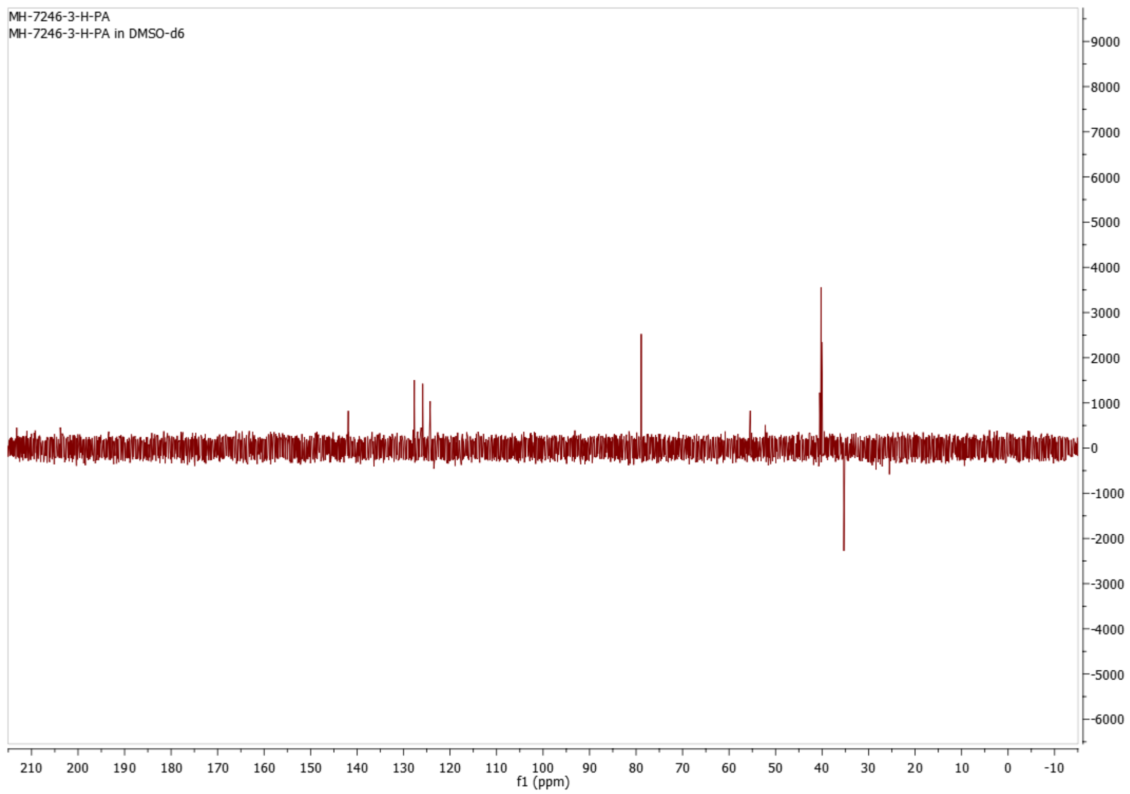

Figure S51. ^{13}C-DEPT spectrum of **10** in DMSO-d$_6$.

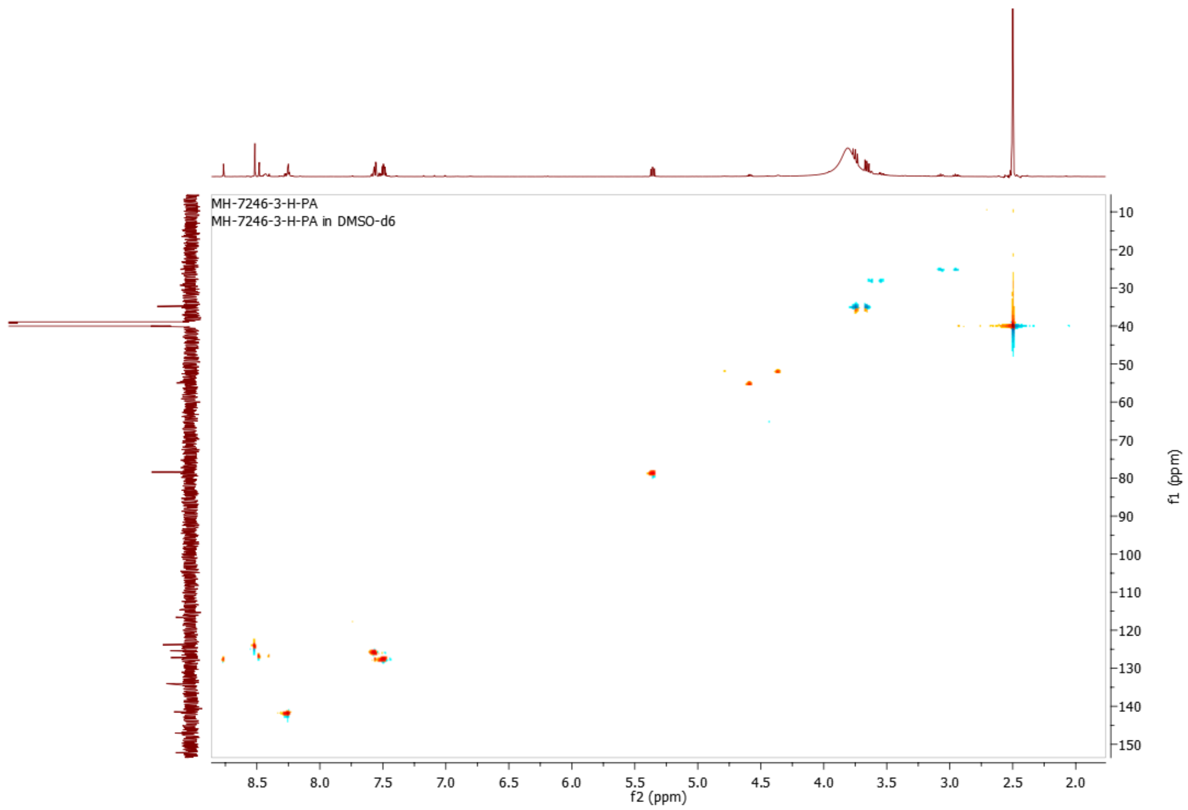

Figure S52. ^{1}H-^{13}C HSQC spectrum of **10** in DMSO-d$_6$.

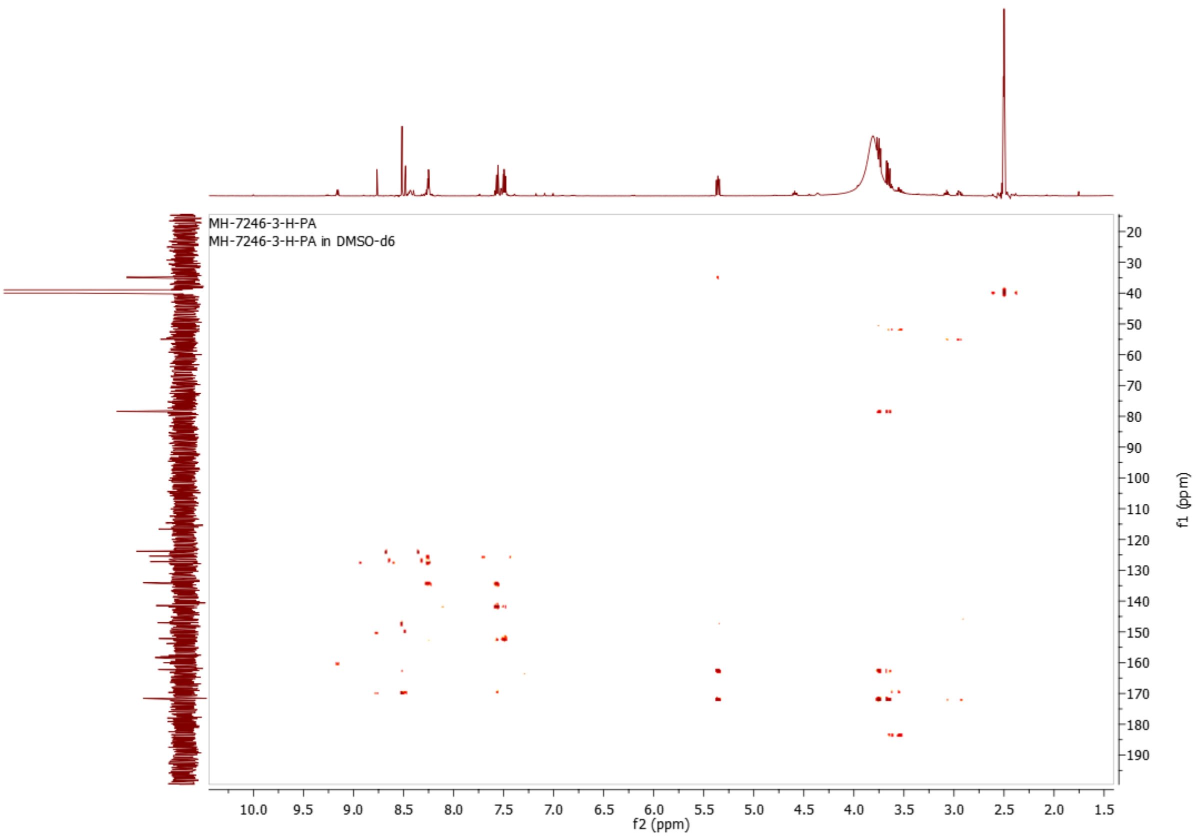

Figure S53. ^{1}H-^{13}C HMBC spectrum of **10** in DMSO-d_6.

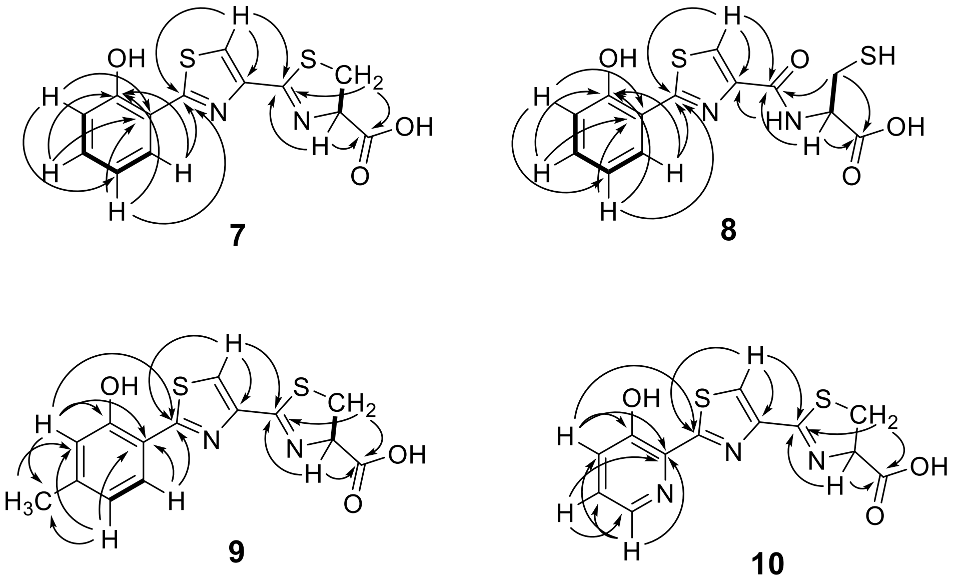

Figure S54. Chemical structures of bilothiazoles C (**7**), D (**8**), E (**9**) and F (**10**) with key COSY (bold) and HMBC (arrows) correlations.

Supplementary References

(1) Pfeifer, B. A.; Admiraal, S. J.; Gramajo, H.; Cane, D. E.; Khosla, C. Biosynthesis of complex polyketides in a metabolically engineered strain of E. coli. *Science* **2001**, *291* (5509), 1790–1792. DOI: 10.1126/science.1058092.

(2) Lautenschläger, N.; Popp, P. F.; Mascher, T. Development of a novel heterologous β-lactam-specific whole-cell biosensor in Bacillus subtilis. *J. Biol. Eng.* **2020**, *14*, 21. DOI: 10.1186/s13036-020-00243-4.

(3) Milzarek, T. M.; Stevanovic, M.; Milivojevic, D.; Vojnovic, S.; Iliasov, D.; Wolf, D.; Mascher, T.; Nikodinovic-Runic, J.; Gulder, T. A. M. Antibiotic Potential of the Ambigol Cyanobacterial Natural Product Class and Simplified Synthetic Analogs. *ACS Infect. Dis.* **2023**, *9* (10), 1941–1948. DOI: 10.1021/acsinfecdis.3c00232.

(4) Duell, E. R.; D'Agostino, P. M.; Shapiro, N.; Woyke, T.; Fuchs, T. M.; Gulder, T. A. M. Direct pathway cloning of the sodorifen biosynthetic gene cluster and recombinant generation of its product in E. coli. *Microb. Cell Fact.* **2019**, *18* (1), 32. DOI: 10.1186/s12934-019-1080-6.
